# Supplementary material for: Genome and Secretome Analysis of Staphylotrichum longicolleum DSM105789 Cultured on Agro-Residual and Chitinous Biomass
Source: Microorganisms. 2021 Jul 25;9(8):1581. doi: 10.3390/microorganisms9081581 (PMC8398502; doi:10.3390/microorganisms9081581)
Supplement: Supplementary file 1 [file microorganisms-09-01581-s001.zip › 3/MicroorganismsFW57_S3.pdf]

microorganisms

MDPI

Supplementary Material

Genome and Secretome Analysis of *Staphylotrichum longicolleum* DSM105789 Cultured on Agro-Residual and Chitinous Biomass †

Arslan Ali [1,2,3], Bernhard Ellinger [4], Sophie C. Brandt [5], Christian Betzel [1], Martin Rühl [6], Carsten Wrenger [1,7], Hartmut Schlüter [1,3], Wilhelm Schäfer [5], Hévila Brognaro [1,7] and Martin Gand [5,6,*]

[1] Institute of Biochemistry and Molecular Biology, University of Hamburg, Martin Luther King Platz 6, 20146 Hamburg, Germany; arslanali1986@gmail.com (A.A.); Christian.Betzel@uni-hamburg.de (C.B.); cwrenger@icb.usp.br (C.W.); ; hschluet@uke.de (H.S.); hevila.brognaro@chemie.uni-hamburg.de (H.B.)
[2] Dr Panjwani Center for Molecular Medicine and Drug Research, International Center for Chemical and Biological Sciences, University of Karachi, University Road, Karachi 75270, Pakistan
[3] Institute of Clinical Chemistry and Laboratory Medicine, Diagnostic Center, Section Mass Spectrometry & Proteomics, Campus Research, Martinistr. 2, N27, 20246 Hamburg, Germany
[4] Department ScreeningPort, Fraunhofer Institute for Translational Medicine and Pharmacology ITMP, Schnackenburgallee 114, 22525 Hamburg, Germany; Bernhard.Ellinger@itmp.fraunhofer.de
[5] Department of Molecular Phytopathology, Biocenter Klein Flottbek, University of Hamburg, Ohnhorststr. 18, 22609 Hamburg, Germany; sophie.brandt@live.de (S.C.B.); wilhelm.schaefer@gmail.com (W.S.)
[6] Institute of Food Chemistry and Food Biotechnology, Department Biology and Chemistry, Justus Liebig University Giessen, Heinrich-Buff-Ring 17, 35392 Gießen, Germany; Martin.Ruehl@lcb.Chemie.uni-giessen.de
[7] Biomedical Science Institute, University of São Paulo, Av. Lineu Prestes, 2415, São Paulo 05508-900, Brazil
* Correspondence: martin.gand@lcb.chemie.uni-giessen.de; Tel.: +49-641-99-34912
† This article is dedicated to Peter Grunwald and Frieder Schauer—both were outstanding scientists.

Supplementary Data 1

The ITS region amplified and sequenced with primers ITS1 and ITS4 (White et al., 1990). Sequencing reactions were performed using ABI Dye Terminator technology according to the manufacturer's instructions (Applied Biosystems, Foster City, CA, USA). The ITS sequence has been deposited in GenBank (accession number MG098702).

MG098702.1 *Staphylotrichum longicolleum* DSM105789 (formerly known as *Humicola* sp. strain FW57) internal transcribed spacer 1, partial sequence; 5.8S ribosomal RNA gene and internal transcribed spacer 2, complete sequence; and large subunit ribosomal RNA gene, partial sequence

CGTTACAGAGTTGCAAAACTCTCTAAACCATTGTGAACGTTACCTAAACCGTTGCTTCGGCGGGCGGCCC

GGGTCCTTCCCGGCGCCCCTCGGCCCTCGCGGGCGCCCGCCGGAGGTAAACCAAACTATTGCATTGTATG

GCCTCTCTGAGTCTTCTGTACTGAATAAGTCAAAACTTTCAACAACGGATCTCTTGGTTCTGGCATCGAT

GAAGAACGCAGCGAAATGCGATAAGTAATGTGAATTGCAGAATTCAGTGAATCATCGAATCTTTGAACGC

ACATTGCGCCCGCCAGTATTCTGGCGGGCATGCCTGTTCGAGCGTCATTTCAACCATCAAGCCCCGGGCT

TGTGTTGGGGACCTGCGGCTGCCGCAGGCCCTGAAATGCAGTGGCGGGCTCGCTGTCACTCCGAGCGTAG

TAGTTACATCTCGCTCTGGGCGTGCTGCGGGTTCCGGCCGTTAAAAGCCTTATTTACCCAAGGTTGACCT

CGATCAGGTAGGAAGACCCGCTGAACTTAAGCATAACAATAAGCGG

Supplementary Figures and Tables

Supplementary Figures

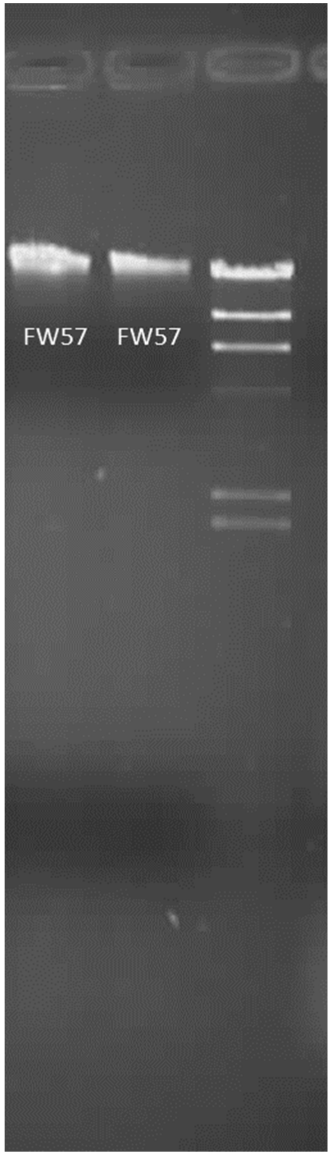
FW57
FW57

Supplementary Figure S1: Agarose gel showing genomic DNA isolated from *Staphylotrichum longicolleum* DSM105789 FW57. The DNA was isolated using the CTAB method and was analyzed on 0.8% (w/v) agarose gel electrophoresis. We mixed 5 µL DNA with 6 x loading buffer (0.25% (w/v) xylene cyanol, 0.25% (w/v) bromophenol blue, 30% (v/v) glycerol in Tris-borate-EDTA) at a 1:6 ratio, loaded the gel and separated at 80 V for 60 min. The GeneRuler 1 kb Plus DNA Ladder (Thermo Fischer Scientific, Darmstadt, Germany) was used as a marker. The DNA was stained with 1% ethidium bromide for 15 min. The gel was documented in a UV transilluminator (SynGene Genius, Bio Imaging System).

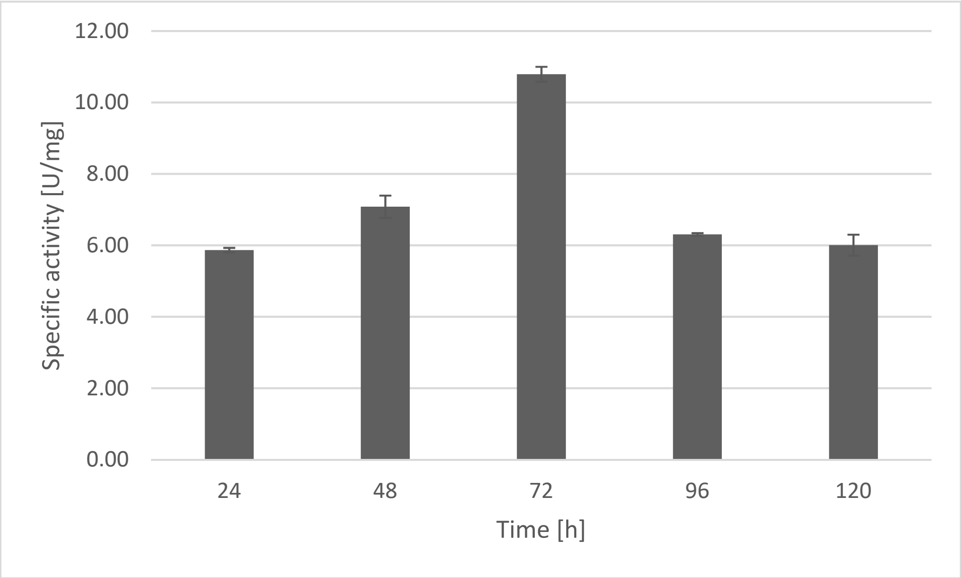

Supplementary Figure S2: Specific chitinase activity of the FW57 supernatants on chitin as a substrate. The fungus was cultivated in YPD medium (28 °C shaking at 145 rpm in the dark) for up to 5 days followed by centrifugation (3250 × g, 20 min, 4 °C) to obtain the cultivation supernatant. Released *N*-acetylglucosamine was determined using Schales reagent (Ferrari et al. 2014).

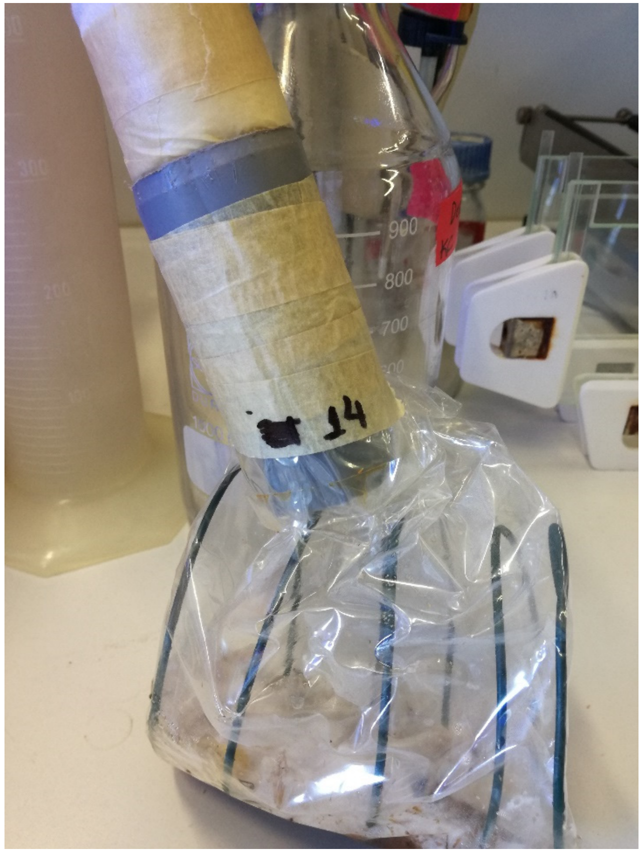

Supplementary Figure S3: Solid-state fermentation of the FW57 strain on agro-residual biomass (SCB) after 24 days of cultivation.

Supplementary Tables

Supplementary Table S1. CAZyme analysis of fungal isolate FW57. Coding regions were annotated with the standalone version of the dbCAN annotation tool and BLASTP (Zhang et al. 2014).

GeneID	Feature	Contig	Start	Stop	Strand	PFAM	CAZyme
NEMBOFW57_001206	CDS	scaffold_1	3916522	3918513	-	PF00394;PF07731;PF07732	AA1
NEMBOFW57_001942	CDS	scaffold_1	6377852	6379900	-	PF00394;PF07731;PF07732	AA1
NEMBOFW57_003715	CDS	scaffold_1	11985498	11987532	-	PF00394;PF07731;PF07732	AA1
NEMBOFW57_004889	CDS	scaffold_2	393737	395891	-	PF00394;PF07731;PF07732	AA1
NEMBOFW57_007295	CDS	scaffold_3	2720324	2722605	+	PF00394;PF07731;PF07732	AA1
NEMBOFW57_007306	CDS	scaffold_3	2747326	2748712	+	PF00394;PF07731	AA1
NEMBOFW57_008766	CDS	scaffold_4	2707514	2709558	+	PF00394;PF07731	AA1
NEMBOFW57_009400	CDS	scaffold_5	592261	594499	+	PF00394;PF07731;PF07732	AA1
NEMBOFW57_009495	CDS	scaffold_5	957248	957986	-	PF07731	AA1
NEMBOFW57_009496	CDS	scaffold_5	958036	958769	-	PF00394	AA1
NEMBOFW57_009716	CDS	scaffold_5	1776867	1778604	-	PF00394;PF07732	AA1
NEMBOFW57_010300	CDS	scaffold_6	28563	30438	+	PF00394;PF07731;PF07732	AA1
NEMBOFW57_010375	CDS	scaffold_6	263202	265459	+	PF00394;PF07731;PF07732	AA1
NEMBOFW57_010159	CDS	scaffold_5	3199178	3201839	-	PF00394;PF07731;PF07732	AA1
NEMBOFW57_002858	CDS	scaffold_1	9314325	9315798	+		AA11
NEMBOFW57_004495	CDS	scaffold_1	14480791	14482071	-		AA11
NEMBOFW57_005322	CDS	scaffold_2	1811768	1812643	-		AA11
NEMBOFW57_005629	CDS	scaffold_2	2744099	2744584	-		AA11
NEMBOFW57_008081	CDS	scaffold_4	370388	371606	-		AA11
NEMBOFW57_003955	CDS	scaffold_1	12726044	12727482	-		AA12
NEMBOFW57_004822	CDS	scaffold_2	148978	151092	-		AA12
NEMBOFW57_008045	CDS	scaffold_4	268509	270107	-		AA12
NEMBOFW57_008990	CDS	scaffold_4	3430589	3434479	+		AA12
NEMBOFW57_004768	CDS	scaffold_1	15354700	15355921	+	PF00686	AA13+CBM20
NEMBOFW57_006204	CDS	scaffold_2	4559468	4560515	-	PF03067	AA16
NEMBOFW57_010266	CDS	scaffold_5	3556462	3557171	-	PF03067	AA16
NEMBOFW57_003132	CDS	scaffold_1	10143860	10145048	-	PF00141	AA2
NEMBOFW57_005252	CDS	scaffold_2	1589809	1593316	-	PF00141;PF01822	AA2
NEMBOFW57_008911	CDS	scaffold_4	3145709	3146895	+	PF00141	AA2
NEMBOFW57_001294	CDS	scaffold_1	4256726	4258534	+	PF00732;PF05199	AA3
NEMBOFW57_001352	CDS	scaffold_1	4423898	4425823	+	PF00732;PF05199;PF13450	AA3
NEMBOFW57_001476	CDS	scaffold_1	4828317	4831023	+	PF00732;PF05199	AA3
NEMBOFW57_001985	CDS	scaffold_1	6498221	6500119	+	PF00732;PF05199	AA3
NEMBOFW57_002775	CDS	scaffold_1	9029272	9030179	+	PF05199	AA3
NEMBOFW57_004137	CDS	scaffold_1	13305645	13308101	-	PF00732;PF05199	AA3
NEMBOFW57_004634	CDS	scaffold_1	14923606	14925702	+	PF00732;PF05199;PF13450	AA3
NEMBOFW57_005468	CDS	scaffold_2	2265499	2270012	-	PF00732	AA3
NEMBOFW57_006440	CDS	scaffold_3	97151	99114	-	PF00732;PF05199	AA3

NEMBOFW57_006627	CDS	scaffold_3	704274	709570	+	PF00732;PF05199;PF07690	AA3
NEMBOFW57_006905	CDS	scaffold_3	1539754	1541646	-	PF00732;PF01266;PF05199	AA3
NEMBOFW57_007166	CDS	scaffold_3	2268017	2270273	+	PF00732;PF05199	AA3
NEMBOFW57_007796	CDS	scaffold_3	4373557	4375492	+	PF00732;PF05199	AA3
NEMBOFW57_007947	CDS	scaffold_3	4854102	4856321	+	PF05199	AA3
NEMBOFW57_008008	CDS	scaffold_4	169532	174152	-	PF00732;PF01266;PF05199;PF13826	AA3
NEMBOFW57_008032	CDS	scaffold_4	231872	233542	+	PF00732;PF05199	AA3
NEMBOFW57_008083	CDS	scaffold_4	374702	376802	+	PF00732;PF05199	AA3
NEMBOFW57_009225	CDS	scaffold_5	36529	37545	-	PF00732	AA3
NEMBOFW57_010289	CDS	scaffold_5	3641192	3643227	+	PF00732;PF05199	AA3
NEMBOFW57_010319	CDS	scaffold_6	87482	89390	-	PF00732;PF05199	AA3
NEMBOFW57_010418	CDS	scaffold_6	386983	388870	-	PF00732;PF05199	AA3
NEMBOFW57_010449	CDS	scaffold_6	471545	473900	+	PF00732;PF05199	AA3
NEMBOFW57_010602	CDS	scaffold_6	997358	999198	+	PF00732;PF05199	AA3
NEMBOFW57_010731	CDS	scaffold_6	1454897	1456942	-	PF00732;PF05199	AA3
NEMBOFW57_010787	CDS	scaffold_6	1623052	1625026	+	PF00732;PF05199;PF13450	AA3
NEMBOFW57_010852	CDS	scaffold_6	1800907	1802864	+	PF00732;PF05199	AA3
NEMBOFW57_008624	CDS	scaffold_4	2230147	2232993	+	PF00732;PF05199;PF13450;PF16010	AA3+AA8
NEMBOFW57_010246	CDS	scaffold_5	3480001	3482222	-	PF00732;PF05199;PF13450	AA3+AA8
NEMBOFW57_001011	CDS	scaffold_1	3325230	3328077	+	PF00732;PF00734;PF05199;PF16010	AA3+AA8+CBM1
NEMBOFW57_001103	CDS	scaffold_1	3605180	3606927	-	PF01565	AA4
NEMBOFW57_001493	CDS	scaffold_1	4882953	4884755	+	PF01565;PF02913	AA4
NEMBOFW57_002033	CDS	scaffold_1	6641957	6643925	+	PF01565	AA4
NEMBOFW57_003515	CDS	scaffold_1	11427266	11429161	-	PF01565;PF02913	AA4
NEMBOFW57_003616	CDS	scaffold_1	11708907	11710856	-	PF01565	AA4
NEMBOFW57_007839	CDS	scaffold_3	4495333	4497434	-		AA4
NEMBOFW57_008217	CDS	scaffold_4	871088	872983	-	PF01565	AA4
NEMBOFW57_003104	CDS	scaffold_1	10065080	10068161	+	PF01822;PF07250	AA5
NEMBOFW57_010603	CDS	scaffold_6	1005519	1009001	-	PF01822;PF07250;PF09118	AA5
NEMBOFW57_002928	CDS	scaffold_1	9501001	9501724	+	PF03358	AA6
NEMBOFW57_001116	CDS	scaffold_1	3647293	3649346	-	PF01565;PF08031	AA7
NEMBOFW57_001310	CDS	scaffold_1	4300416	4302103	+	PF01565;PF08031	AA7
NEMBOFW57_001324	CDS	scaffold_1	4349179	4350644	-	PF01565;PF08031	AA7
NEMBOFW57_001457	CDS	scaffold_1	4776731	4778515	+	PF01565	AA7
NEMBOFW57_001464	CDS	scaffold_1	4795497	4797438	+	PF01565;PF08031	AA7
NEMBOFW57_004115	CDS	scaffold_1	13239286	13240896	-	PF01565;PF08031	AA7
NEMBOFW57_005776	CDS	scaffold_2	3158997	3160569	+	PF01565;PF08031	AA7
NEMBOFW57_008261	CDS	scaffold_4	982096	983979	-	PF01565;PF08031	AA7
NEMBOFW57_008561	CDS	scaffold_4	1996508	1998011	-	PF01565	AA7
NEMBOFW57_009290	CDS	scaffold_5	242623	244021	-	PF01565;PF02913	AA7
NEMBOFW57_008720	CDS	scaffold_4	2550426	2551208	-	PF16010	AA8
NEMBOFW57_001100	CDS	scaffold_1	3593528	3594461	+	PF03443	AA9
NEMBOFW57_001172	CDS	scaffold_1	3820127	3821078	-	PF03443	AA9
NEMBOFW57_001232	CDS	scaffold_1	3994178	3995188	+	PF03443	AA9
NEMBOFW57_001968	CDS	scaffold_1	6448752	6449524	-	PF03443	AA9

NEMBOFW57_002035	CDS	scaffold_1	6649002	6650263	-	PF00734;PF03443	AA9
NEMBOFW57_003567	CDS	scaffold_1	11576226	11577198	-	PF03443	AA9
NEMBOFW57_003664	CDS	scaffold_1	11838690	11839558	-	PF03443	AA9
NEMBOFW57_004004	CDS	scaffold_1	12851434	12852224	+	PF03443	AA9
NEMBOFW57_004061	CDS	scaffold_1	13078730	13079402	-		AA9
NEMBOFW57_004255	CDS	scaffold_1	13624235	13625042	+	PF03443	AA9
NEMBOFW57_004556	CDS	scaffold_1	14717941	14718767	-	PF03443	AA9
NEMBOFW57_005018	CDS	scaffold_2	823965	824816	-	PF03443	AA9
NEMBOFW57_005501	CDS	scaffold_2	2366543	2367434	+		AA9
NEMBOFW57_005556	CDS	scaffold_2	2526026	2526749	-	PF03443	AA9
NEMBOFW57_005701	CDS	scaffold_2	2952345	2953124	-	PF03443	AA9
NEMBOFW57_006551	CDS	scaffold_3	445195	447741	-	PF03443	AA9
NEMBOFW57_006769	CDS	scaffold_3	1144741	1145757	-	PF03443	AA9
NEMBOFW57_007215	CDS	scaffold_3	2476786	2477709	-	PF03443	AA9
NEMBOFW57_007753	CDS	scaffold_3	4228395	4229906	-	PF03443	AA9
NEMBOFW57_007876	CDS	scaffold_3	4629093	4630004	-	PF03443	AA9
NEMBOFW57_007901	CDS	scaffold_3	4707939	4708763	+	PF03443	AA9
NEMBOFW57_008051	CDS	scaffold_4	285372	286814	-	PF03443	AA9
NEMBOFW57_008059	CDS	scaffold_4	310415	311513	+	PF03443	AA9
NEMBOFW57_008519	CDS	scaffold_4	1850151	1851201	-	PF03443	AA9
NEMBOFW57_008623	CDS	scaffold_4	2226483	2227392	+	PF03443	AA9
NEMBOFW57_009147	CDS	scaffold_4	3888893	3889906	+	PF03443	AA9
NEMBOFW57_009366	CDS	scaffold_5	473538	474237	+	PF03443	AA9
NEMBOFW57_009636	CDS	scaffold_5	1524034	1524728	-	PF03443	AA9
NEMBOFW57_009689	CDS	scaffold_5	1668389	1669165	-	PF03443	AA9
NEMBOFW57_009842	CDS	scaffold_5	2201120	2204259	+	PF03443	AA9
NEMBOFW57_010244	CDS	scaffold_5	3476157	3476796	-	PF03443	AA9
NEMBOFW57_010245	CDS	scaffold_5	3477751	3478492	-	PF03443	AA9
NEMBOFW57_010597	CDS	scaffold_6	980301	981135	-	PF03443	AA9
NEMBOFW57_001044	CDS	scaffold_1	3442832	3443936	+	PF00734;PF03443	AA9+CBM1
NEMBOFW57_001466	CDS	scaffold_1	4805681	4806874	+	PF00734;PF03443	AA9+CBM1
NEMBOFW57_003857	CDS	scaffold_1	12408865	12409918	-	PF00734;PF03443	AA9+CBM1
NEMBOFW57_005429	CDS	scaffold_2	2144457	2145507	+	PF00734;PF03443	AA9+CBM1
NEMBOFW57_005558	CDS	scaffold_2	2530674	2531751	+	PF00734;PF03443	AA9+CBM1
NEMBOFW57_003584	CDS	scaffold_1	11623311	11624399	+	PF00734	CBM1
NEMBOFW57_010339	CDS	scaffold_6	148403	149267	+	PF09362	CBM1
NEMBOFW57_006352	CDS	scaffold_2	5156764	5158375	-		CBM18
NEMBOFW57_008264	CDS	scaffold_4	994118	995511	-	PF00704	CBM18+GH18
NEMBOFW57_009681	CDS	scaffold_5	1648888	1652886	+	PF00187;PF00704	CBM18+GH18
NEMBOFW57_001277	CDS	scaffold_1	4206641	4208843	+	PF03370;PF16760	CBM21
NEMBOFW57_005865	CDS	scaffold_2	3508992	3510561	-	PF04739;PF16561	CBM48
NEMBOFW57_001242	CDS	scaffold_1	4020587	4022648	+	PF01476	CBM50
NEMBOFW57_001286	CDS	scaffold_1	4229634	4231968	-	PF01476	CBM50
NEMBOFW57_006922	CDS	scaffold_3	1586660	1588077	-	PF01476	CBM50
NEMBOFW57_009213	CDS	scaffold_4	4078701	4080994	+	PF01476	CBM50

NEMBOFW57_009680	CDS	scaffold_5	1645946	1648042	-	PF01476	CBM50
NEMBOFW57_000190	CDS	scaffold_1	611214	615333	-	PF01544;PF10645	CBM52
NEMBOFW57_001829	CDS	scaffold_1	5998573	6000220	+	PF13472	CE0
NEMBOFW57_000278	CDS	scaffold_1	909735	910688	-	PF00756	CE1
NEMBOFW57_002054	CDS	scaffold_1	6711405	6712203	-	PF00326;PF10503	CE1
NEMBOFW57_004513	CDS	scaffold_1	14569225	14570309	-	PF00326;PF10503	CE1
NEMBOFW57_007800	CDS	scaffold_3	4382832	4383875	-	PF00326;PF00756;PF10503	CE1
NEMBOFW57_009348	CDS	scaffold_5	426542	427411	+		CE1
NEMBOFW57_009711	CDS	scaffold_5	1765223	1766352	+	PF10503	CE1
NEMBOFW57_001166	CDS	scaffold_1	3798469	3799429	+	PF00326;PF10503	CE1+CBM1
NEMBOFW57_002052	CDS	scaffold_1	6702774	6703967	-	PF00326;PF00734;PF10503	CE1+CBM1
NEMBOFW57_002906	CDS	scaffold_1	9439750	9440875	+	PF00326;PF00734;PF10503	CE1+CBM1
NEMBOFW57_004294	CDS	scaffold_1	13753922	13755078	+	PF00734	CE1+CBM1
NEMBOFW57_009603	CDS	scaffold_5	1408113	1409459	+	PF00326;PF00734	CE1+CBM1
NEMBOFW57_000887	CDS	scaffold_1	2916539	2918670	-	PF00135;PF07859	CE10
NEMBOFW57_001299	CDS	scaffold_1	4267806	4269891	-	PF00135	CE10
NEMBOFW57_002197	CDS	scaffold_1	7174648	7176659	+	PF00135	CE10
NEMBOFW57_002792	CDS	scaffold_1	9081793	9085836	+	PF00135;PF07859	CE10
NEMBOFW57_004037	CDS	scaffold_1	12975859	12977646	-	PF00135	CE10
NEMBOFW57_006420	CDS	scaffold_3	37334	40264	-	PF00004;PF07859	CE10
NEMBOFW57_006705	CDS	scaffold_3	961359	962794	-	PF07859	CE10
NEMBOFW57_007816	CDS	scaffold_3	4433165	4434415	+	PF07859;PF12697	CE10
NEMBOFW57_009275	CDS	scaffold_5	197386	199170	+	PF00135	CE10
NEMBOFW57_010736	CDS	scaffold_6	1472630	1473643	-	PF07859	CE10
NEMBOFW57_000286	CDS	scaffold_1	935243	937617	+	PF13472	CE12
NEMBOFW57_001439	CDS	scaffold_1	4727468	4728322	+	PF13472	CE12
NEMBOFW57_003741	CDS	scaffold_1	12060865	12062273	-		CE15
NEMBOFW57_004062	CDS	scaffold_1	13081274	13082468	+		CE15
NEMBOFW57_004373	CDS	scaffold_1	14067310	14068507	-		CE15
NEMBOFW57_004792	CDS	scaffold_2	59466	61756	+		CE15
NEMBOFW57_006599	CDS	scaffold_3	596950	598045	+		CE16
NEMBOFW57_009601	CDS	scaffold_5	1394849	1396161	+	PF00734	CE16+CBM1
NEMBOFW57_007038	CDS	scaffold_3	1900454	1901537	+	PF13472;PF17996	CE2
NEMBOFW57_007004	CDS	scaffold_3	1810201	1811063	-	PF13472	CE3
NEMBOFW57_008260	CDS	scaffold_4	981321	981941	+	PF13472	CE3
NEMBOFW57_009085	CDS	scaffold_4	3699957	3700779	+	PF00657;PF13472	CE3
NEMBOFW57_009569	CDS	scaffold_5	1292031	1293894	+		CE3
NEMBOFW57_009627	CDS	scaffold_5	1483597	1484334	+	PF13472	CE3
NEMBOFW57_009637	CDS	scaffold_5	1526105	1526619	+	PF13472	CE3
NEMBOFW57_002147	CDS	scaffold_1	7038857	7039610	-	PF01522	CE4
NEMBOFW57_004181	CDS	scaffold_1	13443416	13444265	+	PF01522;PF10096	CE4
NEMBOFW57_005718	CDS	scaffold_2	3006557	3007425	-	PF01522	CE4
NEMBOFW57_006469	CDS	scaffold_3	173778	176229	-	PF01522	CE4
NEMBOFW57_009329	CDS	scaffold_5	368298	369367	+	PF01522	CE4
NEMBOFW57_009578	CDS	scaffold_5	1336044	1336859	+	PF01522	CE4

NEMBOFW57_000180	CDS	scaffold_1	568814	569594	-	PF01522	CE4+CBM18
NEMBOFW57_000575	CDS	scaffold_1	1888377	1889863	-		CE4+CBM18
NEMBOFW57_000461	CDS	scaffold_1	1517382	1518390	-	PF01083	CE5
NEMBOFW57_004684	CDS	scaffold_1	15083321	15084132	-	PF01083	CE5
NEMBOFW57_005577	CDS	scaffold_2	2591485	2592367	-	PF01083	CE5
NEMBOFW57_006873	CDS	scaffold_3	1451147	1451982	+	PF01083	CE5
NEMBOFW57_006941	CDS	scaffold_3	1636360	1637083	-	PF01083	CE5
NEMBOFW57_007936	CDS	scaffold_3	4821592	4822332	-	PF01083	CE5
NEMBOFW57_008131	CDS	scaffold_4	511089	511847	-	PF01083	CE5
NEMBOFW57_010955	CDS	scaffold_6	2173618	2174694	+	PF00734;PF01083	CE5
NEMBOFW57_006728	CDS	scaffold_3	1033919	1034924	-	PF01095	CE8
NEMBOFW57_008978	CDS	scaffold_4	3381436	3382732	-		CE9
NEMBOFW57_000322	CDS	scaffold_1	1041990	1043513	-	PF00232	GH1
NEMBOFW57_004075	CDS	scaffold_1	13115290	13116875	-	PF00232	GH1
NEMBOFW57_001316	CDS	scaffold_1	4322419	4323664	-	PF00331	GH10
NEMBOFW57_004303	CDS	scaffold_1	13781886	13783049	-	PF00331	GH10
NEMBOFW57_005777	CDS	scaffold_2	3161034	3162451	-	PF00331	GH10
NEMBOFW57_006022	CDS	scaffold_2	4008027	4009125	-	PF00331	GH10
NEMBOFW57_007190	CDS	scaffold_3	2411581	2414207	-	PF00121;PF00331	GH10
NEMBOFW57_007979	CDS	scaffold_4	77540	78571	+		GH10
NEMBOFW57_008581	CDS	scaffold_4	2081765	2082875	-	PF00331	GH10
NEMBOFW57_008598	CDS	scaffold_4	2151442	2152705	+	PF00331	GH10
NEMBOFW57_009365	CDS	scaffold_5	471103	473068	+	PF00331	GH10
NEMBOFW57_010962	CDS	scaffold_6	2194209	2195606	+	PF00331;PF00734	GH10
NEMBOFW57_009546	CDS	scaffold_5	1192186	1193463	-	PF00331;PF00734	GH10+CBM1
NEMBOFW57_003635	CDS	scaffold_1	11763810	11765170	-	PF00331;PF00734	GH10+CBM1
NEMBOFW57_003655	CDS	scaffold_1	11816729	11818084	+	PF00331	GH10+CBM1
NEMBOFW57_006732	CDS	scaffold_3	1044277	1045621	+	PF00331;PF00734	GH10+CBM1
NEMBOFW57_010166	CDS	scaffold_5	3219944	3221266	+	PF00734	GH10+CBM1
NEMBOFW57_008995	CDS	scaffold_4	3444186	3445323	+	PF07470	GH105
NEMBOFW57_003719	CDS	scaffold_1	11997682	11998401	-	PF00457	GH11
NEMBOFW57_004737	CDS	scaffold_1	15250602	15251375	-	PF00457	GH11
NEMBOFW57_006283	CDS	scaffold_2	4960715	4961455	+	PF00457	GH11
NEMBOFW57_008070	CDS	scaffold_4	341398	342163	-	PF00457	GH11
NEMBOFW57_008340	CDS	scaffold_4	1230371	1231121	-	PF00457	GH11
NEMBOFW57_010837	CDS	scaffold_6	1755679	1756503	-	PF00457	GH11
NEMBOFW57_010850	CDS	scaffold_6	1797080	1797806	+	PF00457	GH11
NEMBOFW57_010934	CDS	scaffold_6	2105284	2106029	-	PF00457	GH11
NEMBOFW57_001362	CDS	scaffold_1	4452303	4453929	-	PF00457;PF00734	GH11+CBM1
NEMBOFW57_006351	CDS	scaffold_2	5151552	5152552	-	PF00457;PF00734	GH11+CBM1
NEMBOFW57_004027	CDS	scaffold_1	12907555	12909618	+	PF03537	GH114
NEMBOFW57_004078	CDS	scaffold_1	13123869	13127504	+	PF15979;PF17829	GH115
NEMBOFW57_005380	CDS	scaffold_2	2005498	2008573	+	PF15979;PF17829	GH115
NEMBOFW57_009324	CDS	scaffold_5	352897	355210	+	PF15979;PF17829	GH115
NEMBOFW57_001184	CDS	scaffold_1	3849206	3850065	+	PF01670	GH12

NEMBOFW57_007690	CDS	scaffold_3	4040105	4043148	-	PF01670	GH12+CE1
NEMBOFW57_001084	CDS	scaffold_1	3545086	3548913	+	PF00535;PF01670;PF13506;PF13632;PF13641	GH12+GT2
NEMBOFW57_003187	CDS	scaffold_1	10323927	10326153	+	PF06824	GH125
NEMBOFW57_007314	CDS	scaffold_3	2766312	2768147	-	PF06824	GH125
NEMBOFW57_009130	CDS	scaffold_4	3831316	3832961	+	PF06824	GH125
NEMBOFW57_004390	CDS	scaffold_1	14118483	14119767	-	PF11790	GH128
NEMBOFW57_006415	CDS	scaffold_3	30156	31221	+	PF11790	GH128
NEMBOFW57_007943	CDS	scaffold_3	4841297	4842346	+	PF11790	GH128
NEMBOFW57_000015	CDS	scaffold_1	52852	54618	-	PF00128	GH13
NEMBOFW57_000224	CDS	scaffold_1	730493	732761	-	PF00128	GH13
NEMBOFW57_001121	CDS	scaffold_1	3663083	3667180	-	PF01965	GH13
NEMBOFW57_002821	CDS	scaffold_1	9184595	9186608	-	PF00128	GH13
NEMBOFW57_008067	CDS	scaffold_4	328464	330417	+	PF00128	GH13
NEMBOFW57_009282	CDS	scaffold_5	221952	223685	+	PF00128;PF09260	GH13
NEMBOFW57_009402	CDS	scaffold_5	599810	601557	-	PF00128	GH13
NEMBOFW57_009412	CDS	scaffold_5	635893	637267	+	PF00128;PF02806	GH13
NEMBOFW57_002380	CDS	scaffold_1	7774384	7777132	+	PF00128;PF00686;PF09260	GH13+CBM20
NEMBOFW57_009709	CDS	scaffold_5	1758980	1761502	+	PF02806;PF02922	GH13+CBM48
NEMBOFW57_002779	CDS	scaffold_1	9040088	9044947	-	PF06202;PF14699;PF14701;PF14702	GH13+GH133
NEMBOFW57_004220	CDS	scaffold_1	13532066	13537776	-	PF00534;PF08323	GH13+GT5
NEMBOFW57_004221	CDS	scaffold_1	13537870	13539312	-	PF00128	GH13+GT5
NEMBOFW57_009281	CDS	scaffold_5	211736	219129	-	PF00128;PF00534;PF08323	GH13+GT5
NEMBOFW57_002736	CDS	scaffold_1	8918484	8919420	-	PF18271	GH131
NEMBOFW57_010725	CDS	scaffold_6	1442514	1443482	+	PF18271	GH131
NEMBOFW57_004659	CDS	scaffold_1	15009797	15010987	+	PF00734	GH131+CBM1
NEMBOFW57_000683	CDS	scaffold_1	2240755	2241607	-	PF18271	GH131+CMB1
NEMBOFW57_002244	CDS	scaffold_1	7296940	7298266	-	PF03856	GH132
NEMBOFW57_007260	CDS	scaffold_3	2612920	2614418	+	PF03856	GH132
NEMBOFW57_001504	CDS	scaffold_1	4910832	4911541	-		GH134
NEMBOFW57_005745	CDS	scaffold_2	3076608	3077537	+	PF12138	GH135
NEMBOFW57_003754	CDS	scaffold_1	12089760	12091265	-	PF15892	GH145
NEMBOFW57_001379	CDS	scaffold_1	4511352	4513230	-	PF07944	GH146+CBM13
NEMBOFW57_002472	CDS	scaffold_1	8081026	8083315	-	PF00723	GH15
NEMBOFW57_004103	CDS	scaffold_1	13201102	13203729	-	PF00686;PF00723	GH15+CBM20
NEMBOFW57_007659	CDS	scaffold_3	3952003	3954069	-	PF00686;PF00723	GH15+CBM20
NEMBOFW57_009932	CDS	scaffold_5	2486801	2488983	-	PF00686;PF00723	GH15+CBM20
NEMBOFW57_001921	CDS	scaffold_1	6313488	6315008	+	PF00314	GH152
NEMBOFW57_005402	CDS	scaffold_2	2076096	2078912	+	PF10022	GH154
NEMBOFW57_000456	CDS	scaffold_1	1496553	1497977	+	PF00722	GH16
NEMBOFW57_000956	CDS	scaffold_1	3167036	3168607	+	PF00722	GH16
NEMBOFW57_001029	CDS	scaffold_1	3392678	3393566	-	PF00722	GH16
NEMBOFW57_001400	CDS	scaffold_1	4595500	4597330	-	PF00722	GH16
NEMBOFW57_001416	CDS	scaffold_1	4654802	4656315	-	PF00722	GH16
NEMBOFW57_002428	CDS	scaffold_1	7938533	7939628	+		GH16
NEMBOFW57_003172	CDS	scaffold_1	10266578	10267720	+	PF00722	GH16

NEMBOFW57_004987	CDS	scaffold_2	718428	720942	-		GH16
NEMBOFW57_005900	CDS	scaffold_2	3600232	3601697	+	PF00722	GH16
NEMBOFW57_006737	CDS	scaffold_3	1056613	1057620	+	PF00722	GH16
NEMBOFW57_006999	CDS	scaffold_3	1798259	1799205	+	PF00722	GH16
NEMBOFW57_007994	CDS	scaffold_4	133760	134983	-	PF00722;PF00734	GH16
NEMBOFW57_008500	CDS	scaffold_4	1774843	1776027	-	PF00722	GH16
NEMBOFW57_009121	CDS	scaffold_4	3807592	3808568	+	PF00722	GH16
NEMBOFW57_000084	CDS	scaffold_1	275162	276646	-	PF00722	GH16+CBM18
NEMBOFW57_000164	CDS	scaffold_1	508845	510042	-		GH17
NEMBOFW57_002019	CDS	scaffold_1	6599223	6601419	-		GH17
NEMBOFW57_002419	CDS	scaffold_1	7902040	7903669	-		GH17
NEMBOFW57_007588	CDS	scaffold_3	3718885	3719933	+		GH17
NEMBOFW57_002998	CDS	scaffold_1	9695146	9696325	+	PF00704	GH18
NEMBOFW57_003268	CDS	scaffold_1	10603067	10604302	+	PF00704	GH18
NEMBOFW57_004186	CDS	scaffold_1	13451613	13452917	+	PF00704	GH18
NEMBOFW57_005276	CDS	scaffold_2	1661182	1662247	-	PF00704	GH18
NEMBOFW57_007866	CDS	scaffold_3	4596706	4597998	+	PF00704	GH18
NEMBOFW57_009632	CDS	scaffold_5	1502890	1504071	+	PF00704	GH18
NEMBOFW57_010448	CDS	scaffold_6	469221	470505	+	PF00704	GH18
NEMBOFW57_006970	CDS	scaffold_3	1709828	1711219	-		GH18+CBM1
NEMBOFW57_000686	CDS	scaffold_1	2249463	2251211	-	PF00704;PF01476	GH18+CBM18
NEMBOFW57_001239	CDS	scaffold_1	4013087	4014641	-	PF00187;PF00704	GH18+CBM18
NEMBOFW57_001287	CDS	scaffold_1	4233026	4236857	+	PF00187;PF00704	GH18+CBM18
NEMBOFW57_003872	CDS	scaffold_1	12456276	12460861	-	PF00704	GH18+CBM18
NEMBOFW57_008621	CDS	scaffold_4	2215576	2219221	-	PF00187;PF00704	GH18+CBM18
NEMBOFW57_009212	CDS	scaffold_4	4075457	4076758	-	PF00704	GH18+CBM18
NEMBOFW57_000124	CDS	scaffold_1	396078	397378	+		GH18+CBM18+CBM50
NEMBOFW57_002946	CDS	scaffold_1	9541889	9543616	+	PF01476	GH18+CBM18+CBM50
NEMBOFW57_010628	CDS	scaffold_6	1115010	1120171	+	PF00704;PF01476	GH18+CBM24
NEMBOFW57_001690	CDS	scaffold_1	5526235	5529144	+	PF01476	GH18+CBM50
NEMBOFW57_009563	CDS	scaffold_5	1267721	1272992	-	PF00704;PF01476	GH18+CBM50+CBM18
NEMBOFW57_004117	CDS	scaffold_1	13250646	13254825	-	PF00704;PF01476	GH18+CBM50+CBM18
NEMBOFW57_006926	CDS	scaffold_3	1595170	1599488	+	PF00704;PF01476	GH18+CBM50+CBM18
NEMBOFW57_003549	CDS	scaffold_1	11513994	11516599	-	PF00703;PF17753;PF17786	GH2
NEMBOFW57_004374	CDS	scaffold_1	14069371	14072139	+	PF00703;PF02837;PF16355;PF18565	GH2
NEMBOFW57_008526	CDS	scaffold_4	1870141	1873562	-	PF00703;PF02836;PF02837;PF02929;PF16353	GH2
NEMBOFW57_008742	CDS	scaffold_4	2626316	2628926	-	PF00703;PF17786	GH2
NEMBOFW57_009152	CDS	scaffold_4	3898863	3901470	-	PF00703;PF02836;PF02837;PF16355;PF18565	GH2
NEMBOFW57_010681	CDS	scaffold_6	1319302	1323787	-	PF00703;PF18368	GH2
NEMBOFW57_002850	CDS	scaffold_1	9286610	9289919	+	PF00703;PF02836;PF02837;PF02929	GH2+CBM32+CBM51+CBM67
NEMBOFW57_002037	CDS	scaffold_1	6654956	6656701	-	PF02837	GH2+CBM42+CBM67
NEMBOFW57_003169	CDS	scaffold_1	10252597	10254535	+	PF00728;PF14845	GH20
NEMBOFW57_004532	CDS	scaffold_1	14648586	14649497	+	PF00959	GH24
NEMBOFW57_010763	CDS	scaffold_6	1544629	1545580	+	PF00959	GH24
NEMBOFW57_002920	CDS	scaffold_1	9472722	9474414	-	PF02156;PF16990	GH26+CBM35

NEMBOFW57_003773	CDS	scaffold_1	12147775	12149300	+	PF17801	GH27
NEMBOFW57_008762	CDS	scaffold_4	2698404	2699868	+	PF16499;PF17801	GH27
NEMBOFW57_004007	CDS	scaffold_1	12857839	12859391	-	PF00295	GH28
NEMBOFW57_005935	CDS	scaffold_2	3698268	3699956	+	PF00295	GH28
NEMBOFW57_008244	CDS	scaffold_4	940732	941937	+	PF00295	GH28
NEMBOFW57_010726	CDS	scaffold_6	1445426	1447379	+	PF00295;PF00734	GH28
NEMBOFW57_002874	CDS	scaffold_1	9357149	9359108	+	PF00933;PF01915;PF14310	GH3
NEMBOFW57_002908	CDS	scaffold_1	9443270	9444149	-	PF01915	GH3
NEMBOFW57_002909	CDS	scaffold_1	9444181	9447176	-	PF00933	GH3
NEMBOFW57_003609	CDS	scaffold_1	11692437	11695180	+	PF00933;PF01915;PF07691;PF14310	GH3
NEMBOFW57_005247	CDS	scaffold_2	1562068	1565876	-	PF00933;PF01915;PF14310	GH3
NEMBOFW57_006021	CDS	scaffold_2	4005596	4007873	+	PF00933;PF01915;PF14310	GH3
NEMBOFW57_007007	CDS	scaffold_3	1814652	1816847	-	PF01915;PF07691;PF14310	GH3
NEMBOFW57_007880	CDS	scaffold_3	4640311	4642651	-	PF00933;PF01915;PF14310	GH3
NEMBOFW57_007883	CDS	scaffold_3	4651129	4653390	-	PF00933;PF01915;PF14310	GH3
NEMBOFW57_008022	CDS	scaffold_4	205780	208418	-	PF00933;PF01915;PF07691;PF14310	GH3
NEMBOFW57_008191	CDS	scaffold_4	810181	812857	-	PF00933;PF01915;PF14310	GH3
NEMBOFW57_008747	CDS	scaffold_4	2642375	2644583	-	PF00933;PF01915;PF14310	GH3
NEMBOFW57_008979	CDS	scaffold_4	3383991	3387271	+	PF00933	GH3
NEMBOFW57_009222	CDS	scaffold_5	24500	26891	-	PF00933;PF01915;PF14310	GH3
NEMBOFW57_001375	CDS	scaffold_1	4499519	4502456	+	PF00933;PF01915;PF14310	GH3+CBM1
NEMBOFW57_003505	CDS	scaffold_1	11395171	11397801	-	PF00933;PF01915;PF14310	GH3+CBM1
NEMBOFW57_003860	CDS	scaffold_1	12416632	12419469	-	PF01915;PF14310	GH3+CBM1
NEMBOFW57_002699	CDS	scaffold_1	8797928	8800971	+	PF00933;PF01915;PF07691;PF14310	GH3+CBM6
NEMBOFW57_001051	CDS	scaffold_1	3456006	3460539	+	PF00933;PF01915;PF14310	GH3+CMB1
NEMBOFW57_000554	CDS	scaffold_1	1818275	1822016	+	PF02057;PF02110;PF02581;PF14587	GH30
NEMBOFW57_007121	CDS	scaffold_3	2141255	2142658	+	PF02057;PF17189	GH30
NEMBOFW57_009537	CDS	scaffold_5	1160241	1161871	-	PF02055	GH30
NEMBOFW57_002965	CDS	scaffold_1	9597538	9604600	-	PF01055;PF16863	GH31
NEMBOFW57_004860	CDS	scaffold_2	281082	284132	+	PF01055;PF13802;PF16863	GH31
NEMBOFW57_005293	CDS	scaffold_2	1710160	1713182	+	PF01055;PF13802	GH31
NEMBOFW57_007820	CDS	scaffold_3	4440090	4442592	-	PF01055	GH31
NEMBOFW57_008838	CDS	scaffold_4	2912138	2914712	-	PF01055;PF13802	GH31
NEMBOFW57_009223	CDS	scaffold_5	27864	30280	+	PF01055;PF13802	GH31
NEMBOFW57_009691	CDS	scaffold_5	1675769	1685051	+	PF01055;PF13520;PF13802;PF16863	GH31
NEMBOFW57_006334	CDS	scaffold_2	5112140	5113644	+	PF08244	GH32
NEMBOFW57_004148	CDS	scaffold_1	13335183	13337235	-	PF00251;PF08244	GH32+CBM38
NEMBOFW57_004150	CDS	scaffold_1	13342119	13347183	-	PF00251;PF08244	GH32+CBM38
NEMBOFW57_001288	CDS	scaffold_1	4237299	4239254	-		GH35
NEMBOFW57_001853	CDS	scaffold_1	6068884	6075121	+	PF01979;PF10435;PF13363;PF13364	GH35
NEMBOFW57_005693	CDS	scaffold_2	2920266	2924017	-	PF01301;PF10435;PF13363;PF13364	GH35
NEMBOFW57_002951	CDS	scaffold_1	9551147	9552897	-		GH36
NEMBOFW57_003451	CDS	scaffold_1	11191634	11193902	+	PF01204	GH37
NEMBOFW57_006027	CDS	scaffold_2	4015729	4022291	-	PF01204;PF07492;PF11885	GH37
NEMBOFW57_002602	CDS	scaffold_1	8502111	8505408	+	PF01074;PF07748;PF09261;PF17677	GH38

NEMBOFW57_000134	CDS	scaffold_1	418231	419919	-	PF04616;PF17851	GH43
NEMBOFW57_001526	CDS	scaffold_1	4980103	4981087	+	PF04616	GH43
NEMBOFW57_003627	CDS	scaffold_1	11738435	11739392	+	PF04616	GH43
NEMBOFW57_003714	CDS	scaffold_1	11981106	11982534	-	PF04616;PF17851	GH43
NEMBOFW57_005151	CDS	scaffold_2	1240541	1241706	+	PF04616	GH43
NEMBOFW57_007869	CDS	scaffold_3	4606538	4608124	-	PF04616	GH43
NEMBOFW57_007945	CDS	scaffold_3	4845094	4846708	+	PF04616;PF17851	GH43
NEMBOFW57_008294	CDS	scaffold_4	1076937	1078973	-	PF04616;PF17851	GH43
NEMBOFW57_008512	CDS	scaffold_4	1816089	1818665	+	PF04616	GH43
NEMBOFW57_008515	CDS	scaffold_4	1825601	1826863	+		GH43
NEMBOFW57_009241	CDS	scaffold_5	92164	93267	-	PF04616	GH43
NEMBOFW57_009841	CDS	scaffold_5	2198551	2200175	-	PF04616;PF17851	GH43
NEMBOFW57_010633	CDS	scaffold_6	1195780	1197519	+	PF04616;PF17851	GH43
NEMBOFW57_010634	CDS	scaffold_6	1197528	1199412	-	PF04616;PF17851	GH43
NEMBOFW57_004619	CDS	scaffold_1	14889834	14891428	+	PF04616	GH43+CBM35
NEMBOFW57_001268	CDS	scaffold_1	4158478	4159885	-	PF04616	GH43+CBM42
NEMBOFW57_010808	CDS	scaffold_6	1677397	1678739	+	PF04616	GH43+CBM6
NEMBOFW57_002500	CDS	scaffold_1	8166561	8167492	-	PF02015	GH45
NEMBOFW57_009019	CDS	scaffold_4	3519101	3520083	-	PF00734;PF02015	GH45+CBM1
NEMBOFW57_002507	CDS	scaffold_1	8186913	8188278	-	PF01532	GH47
NEMBOFW57_002700	CDS	scaffold_1	8801941	8803879	+	PF01532	GH47
NEMBOFW57_003122	CDS	scaffold_1	10115855	10118219	+	PF01532	GH47
NEMBOFW57_003279	CDS	scaffold_1	10643413	10646555	-	PF01532	GH47
NEMBOFW57_003808	CDS	scaffold_1	12266399	12269450	+	PF01532	GH47
NEMBOFW57_004824	CDS	scaffold_2	154742	156633	-	PF01532	GH47
NEMBOFW57_005714	CDS	scaffold_2	2997296	2999138	+	PF01532	GH47
NEMBOFW57_006190	CDS	scaffold_2	4512896	4516846	-	PF01532;PF10639	GH47
NEMBOFW57_008471	CDS	scaffold_4	1675103	1676654	-	PF01532	GH47
NEMBOFW57_004670	CDS	scaffold_1	15043511	15045158	+	PF03718;PF17433;PF18783;PF18841	GH49
NEMBOFW57_000239	CDS	scaffold_1	792528	793794	-	PF00150	GH5
NEMBOFW57_001193	CDS	scaffold_1	3881936	3883182	+	PF00150	GH5
NEMBOFW57_002806	CDS	scaffold_1	9119834	9121185	-	PF00150;PF00734	GH5
NEMBOFW57_003411	CDS	scaffold_1	11055544	11056792	-	PF00150	GH5
NEMBOFW57_003806	CDS	scaffold_1	12255605	12257958	+	PF18564	GH5
NEMBOFW57_004769	CDS	scaffold_1	15356749	15358088	+	PF00150;PF02836	GH5
NEMBOFW57_006899	CDS	scaffold_3	1521720	1522961	+	PF00150;PF02836	GH5
NEMBOFW57_008096	CDS	scaffold_4	416569	418114	-	PF00150	GH5
NEMBOFW57_008436	CDS	scaffold_4	1576626	1578904	+	PF00150	GH5
NEMBOFW57_009318	CDS	scaffold_5	338234	339586	+	PF00150	GH5
NEMBOFW57_009419	CDS	scaffold_5	651749	653033	+	PF00150	GH5
NEMBOFW57_005502	CDS	scaffold_2	2368415	2369636	+	PF00150	GH5
NEMBOFW57_003576	CDS	scaffold_1	11597533	11598806	+	PF00150;PF00734	GH5+CBM1
NEMBOFW57_004293	CDS	scaffold_1	13751650	13752902	+	PF00150	GH5+CBM1
NEMBOFW57_006677	CDS	scaffold_3	882697	884307	+	PF00150;PF00734	GH5+CBM1
NEMBOFW57_010904	CDS	scaffold_6	2018741	2020303	+	PF00150;PF00734;PF02836	GH5+CBM1

NEMBOFW57_001185	CDS	scaffold_1	3851001	3853459	+	PF06964	GH51
NEMBOFW57_009294	CDS	scaffold_5	253850	255598	+	PF06964	GH51
NEMBOFW57_002223	CDS	scaffold_1	7237958	7239061	+	PF07745	GH53
NEMBOFW57_000338	CDS	scaffold_1	1101226	1102774	+	PF05270;PF09206	GH54+CBM13+CBM42
NEMBOFW57_001114	CDS	scaffold_1	3637541	3639898	-	PF12708	GH55
NEMBOFW57_001411	CDS	scaffold_1	4628611	4631144	-	PF12708	GH55
NEMBOFW57_003605	CDS	scaffold_1	11682146	11684344	-		GH55
NEMBOFW57_005046	CDS	scaffold_2	923511	925688	+	PF05048;PF12708	GH55
NEMBOFW57_005605	CDS	scaffold_2	2672591	2675976	+	PF12708	GH55
NEMBOFW57_008041	CDS	scaffold_4	255307	258238	+	PF12708	GH55
NEMBOFW57_008270	CDS	scaffold_4	1008917	1011398	-		GH55
NEMBOFW57_008622	CDS	scaffold_4	2220392	2225981	+	PF12708	GH55
NEMBOFW57_008865	CDS	scaffold_4	3005592	3010005	+	PF12708	GH55
NEMBOFW57_009388	CDS	scaffold_5	548389	552120	+	PF12708	GH55
NEMBOFW57_009521	CDS	scaffold_5	1104155	1105771	+		GH55
NEMBOFW57_009538	CDS	scaffold_5	1165181	1167632	-	PF12708	GH55
NEMBOFW57_010770	CDS	scaffold_6	1570758	1573223	+	PF12708	GH55
NEMBOFW57_004785	CDS	scaffold_2	39065	40724	+	PF00734;PF01341	GH6
NEMBOFW57_006383	CDS	scaffold_2	5245138	5246341	+	PF01341	GH6
NEMBOFW57_008641	CDS	scaffold_4	2280206	2281531	+	PF01341	GH6
NEMBOFW57_009027	CDS	scaffold_4	3537837	3539282	-	PF00734;PF01341	GH6
NEMBOFW57_001269	CDS	scaffold_1	4160536	4161616	+	PF03664	GH62
NEMBOFW57_005761	CDS	scaffold_2	3126040	3127183	+	PF03664	GH62
NEMBOFW57_003626	CDS	scaffold_1	11735467	11736938	+	PF00734;PF03664	GH62+CBM1
NEMBOFW57_009999	CDS	scaffold_5	2673144	2674317	+	PF00734;PF03664	GH62+CBM1
NEMBOFW57_004071	CDS	scaffold_1	13103497	13106752	-	PF03664	GH62+CBM13
NEMBOFW57_005944	CDS	scaffold_2	3731173	3733805	+	PF03200;PF16923	GH63
NEMBOFW57_005656	CDS	scaffold_2	2811882	2813125	+	PF16483	GH64
NEMBOFW57_004236	CDS	scaffold_1	13573183	13575748	-	PF03648;PF07477;PF07488	GH67
NEMBOFW57_003080	CDS	scaffold_1	9982865	9984411	-	PF00840	GH7
NEMBOFW57_003557	CDS	scaffold_1	11542158	11546304	-	PF00106;PF00840;PF02566;PF08659;PF13561	GH7
NEMBOFW57_003636	CDS	scaffold_1	11766358	11767810	+	PF00734;PF00840	GH7
NEMBOFW57_008029	CDS	scaffold_4	221659	223194	+	PF00840	GH7
NEMBOFW57_008456	CDS	scaffold_4	1628555	1630147	+	PF00840	GH7
NEMBOFW57_009028	CDS	scaffold_4	3540688	3541556	+	PF00840	GH7
NEMBOFW57_009077	CDS	scaffold_4	3676976	3678312	-	PF00840	GH7
NEMBOFW57_010165	CDS	scaffold_5	3216684	3218081	-	PF00840	GH7
NEMBOFW57_010290	CDS	scaffold_5	3644934	3646112	+		GH7
NEMBOFW57_010291	CDS	scaffold_5	3646591	3647275	-		GH7
NEMBOFW57_007704	CDS	scaffold_3	4089091	4090788	-	PF00734;PF00840	GH7+CBM1
NEMBOFW57_002060	CDS	scaffold_1	6730441	6731795	-	PF03659	GH71
NEMBOFW57_005284	CDS	scaffold_2	1684353	1686035	+	PF02127	GH71
NEMBOFW57_009442	CDS	scaffold_5	721660	723074	+		GH71
NEMBOFW57_004092	CDS	scaffold_1	13176015	13179112	+	PF03659	GH71+CBM24
NEMBOFW57_009012	CDS	scaffold_4	3504887	3506886	+		GH71+CBM24

NEMBOFW57_001461	CDS	scaffold_1	4784488	4787358	+	PF00704;PF03659	GH71+CBM24+GH18
NEMBOFW57_004454	CDS	scaffold_1	14337274	14338894	+	PF03198	GH72
NEMBOFW57_005031	CDS	scaffold_2	879545	881083	+	PF00150;PF03198	GH72
NEMBOFW57_007083	CDS	scaffold_3	2036627	2038121	-	PF03198	GH72
NEMBOFW57_009421	CDS	scaffold_5	653607	657095	-	PF03198	GH72
NEMBOFW57_005755	CDS	scaffold_2	3112777	3114453	+	PF02836;PF03198;PF07983	GH72+CBM43
NEMBOFW57_008527	CDS	scaffold_4	1874767	1877497	+	PF00734	GH74+CBM1
NEMBOFW57_003728	CDS	scaffold_1	12026732	12027728	+	PF07335	GH75
NEMBOFW57_006928	CDS	scaffold_3	1602455	1603935	-	PF07335	GH75
NEMBOFW57_000477	CDS	scaffold_1	1576751	1577709	+	PF03663	GH76
NEMBOFW57_001637	CDS	scaffold_1	5332456	5336629	-	PF00067;PF03663	GH76
NEMBOFW57_003481	CDS	scaffold_1	11315701	11317419	-		GH76
NEMBOFW57_005915	CDS	scaffold_2	3646476	3647898	+	PF03663	GH76
NEMBOFW57_006089	CDS	scaffold_2	4231761	4233305	+	PF03663	GH76
NEMBOFW57_006511	CDS	scaffold_3	312963	314239	-	PF03663	GH76
NEMBOFW57_006597	CDS	scaffold_3	592003	593332	+	PF03663	GH76
NEMBOFW57_007041	CDS	scaffold_3	1910601	1912077	-	PF03663	GH76
NEMBOFW57_009270	CDS	scaffold_5	183235	184717	+	PF03663	GH76
NEMBOFW57_010365	CDS	scaffold_6	223922	225466	+	PF03663	GH76
NEMBOFW57_010567	CDS	scaffold_6	867075	868320	+	PF03663	GH76
NEMBOFW57_004271	CDS	scaffold_1	13691680	13693810	+	PF17389;PF17390	GH78
NEMBOFW57_004076	CDS	scaffold_1	13117487	13120262	+	PF05592;PF08531;PF17389;PF17390	GH78+CBM67
NEMBOFW57_007008	CDS	scaffold_3	1818067	1820907	+	PF05592;PF08531;PF17389;PF17390	GH78+CBM67
NEMBOFW57_008078	CDS	scaffold_4	362493	364995	+	PF17389	GH78+CBM67
NEMBOFW57_008230	CDS	scaffold_4	898913	901343	+	PF08531;PF17389	GH78+CBM67
NEMBOFW57_003457	CDS	scaffold_1	11214710	11216466	+	PF16862	GH79
NEMBOFW57_004002	CDS	scaffold_1	12846836	12848414	-	PF16862	GH79
NEMBOFW57_008077	CDS	scaffold_4	359467	361504	-	PF16862	GH79
NEMBOFW57_008132	CDS	scaffold_4	515294	516425	-	PF16862	GH79
NEMBOFW57_009514	CDS	scaffold_5	1045590	1047343	+	PF16862	GH79
NEMBOFW57_004738	CDS	scaffold_1	15253418	15258779	-	PF00135;PF07859;PF16862	GH79+CE1
NEMBOFW57_004668	CDS	scaffold_1	15031855	15035158	-	PF03639;PF17652	GH81
NEMBOFW57_008477	CDS	scaffold_4	1687272	1692503	+	PF03639;PF17652	GH81
NEMBOFW57_004772	CDS	scaffold_1	15364971	15367655	+	PF07470	GH88
NEMBOFW57_007118	CDS	scaffold_3	2130007	2132705	-	PF07971;PF17678	GH92
NEMBOFW57_008891	CDS	scaffold_4	3086670	3089526	-	PF07971;PF17678	GH92
NEMBOFW57_007618	CDS	scaffold_3	3812333	3813524	-		GH93
NEMBOFW57_007691	CDS	scaffold_3	4044171	4045417	+		GH93
NEMBOFW57_009267	CDS	scaffold_5	173616	174792	-		GH93
NEMBOFW57_009604	CDS	scaffold_5	1412889	1415263	-	PF06165;PF17167	GH94
NEMBOFW57_004555	CDS	scaffold_1	14714558	14717141	-	PF14498	GH95
NEMBOFW57_006721	CDS	scaffold_3	1016062	1017882	-		GH95
NEMBOFW57_000885	CDS	scaffold_1	2909125	2912797	+	PF03033	GT1
NEMBOFW57_002708	CDS	scaffold_1	8832565	8837048	-		GT1
NEMBOFW57_004744	CDS	scaffold_1	15278180	15279557	-		GT1

NEMBOFW57_007039	CDS	scaffold_3	1902832	1904190	+		GT1
NEMBOFW57_008656	CDS	scaffold_4	2328264	2330917	+	PF04101	GT1
NEMBOFW57_009579	CDS	scaffold_5	1337167	1338742	-	PF04101	GT1
NEMBOFW57_010057	CDS	scaffold_5	2843887	2844839	-	PF08660	GT1
NEMBOFW57_010391	CDS	scaffold_6	322864	325741	+	PF03033	GT1
NEMBOFW57_010643	CDS	scaffold_6	1228889	1233591	-	PF00169;PF02893;PF03033	GT1
NEMBOFW57_010902	CDS	scaffold_6	2006675	2010848	-	PF03033	GT1
NEMBOFW57_000088	CDS	scaffold_1	298021	299754	-	PF01793	GT15
NEMBOFW57_004562	CDS	scaffold_1	14736773	14739410	+	PF01793;PF07690	GT15
NEMBOFW57_005272	CDS	scaffold_2	1650971	1652340	-	PF01793	GT15
NEMBOFW57_006806	CDS	scaffold_3	1262905	1264404	+	PF01793	GT15
NEMBOFW57_002649	CDS	scaffold_1	8656308	8657361	+	PF04724	GT17
NEMBOFW57_002417	CDS	scaffold_1	7890452	7894184	+	PF03142;PF13632;PF13641	GT2
NEMBOFW57_003117	CDS	scaffold_1	10103403	10106260	-	PF01644;PF08407	GT2
NEMBOFW57_003425	CDS	scaffold_1	11120996	11123251	+	PF13632	GT2
NEMBOFW57_003476	CDS	scaffold_1	11292449	11293779	-	PF13506;PF13641	GT2
NEMBOFW57_003751	CDS	scaffold_1	12081064	12083565	+	PF00535;PF13506;PF13641	GT2
NEMBOFW57_004946	CDS	scaffold_2	597131	599996	+	PF01644;PF08407	GT2
NEMBOFW57_006034	CDS	scaffold_2	4048666	4051695	+	PF13632	GT2
NEMBOFW57_006049	CDS	scaffold_2	4108358	4111017	+	PF01644;PF08407	GT2
NEMBOFW57_006448	CDS	scaffold_3	120927	124414	-	PF01644;PF08407	GT2
NEMBOFW57_007490	CDS	scaffold_3	3390553	3391951	+		GT2
NEMBOFW57_007560	CDS	scaffold_3	3629154	3631982	-	PF03142;PF13632;PF13641	GT2
NEMBOFW57_007626	CDS	scaffold_3	3841121	3846886	-	PF00063;PF00173;PF03142;PF08766;PF13632;PF13641	GT2
NEMBOFW57_007627	CDS	scaffold_3	3850297	3855869	+	PF00173;PF03142;PF08766;PF13632;PF13641	GT2
NEMBOFW57_007648	CDS	scaffold_3	3907256	3908091	-	PF00535;PF13641	GT2
NEMBOFW57_009453	CDS	scaffold_5	764630	767247	+	PF01644;PF08407	GT2
NEMBOFW57_002838	CDS	scaffold_1	9236650	9239566	-	PF00982;PF02358	GT20
NEMBOFW57_004244	CDS	scaffold_1	13591500	13593214	-	PF00982	GT20
NEMBOFW57_010825	CDS	scaffold_6	1718408	1721689	-	PF00982;PF02358	GT20
NEMBOFW57_005587	CDS	scaffold_2	2619479	2621129	-		GT21
NEMBOFW57_000254	CDS	scaffold_1	831045	833158	-		GT22
NEMBOFW57_000697	CDS	scaffold_1	2281949	2283818	+		GT22
NEMBOFW57_002714	CDS	scaffold_1	8849952	8852130	+		GT22
NEMBOFW57_006908	CDS	scaffold_3	1548114	1550165	-	PF03901	GT22
NEMBOFW57_010559	CDS	scaffold_6	841935	846478	-	PF06427;PF18400;PF18401;PF18402;PF18403;PF18404	GT24
NEMBOFW57_008970	CDS	scaffold_4	3350764	3352108	-		GT25
NEMBOFW57_005432	CDS	scaffold_2	2159953	2162329	-	PF05693	GT3
NEMBOFW57_005553	CDS	scaffold_2	2515796	2517182	+		GT31
NEMBOFW57_009317	CDS	scaffold_5	334750	336541	+		GT31
NEMBOFW57_009398	CDS	scaffold_5	581882	583586	+		GT31
NEMBOFW57_010362	CDS	scaffold_6	214896	216498	-		GT31
NEMBOFW57_010834	CDS	scaffold_6	1747407	1748496	+	PF01762	GT31
NEMBOFW57_010975	CDS	scaffold_6	2234514	2235798	+		GT31
NEMBOFW57_010985	CDS	scaffold_6	2268098	2269008	+		GT31

NEMBOFW57_001072	CDS	scaffold_1	3512184	3514025	-	PF04488	GT32
NEMBOFW57_001141	CDS	scaffold_1	3723412	3724531	-	PF04488	GT32
NEMBOFW57_001904	CDS	scaffold_1	6237444	6238639	-	PF04488	GT32
NEMBOFW57_006411	CDS	scaffold_3	20640	21992	+	PF04488	GT32
NEMBOFW57_006935	CDS	scaffold_3	1624574	1626014	+	PF04488	GT32
NEMBOFW57_007112	CDS	scaffold_3	2120771	2122042	+		GT33
NEMBOFW57_002427	CDS	scaffold_1	7935090	7936468	+	PF05637	GT34
NEMBOFW57_004433	CDS	scaffold_1	14279384	14280460	-	PF05637	GT34
NEMBOFW57_006478	CDS	scaffold_3	209540	213847	-	PF00343	GT35
NEMBOFW57_001537	CDS	scaffold_1	5013267	5016656	-	PF02366;PF02815;PF16192	GT39
NEMBOFW57_002406	CDS	scaffold_1	7841523	7843973	+	PF02366;PF02815;PF16192	GT39
NEMBOFW57_004357	CDS	scaffold_1	14010692	14013733	+	PF02366;PF02815;PF16192	GT39
NEMBOFW57_003524	CDS	scaffold_1	11450094	11451675	+	PF00534;PF13524;PF13692	GT4
NEMBOFW57_004042	CDS	scaffold_1	12993798	12994646	+		GT4
NEMBOFW57_004043	CDS	scaffold_1	12995298	12996839	+		GT4
NEMBOFW57_004463	CDS	scaffold_1	14358373	14360297	-	PF00534;PF13692;PF15924	GT4
NEMBOFW57_004605	CDS	scaffold_1	14850979	14852509	-	PF00534;PF08288;PF13439;PF13477;PF13579;PF13692	GT4
NEMBOFW57_006229	CDS	scaffold_2	4661743	4664404	+	PF00534;PF13692	GT4
NEMBOFW57_008169	CDS	scaffold_4	756219	757681	-	PF13692	GT4
NEMBOFW57_009623	CDS	scaffold_5	1473163	1475984	+	PF00534;PF13692	GT4
NEMBOFW57_010183	CDS	scaffold_5	3270171	3271672	-	PF00534;PF13439;PF13524;PF13579;PF13692	GT4
NEMBOFW57_005159	CDS	scaffold_2	1284293	1287929	-	PF13181;PF13374;PF13432	GT41
NEMBOFW57_003918	CDS	scaffold_1	12603298	12609207	-	PF02364;PF14288	GT48
NEMBOFW57_010425	CDS	scaffold_6	403570	405078	+		GT50
NEMBOFW57_000063	CDS	scaffold_1	215126	217004	+	PF09488	GT55
NEMBOFW57_000611	CDS	scaffold_1	2013974	2015657	-	PF03155	GT57
NEMBOFW57_003153	CDS	scaffold_1	10196709	10198572	+		GT57
NEMBOFW57_006814	CDS	scaffold_3	1287151	1288640	-	PF05208	GT58
NEMBOFW57_006692	CDS	scaffold_3	931823	935273	-	PF09796	GT59
NEMBOFW57_003191	CDS	scaffold_1	10333989	10335160	+	PF03452	GT62
NEMBOFW57_006153	CDS	scaffold_2	4414027	4415548	+	PF03452	GT62
NEMBOFW57_007328	CDS	scaffold_3	2815038	2816627	-	PF03452	GT62
NEMBOFW57_002281	CDS	scaffold_1	7415785	7418362	+	PF02516	GT66
NEMBOFW57_006579	CDS	scaffold_3	524947	526190	+	PF11735	GT69
NEMBOFW57_006940	CDS	scaffold_3	1634416	1635764	-	PF11735	GT69
NEMBOFW57_008587	CDS	scaffold_4	2113403	2114631	-	PF11735	GT69
NEMBOFW57_009685	CDS	scaffold_5	1661972	1663337	+	PF11735	GT69
NEMBOFW57_001281	CDS	scaffold_1	4218004	4219689	-	PF11051	GT71
NEMBOFW57_003717	CDS	scaffold_1	11990114	11991587	-		GT71
NEMBOFW57_005706	CDS	scaffold_2	2972187	2973660	-		GT71
NEMBOFW57_009551	CDS	scaffold_5	1207407	1208210	-		GT71
NEMBOFW57_008522	CDS	scaffold_4	1862100	1863655	+	PF04188	GT76
NEMBOFW57_005425	CDS	scaffold_2	2128444	2131512	+	PF01501	GT8
NEMBOFW57_006139	CDS	scaffold_2	4381851	4382989	-		GT8
NEMBOFW57_000381	CDS	scaffold_1	1260782	1262720	-	PF05686	GT90

NEMBOFW57_003542	CDS	scaffold_1	11497496	11499357	-		GT90
NEMBOFW57_010590	CDS	scaffold_6	959476	962745	-		GT90
NEMBOFW57_004511	CDS	scaffold_1	14557716	14561900	-	PF03443	GT90+AA9
NEMBOFW57_007080	CDS	scaffold_3	2028810	2030152	+		PL1
NEMBOFW57_008589	CDS	scaffold_4	2119690	2120881	-	PF00544	PL1
NEMBOFW57_009693	CDS	scaffold_5	1687722	1688852	-	PF00544	PL1
NEMBOFW57_010632	CDS	scaffold_6	1193775	1194841	+	PF00544	PL1
NEMBOFW57_010773	CDS	scaffold_6	1590555	1591798	+	PF00544	PL1
NEMBOFW57_009195	CDS	scaffold_4	4010376	4011129	-	PF14099	PL20
NEMBOFW57_004480	CDS	scaffold_1	14414342	14417093	-		PL26
NEMBOFW57_009003	CDS	scaffold_4	3468560	3469773	+	PF03211	PL3
NEMBOFW57_010715	CDS	scaffold_6	1417147	1417958	-	PF03211	PL3
NEMBOFW57_001370	CDS	scaffold_1	4478411	4483301	-	PF07732;PF09284;PF14683;PF14686	PL4
NEMBOFW57_006439	CDS	scaffold_3	94704	96819	+	PF14683;PF14686	PL4
NEMBOFW57_008717	CDS	scaffold_4	2543140	2544825	+	PF09284;PF14683;PF14686	PL4
NEMBOFW57_009327	CDS	scaffold_5	360754	362920	-	PF14683;PF14686	PL4
NEMBOFW57_005696	CDS	scaffold_2	2934466	2938455	+	PF08787	PL7
NEMBOFW57_001223	CDS	scaffold_1	3971032	3972316	-	PF13229	PL9

Supplementary Table S2: Functional prediction of the CAZymes found by in-gel MS on different chitin-like and complex substrates: maize leaves (MZ) or sugar cane bagasse (SCB). The proteins were separated by SDS-PAGE followed by in-gel tryptic digestion and LC-MS/MS. The accession number and HMMER, Hotpep, and DIAMOND annotations are given. For the EC classification, a PBLAST search was performed and the top 1, 10, 100 and 99999 EC classifications were fetched for the hits. For ambiguous or unknown CAZyme family prediction results, BLASTP annotations was used to match the CAZyme family.

#	Accession number	MZ	SCB	CS			C	HMMER	Hotpep	DIAMOND	Annotation	top_1	top_10	top_100	top_99999	Function
				L	M	H										
0	NEMBOFW57_010300	x	x					AA1_3(52-366)	AA1(5)	AA1_3+1.10.3.2	AA1_3					Laccase / *p*-diphenol:oxygen oxidoreductase / ferroxidase (EC 1.10.3.2); ferroxidase (EC 1.10.3.-); Laccase-like multicopper oxidase (EC 1.10.3.-)
1	NEMBOFW57_001206		x					AA1_3(67-379)	AA1(25)	AA1_3	AA1_3					Laccase / *p*-diphenol:oxygen oxidoreductase / ferroxidase (EC 1.10.3.2); ferroxidase (EC 1.10.3.-); Laccase-like multicopper oxidase (EC 1.10.3.-)

**2**	NEMBOFW57_005252			x				AA2(65-241)	AA0(2)	AA0	AA2				2.4.2.26|3.2.1.58	Manganese peroxidase (EC 1.11.1.13); versatile peroxidase (EC 1.11.1.16); lignin peroxidase (EC 1.11.1.14); peroxidase (EC 1.11.1.-); cytochrome-c peroxidase (EC 1.11.1.5); ascorbate peroxidase (EC 1.11.1.11)
**3**	NEMBOFW57_003132	x	x					AA2(88-339)	-	-	AA2					Manganese peroxidase (EC 1.11.1.13); versatile peroxidase (EC 1.11.1.16); lignin peroxidase (EC 1.11.1.14); peroxidase (EC 1.11.1.-); cytochrome-c peroxidase (EC 1.11.1.5); ascorbate peroxidase (EC 1.11.1.11)
**4**	NEMBOFW57_010319	x	x	x	x	x	x	AA3(14-592)	AA3(6)	AA3	AA3				1.1.3.13|1.1.3.16|1.1.3.7	Aryl alcohol oxidase (EC 1.1.3.7); alcohol oxidase (EC 1.1.3.13)
**5**	NEMBOFW57_001352	x	x					AA3(35-616)	AA3(6)	AA3	AA3				1.1.3.13|1.1.3.16|1.1.3.7	Aryl alcohol oxidase (EC 1.1.3.7); alcohol oxidase (EC 1.1.3.13)
**6**	NEMBOFW57_008032	x	x					AA3_2(1-498)	AA3(17)	AA3_2	AA3_2				1.1.3.13|1.1.3.16|1.1.3.4|1.1.3.7	Glucose 1-oxidase (EC 1.1.3.4); aryl alcohol oxidase (EC 1.1.3.7); alcohol oxidase (EC 1.1.3.13)
**7**	NEMBOFW57_007947	x		x		x		AA3_2(152-518)	AA3(3)	AA3_2	AA3_2				1.1.3.13|1.1.3.16|1.1.3.4|1.1.3.7	Glucose 1-oxidase (EC 1.1.3.4); aryl alcohol oxidase (EC 1.1.3.7); alcohol oxidase (EC 1.1.3.13)
**8**	NEMBOFW57_009225	x						AA3_2(24-312)	AA3(3)	AA3_2	AA3_2			1.1.3.4	1.1.3.13|1.1.3.4|1.1.3.7	Glucose 1-oxidase (EC 1.1.3.4)

**9**	NEMBOFW57_007166		x				x	AA3_2(24-623)	AA3(6)	AA3	AA3_2				1.1.3.16|1.1.3.7	Aryl alcohol oxidase (EC 1.1.3.7)
**10**	NEMBOFW57_007796		x					AA3_2(24-641)	AA3(6)	AA3	AA3_2					Cellobiose dehydrogenase (EC 1.1.99.18); glucose 1-oxidase (EC 1.1.3.4); aryl alcohol oxidase (EC 1.1.3.7); alcohol oxidase (EC 1.1.3.13); pyranose oxidase (EC 1.1.3.10)
**11**	NEMBOFW57_010602	x	x	x	x	x	x	AA3_2(25-585)	AA3(2)	AA3_2	AA3_2				1.1.3.13|1.1.3.4|1.1.3.7	Glucose 1-oxidase (EC 1.1.3.4); aryl alcohol oxidase (EC 1.1.3.7); alcohol oxidase (EC 1.1.3.13)
**12**	NEMBOFW57_010289	x	x	x	x	x		AA3_2(28-655)	AA3(6)	AA3	AA3_2				1.1.3.13|1.1.3.7	Aryl alcohol oxidase (EC 1.1.3.7); alcohol oxidase (EC 1.1.3.13)
**13**	NEMBOFW57_006440	x	x	x	x	x	x	AA3_2(31-611)	AA3(2)+AA8(3)	AA3_2	AA3_2				1.1.3.13|1.1.3.16|1.1.3.4|1.1.3.7	Glucose 1-oxidase (EC 1.1.3.4); aryl alcohol oxidase (EC 1.1.3.7); alcohol oxidase (EC 1.1.3.13)
**14**	NEMBOFW57_002775	x	x					AA3_2(3-258)	-	-	AA3_2				1.1.3.16|1.1.3.4|1.1.3.7	Glucose 1-oxidase (EC 1.1.3.4); aryl alcohol oxidase (EC 1.1.3.7); alcohol oxidase (EC 1.1.3.13)
**15**	NEMBOFW57_008083	x	x					AA3_2(34-636)	AA3(17)	AA3_2	AA3_2				1.1.3.4|1.1.3.7	Glucose 1-oxidase (EC 1.1.3.4); aryl alcohol oxidase (EC 1.1.3.7)
**16**	NEMBOFW57_010449	x	x	x	x			AA3_2(41-622)	AA3(17)	AA3_2	AA3_2				1.1.3.13|1.1.3.16|1.1.3.4|1.1.3.7	Glucose 1-oxidase (EC 1.1.3.4); aryl alcohol oxidase (EC 1.1.3.7); alcohol oxidase (EC 1.1.3.13)

<table>
<tr><td>17</td><td>NEMBOFW57_004634</td><td>x</td><td></td><td>x</td><td>x</td><td></td><td></td><td>AA3_2(41-671)</td><td>AA3(6)</td><td>AA3</td><td>AA3_2</td><td></td><td></td><td></td><td>1.1.3.13|1.1.3.7</td><td>Aryl alcohol oxidase (EC 1.1.3.7); alcohol oxidase (EC 1.1.3.13)</td></tr>
<tr><td>18</td><td>NEMBOFW57_010418</td><td></td><td></td><td>x</td><td>x</td><td></td><td>x</td><td>AA3_2(9-604)</td><td>AA3(28)</td><td>AA3_2</td><td>AA3_2</td><td></td><td></td><td></td><td>1.1.3.13|1.1.3.4|1.1.3.7</td><td>Glucose 1-oxidase (EC 1.1.3.4); aryl alcohol oxidase (EC 1.1.3.7); alcohol oxidase (EC 1.1.3.13)</td></tr>
<tr><td>19</td><td>NEMBOFW57_010246</td><td>x</td><td>x</td><td></td><td></td><td></td><td></td><td>AA8(22-540)</td><td>AA3(5)+AA8(1)+CBM1(10)</td><td>AA3_1</td><td>AA3+AA8</td><td></td><td></td><td></td><td>1.1.3.13|1.1.3.16|1.1.3.7</td><td>Aryl alcohol oxidase (EC 1.1.3.7); alcohol oxidase (EC 1.1.3.13) + Iron reductase domain</td></tr>
<tr><td>20</td><td>NEMBOFW57_008624</td><td>x</td><td>x</td><td></td><td></td><td></td><td></td><td>AA8(4-767)</td><td>AA3(5)+AA8(1)+CBM1(10)</td><td>AA3_1+AA8</td><td>AA3+AA8</td><td></td><td></td><td></td><td>1.1.3.13|1.1.3.7</td><td>Aryl alcohol oxidase (EC 1.1.3.7); alcohol oxidase (EC 1.1.3.13) + Iron reductase domain</td></tr>
<tr><td>21</td><td>NEMBOFW57_001011</td><td>x</td><td>x</td><td></td><td></td><td></td><td></td><td>AA8(3-829)</td><td>AA3(5)+AA8(1)+CBM1(10)</td><td>AA3_1+AA8+CBM1+1.1.99.18</td><td>AA3+AA8+CBM1</td><td></td><td></td><td></td><td>1.-.-.-|1.1.3.13|1.1.3.7|3.2.1.78|3.2.1.8</td><td>Aryl alcohol oxidase (EC 1.1.3.7); alcohol oxidase (EC 1.1.3.13) + Iron reductase domain + these modules cellulose binding function has been demonstrated</td></tr>
<tr><td>22</td><td>NEMBOFW57_003104</td><td>x</td><td>x</td><td>x</td><td>x</td><td>x</td><td>x</td><td>-</td><td>AA5(5)</td><td>AA5_1</td><td>AA5_1</td><td></td><td></td><td></td><td>1.1.3.47|1.1.3.7|1.1.3.9|1.2.3.15|2.4.2.26|3.2.1.58</td><td>Galactose oxidase (EC 1.1.3.9); glyoxal oxidase (EC 1.2.3.15)</td></tr>
<tr><td>23</td><td>NEMBOFW57_002928</td><td>x</td><td>x</td><td></td><td></td><td></td><td></td><td>AA6(4-201)</td><td>AA6(1)</td><td>AA6</td><td>AA6</td><td></td><td></td><td>1.6.5.6</td><td>1.6.5.6</td><td>1,4-Benzoquinone reductase (EC. 1.6.5.6)</td></tr>
<tr><td>24</td><td>NEMBOFW57_001310</td><td>x</td><td>x</td><td>x</td><td>x</td><td>x</td><td>x</td><td>-</td><td>AA7(3)</td><td>-</td><td>AA7</td><td></td><td></td><td>1.1.3.-</td><td>1.1.3.-</td><td>Glucooligosaccharide oxidase (EC 1.1.3.-); chitooligosaccharide oxidase (EC 1.1.3.-);</td></tr>
</table>

**25**	NEMBOFW57_001116	x	x					AA7(129-552)	-	-	AA7			1.1.3.-|3.2.1.58	1.1.3.-|3.2.1.58	Glucooligosaccharide oxidase (EC 1.1.3.-); chitooligosaccharide oxidase (EC 1.1.3.-);
**26**	NEMBOFW57_001464	x						AA7(154-578)	-	-	AA7		1.1.3.-	1.1.3.-	1.1.3.-	Glucooligosaccharide oxidase (EC 1.1.3.-); chitooligosaccharide oxidase (EC 1.1.3.-);
**27**	NEMBOFW57_004115	x	x					AA7(51-487)	AA7(2)	AA7	AA7		1.1.3.-	1.1.3.-	1.1.3.-	Glucooligosaccharide oxidase (EC 1.1.3.-); chitooligosaccharide oxidase (EC 1.1.3.-);
**28**	NEMBOFW57_008561	x	x	x	x	x		AA7(58-266)	-	-	AA7		1.1.3.-	1.1.3.-	1.1.3.-	Glucooligosaccharide oxidase (EC 1.1.3.-); chitooligosaccharide oxidase (EC 1.1.3.-);
**29**	NEMBOFW57_008720	x	x					-	AA8(4)	AA8	AA8				1.-.-.-	Oxidoreductase (EC 1.-.-.-)
**30**	NEMBOFW57_007876	x	x					AA9(10-229)	AA9(9)	AA9	AA9					Lytic cellulose monooxygenase (C1-hydroxylating) (EC 1.14.99.54); lytic cellulose monooxygenase (C4-dehydrogenating) (EC 1.14.99.56)
**31**	NEMBOFW57_001172	x						AA9(15-228)	AA9(7)	AA9	AA9					Lytic cellulose monooxygenase (C1-hydroxylating) (EC 1.14.99.54); lytic cellulose monooxygenase (C4-dehydrogenating) (EC 1.14.99.56)
**32**	NEMBOFW57_008059					x		AA9(4-221)	AA9(2)+CBM1(38)	AA9	AA9					Lytic cellulose monooxygenase (C1-hydroxylating) (EC 1.14.99.54); lytic cellulose monooxygenase (C4-dehydrogenating) (EC 1.14.99.56)

33	NEMBOFW57_005701	x						AA9(4-222)	AA9(2)+CBM1(38)	AA9	AA9					Lytic cellulose monooxygenase (C1-hydroxylating) (EC 1.14.99.54); lytic cellulose monooxygenase (C4-dehydrogenating) (EC 1.14.99.56)
34	NEMBOFW57_010245		x				x	AA9(6-209)	AA9(2)	AA9	AA9					Lytic cellulose monooxygenase (C1-hydroxylating) (EC 1.14.99.54); lytic cellulose monooxygenase (C4-dehydrogenating) (EC 1.14.99.56)
35	NEMBOFW57_010597				x	x		AA9(7-218)	AA9(5)	AA9	AA9					Lytic cellulose monooxygenase (C1-hydroxylating) (EC 1.14.99.54); lytic cellulose monooxygenase (C4-dehydrogenating) (EC 1.14.99.56)
36	NEMBOFW57_002035		x					AA9(8-210)	AA9(4)+CBM1(15)	AA9+CBM1	AA9					Lytic cellulose monooxygenase (C1-hydroxylating) (EC 1.14.99.54); lytic cellulose monooxygenase (C4-dehydrogenating) (EC 1.14.99.56)
37	NEMBOFW57_003857	x	x					AA9(5-218)	AA9(13)+CBM1(38)	AA9+CBM1	AA9+CBM1					Lytic cellulose monooxygenase (C1-hydroxylating) (EC 1.14.99.54); lytic cellulose monooxygenase (C4-dehydrogenating) (EC 1.14.99.56) + these modules cellulose binding function has been demonstrated
38	NEMBOFW57_001466	x	x					AA9(8-223)	AA9(7)+CBM1(25)	AA9+CBM1	AA9+CBM1					Lytic cellulose monooxygenase (C1-hydroxylating) (EC 1.14.99.54); lytic cellulose monooxygenase (C4-dehydrogenating) (EC 1.14.99.56) + these modules cellulose binding function has been demonstrated

39	NEMBOFW57_001044	x	x	x	x	x		AA9(8-227)	AA9(7)+CBM1(25)	AA9+CBM1	AA9+CBM1					Lytic cellulose monooxygenase (C1-hydroxylating) (EC 1.14.99.54); lytic cellulose monooxygenase (C4-dehydrogenating) (EC 1.14.99.56) + these modules cellulose binding function has been demonstrated
40	NEMBOFW57_008990		x					AA12(333-741)	AA12(1)	AA12	AA12			1.-.-.-|3.2.1.26	1.-.-.-|3.2.1.26	Oxidoreductase (EC 1.-.-.-)
41	NEMBOFW57_010266	x	x					AA16(21-189)	AA16(1)	AA16	AA16					Lytic cellulose monooxygenase (C1-hydroxylating) (EC 1.14.99.54)
42	NEMBOFW57_003584		x					-	CBM2(28)	CBM1	CBM1			3.1.1.-|3.2.1.-|3.2.1.176|3.2.1.4|3.2.1.8	3.1.1.-|3.1.1.6|3.1.1.72|3.2.1.-|3.2.1.14|3.2.1.151|3.2.1.176|3.2.1.4|3.2.1.55|3.2.1.78|3.2.1.8|3.2.1.91|4.2.2.10	These modules cellulose binding function has been demonstrated
43	NEMBOFW57_006922	x	x					-	-	CBM50	CBM50				3.2.1.-|3.2.1.14|3.2.1.17|3.2.1.96	CBM50 chitopentaose binding function has been demonstrated
44	NEMBOFW57_000190	x	x	x	x	x		CBM52(633-683)	CBM52(1)	CBM52	CBM52			3.2.1.39	3.2.1.39	Binding to β-(1,3)-glucan demonstrated
45	NEMBOFW57_000278		x					CE1(23-287)	-	-	CE1				2.3.1.122|2.3.1.20|3.1.1.-	Cinnamoyl esterase (EC 3.1.1.-); diacylglycerol *O*-acyltransferase (EC 2.3.1.20); trehalose 6-*O*-mycolyltransferase (EC 2.3.1.122)

46	NEMBOFW57_009711	x						CE1(29-214)	CE1(14)	CE1	CE1		3.1.1.72	3.1.1.-|3.1.1.72|3.1.1.73	3.1.1.-|3.1.1.6|3.1.1.72|3.1.1.73	Acetyl xylan esterase (EC 3.1.1.72)
47	NEMBOFW57_002054	x	x					CE1(3-170)	CE1(32)	CE1	CE1		3.1.1.73	3.1.1.-|3.1.1.72|3.1.1.73	3.1.1.-|3.1.1.6|3.1.1.72|3.1.1.73	Feruloyl esterase (EC 3.1.1.73)
48	NEMBOFW57_004513	x	x					CE1(32-191)	CE1(32)	CE1	CE1		3.1.1.73	3.1.1.-|3.1.1.72|3.1.1.73	3.1.1.-|3.1.1.6|3.1.1.72|3.1.1.73	Feruloyl esterase (EC 3.1.1.73)
49	NEMBOFW57_009348		x					CE1(46-180)	-	CE1	CE1		3.1.1.73	3.1.1.73	3.1.1.72|3.1.1.73|3.2.1.8	Feruloyl esterase (EC 3.1.1.73)
50	NEMBOFW57_007800	x	x					CE1(54-286)	CE1(14)+CBM2(65)	CE1	CE1		3.1.1.72	3.1.1.-|3.1.1.72|3.1.1.73	3.1.1.-|3.1.1.6|3.1.1.72|3.1.1.73	Acetyl xylan esterase (EC 3.1.1.72)
51	NEMBOFW57_004294		x					-	CE1(25)+CBM1(17)	CBM1+CE1	CE1+CBM1			3.1.1.72|3.1.1.73	1.-.-.-|3.1.1.72|3.1.1.73|3.2.1.132|3.2.1.176|3.2.1.4|3.2.1.78|3.2.1.8|3.2.1.91	Acetyl xylan esterase (EC 3.1.1.72); feruloyl esterase (EC 3.1.1.73) + these modules cellulose binding function has been demonstrated
52	NEMBOFW57_002052		x					CE1(109-262)	CE1(32)+CBM1(11)	CE1	CE1+CBM1		3.1.1.73	3.1.1.-|3.1.1.72|3.1.1.73	3.1.1.-|3.1.1.6|3.1.1.72|3.1.1.73	Feruloyl esterase (EC 3.1.1.73) + these modules cellulose binding function has been demonstrated
53	NEMBOFW57_001166	x						CE1(33-227)	CE1(34)+CBM2(17)	CBM1+CE1	CE1+CBM1			3.1.1.72	3.1.1.-|3.1.1.6|3.1.1.72|3.1.1.73	Acetyl xylan esterase (EC 3.1.1.72) + these modules cellulose binding function has been demonstrated
54	NEMBOFW57_002906		x		x		x	CE1(41-238)	CE1(14)	CBM1+CE1	CE1+CBM1		3.1.1.72	3.1.1.-|3.1.1.72	3.1.1.-|3.1.1.6|3.1.1.72|3.1.1.73	Acetyl xylan esterase (EC 3.1.1.72)

55	NEMBOFW57_007038	x	x					CE2(133-354)	CE2(14)	CBM1+CE2	CE2			3.1.1.-|3.1.1.72|3.2.1.4	3.1.1.-|3.1.1.72|3.2.1.4	Acetyl xylan esterase (EC 3.1.1.72); pectin acetylesterase (EC 3.1.1.-); rhamnogalacturonan acetylesterase (EC 3.1.1.-)
56	NEMBOFW57_008260	x	x					CE3(10-154)	CE3(6)	CBM1+CE3	CE3		3.1.1.6	3.1.1.6	3.1.1.6|3.1.1.72	Acetylesterase (EC 3.1.1.6) active on various carbohydrate acetyl esters
57	NEMBOFW57_009085	x	x	x	x			CE3(31-220)	CE3(6)+CBM2(48)	CE3	CE3			3.1.1.6	3.1.1.6|3.1.1.72	Acetylesterase (EC 3.1.1.6) active on various carbohydrate acetyl esters
58	NEMBOFW57_005718	x	x					CE4(35-159)	-	CE4	CE4			3.1.1.72|3.5.1.41	3.1.1.72|3.2.1.8|3.5.1.41	Acetyl xylan esterase (EC 3.1.1.72); chi-tin deacetylase (EC 3.5.1.41)
59	NEMBOFW57_009578	x	x				x	CE4(44-165)	-	CE4	CE4			3.1.1.72|3.5.1.41	3.1.1.72|3.5.1.41	Acetyl xylan esterase (EC 3.1.1.72); chi-tin deacetylase (EC 3.5.1.41)
60	NEMBOFW57_006469	x	x					CE4(570-690)	-	-	CE4				3.1.1.72	Acetyl xylan esterase (EC 3.1.1.72)
61	NEMBOFW57_000575	x	x					-	CE4(143)+CBM18(7)	CBM18+CE4	CE4+CBM18				3.1.1.72|3.5.1.104|3.5.1.41	Acetyl xylan esterase (EC 3.1.1.72); chi-tin deacetylase (EC 3.5.1.41); chitooli-gosaccharide deacetylase (EC 3.5.1.-) + these modules chitin-binding function has been demonstrated
62	NEMBOFW57_007936	x	x					CE5(27-223)	CE5(10)+CBM1(28)	CE5	CE5		3.1.1.72	3.1.1.72	3.1.1.72	Acetyl xylan esterase (EC 3.1.1.72)

63	NEMBOFW57_010955		x	x	x	x	x	CE5(38-245)	CE5(10)+CBM1(28)	CBM1+CE5	CE5		3.1.1.72	3.1.1.72	3.1.1.72|3.2.1.73|3.2.1.78|3.2.1.8	Acetyl xylan esterase (EC 3.1.1.72)
64	NEMBOFW57_006728	x	x					CE8(37-313)	CE8(20)	CE8	CE8		3.1.1.11	3.1.1.11	3.1.1.11	Pectin methylesterase (EC 3.1.1.11)
65	NEMBOFW57_009275		x					CE10(120-359)	-	-	CE10					Arylesterase (EC 3.1.1.-); carboxyl esterase (EC 3.1.1.3); acetylcholinesterase (EC 3.1.1.7); cholinesterase (EC 3.1.1.8); sterol esterase (EC 3.1.1.13)
66	NEMBOFW57_001299	x	x					CE10(142-299)	-	-	CE10			3.1.1.6|3.1.1.73	3.1.1.6|3.1.1.73	Feruloyl esterase (EC 3.1.1.73); Acetylesterase (EC 3.1.1.6)
67	NEMBOFW57_000887	x	x		x	x		CE10(233-378)	-	-	CE10					Arylesterase (EC 3.1.1.-); carboxyl esterase (EC 3.1.1.3); acetylcholinesterase (EC 3.1.1.7); cholinesterase (EC 3.1.1.8); sterol esterase (EC 3.1.1.13); brefeldin A esterase (EC 3.1.1.-)
68	NEMBOFW57_002792		x					CE10(489-695)	-	-	CE10					Arylesterase (EC 3.1.1.-); carboxyl esterase (EC 3.1.1.3); acetylcholinesterase (EC 3.1.1.7); cholinesterase (EC 3.1.1.8); sterol esterase (EC 3.1.1.13); brefeldin A esterase (EC 3.1.1.-)
69	NEMBOFW57_002197	x	x					CE10(50-219)	-	-	CE10					Cholinesterase (EC 3.1.1.8)

<table>
<tr><td>70</td><td>NEMBOFW57_000286</td><td>x</td><td>x</td><td></td><td></td><td></td><td></td><td>CE12(307-494)</td><td>CE12(14)</td><td>CE12</td><td>CE12</td><td></td><td></td><td></td><td>3.1.1.72</td><td>Acetyl xylan esterase (EC 3.1.1.72)</td></tr>
<tr><td>71</td><td>NEMBOFW57_001439</td><td>x</td><td>x</td><td></td><td></td><td></td><td></td><td>CE12(31-240)</td><td>CE12(13)</td><td>CE12</td><td>CE12</td><td></td><td></td><td>3.1.1.-</td><td>3.1.1.-</td><td>Pectin acetylesterase (EC 3.1.1.-); rhamnogalacturonan acetylesterase (EC 3.1.1.-)</td></tr>
<tr><td>72</td><td>NEMBOFW57_004062</td><td>x</td><td>x</td><td></td><td></td><td></td><td></td><td>CE15(44-365)</td><td>CE15(2)+CBM1(20)</td><td>CE15</td><td>CE15</td><td></td><td>3.1.1.-</td><td>3.1.1.-</td><td>3.1.1.-|3.1.1.72|3.2.1.39|3.2.1.40</td><td>Pectin acetylesterase (EC 3.1.1.-); rhamnogalacturonan acetylesterase (EC 3.1.1.-)</td></tr>
<tr><td>73</td><td>NEMBOFW57_004373</td><td>x</td><td></td><td></td><td></td><td></td><td></td><td>CE15(45-364)</td><td>CE15(2)+CBM1(20)+CBM2(40)</td><td>CBM1+CE15+3.1.1.-</td><td>CE15</td><td>3.1.1.-</td><td>3.1.1.-</td><td>3.1.1.-</td><td>3.1.1.-|3.1.1.72|3.2.1.40</td><td>Pectin acetylesterase (EC 3.1.1.-); rhamnogalacturonan acetylesterase (EC 3.1.1.-)</td></tr>
<tr><td>74</td><td>NEMBOFW57_003741</td><td>x</td><td>x</td><td>x</td><td>x</td><td></td><td>x</td><td>CE15(66-380)</td><td>CE15(7)</td><td>CE15</td><td>CE15</td><td></td><td>3.1.1.-</td><td>3.1.1.-|3.1.1.72</td><td>3.1.1.-|3.1.1.72|3.2.1.40</td><td>Pectin acetylesterase (EC 3.1.1.-); rhamnogalacturonan acetylesterase (EC 3.1.1.-)</td></tr>
<tr><td>75</td><td>NEMBOFW57_004792</td><td></td><td>x</td><td>x</td><td></td><td>x</td><td></td><td>CE15(86-391)</td><td>CE15(2)+CBM1(20)</td><td>CBM1+CE15+3.1.1.-</td><td>CE15</td><td>3.1.1.-</td><td>3.1.1.-</td><td>3.1.1.-</td><td>3.1.1.-|3.1.1.72|3.2.1.40</td><td>Pectin acetylesterase (EC 3.1.1.-); rhamnogalacturonan acetylesterase (EC 3.1.1.-)</td></tr>
<tr><td>76</td><td>NEMBOFW57_006599</td><td>x</td><td>x</td><td>x</td><td>x</td><td>x</td><td></td><td>CE16(25-325)</td><td>CE16(2)</td><td>CE16</td><td>CE16</td><td></td><td>3.1.1.6</td><td>3.1.1.6</td><td>3.1.1.6|3.2.1.51</td><td>Acetylesterase (EC 3.1.1.6) active on various carbohydrate acetyl esters</td></tr>
<tr><td>77</td><td>NEMBOFW57_009601</td><td></td><td>x</td><td></td><td></td><td></td><td></td><td>CE16(131-388)</td><td>CE16(1)+CBM1(31)</td><td>CBM1+CE16</td><td>CE16+CBM1</td><td></td><td>3.1.1.6</td><td>3.1.1.-|3.1.1.6</td><td>1.-.-.-|3.1.1.-|3.1.1.6|3.1.1.72|3.2.1.-|3.2.1.151|3.2.1.176|3.2.1.21|3.2.1.4|3.2.1.55|3.2.1.78|3.2.1.8|3.2.1.91</td><td>Acetylesterase (EC 3.1.1.6) active on various carbohydrate acetyl esters + these modules cellulose binding function has been demonstrated</td></tr>
</table>

**78**	NEMBOFW57_000322	x	x					GH1(2-468)	GH1(55)	GH1+3.2.1.21	GH1	3.2.1.21	3.2.1.21	3.2.1.21	2.4.1.-|3.2.1.117|3.2.1.118|3.2.1.119|3.2.1.125|3.2.1.149|3.2.1.161|3.2.1.21|3.2.1.25|3.2.1.38|3.2.1.74	β-Glucosidase (EC 3.2.1.21)
**79**	NEMBOFW57_010681		x					GH2(103-611)	GH2(70)	GH2	GH2			3.2.1.165	3.2.1.165	Exo-β-glucosaminidase (EC 3.2.1.165)
**80**	NEMBOFW57_009152	x	x			x		GH2(30-644)	GH2(36)	GH2	GH2			3.2.1.23	3.2.1.23	β-Galactosidase (EC 3.2.1.23)
**81**	NEMBOFW57_004374	x	x					GH2(34-670)	GH2(36)	GH2	GH2			3.2.1.23	3.2.1.23	β-Galactosidase (EC 3.2.1.23)
**82**	NEMBOFW57_002850		x		x			GH2(39-855)	GH2(10)+CBM32(79)+CBM51(5)+CBM67(4)	GH2	GH2+CBM32+CBM51+CBM67			3.2.1.23	3.2.1.23	β-Galactosidase (EC 3.2.1.23) + CBM32 binding to polygalacturonic acid, CBM51 Bbnding to galactose, CBM67 L-rhamnose binding activity has been demonstrated.
**83**	NEMBOFW57_002037	x	x	x	x	x	x	GH2(8-378)	GH2(52)+CBM42(2)+CBM67(18)	GH2	GH2+CBM42+CBM67				3.2.1.146	β-D-Galactofuranosidase (EC 3.2.1.146) + CBM42 binding to arabinofuranose, CBM67 L-rhamnose binding activity has been demonstrated.
**84**	NEMBOFW57_002908	x	x					-	GH3(58)	GH3	GH3		3.2.1.37	3.2.1.37|3.2.1.55|3.2.1.8	3.2.1.37|3.2.1.55|3.2.1.8	Xylan β-(1,4)-xylosidase (EC 3.2.1.37)

**85**	NEMBOFW57_005247	x	x					GH3(112-325)	GH3(113)+CBM1(30)	GH3	GH3			3.2.1.21	3.2.1.-|3.2.1.21|3.2.1.37	β-Glucosidase (EC 3.2.1.21)
**86**	NEMBOFW57_009222	x	x					GH3(112-337)	GH3(41)	GH3	GH3		3.2.1.21	3.2.1.21|3.2.1.37	3.2.1.21|3.2.1.37|3.2.1.55	β-Glucosidase (EC 3.2.1.21)
**87**	NEMBOFW57_008747		x					GH3(23-237)	GH3(63)+CBM1(30)	GH3	GH3			3.2.1.21	3.2.1.-|3.2.1.21|3.2.1.37	β-Glucosidase (EC 3.2.1.21)
**88**	NEMBOFW57_002909	x	x					GH3(240-472)	GH3(58)	GH3	GH3		3.2.1.37	3.2.1.37|3.2.1.55	3.2.1.-|3.2.1.37|3.2.1.55|3.2.1.8	Xylan β-(1,4)-xylosidase (EC 3.2.1.37)
**89**	NEMBOFW57_002874	x	x					GH3(3-209)	GH3(58)	GH3+3.2.1.37	GH3	3.2.1.37	3.2.1.37	3.2.1.37|3.2.1.55|3.2.1.8	3.2.1.-|3.2.1.21|3.2.1.37|3.2.1.55|3.2.1.8	Xylan β-(1,4)-xylosidase (EC 3.2.1.37)
**90**	NEMBOFW57_007883	x	x					GH3(74-281)	GH3(13)+CBM1(30)	GH3	GH3	3.2.1.21	3.2.1.21	3.2.1.-|3.2.1.21	3.2.1.-|3.2.1.21|3.2.1.37	β-Glucosidase (EC 3.2.1.21)
**91**	NEMBOFW57_003505	x	x					GH3(101-321)	GH3(13)+CBM1(30)	GH3+3.2.1.21	GH3+CBM1	3.2.1.21	3.2.1.21	3.2.1.-|3.2.1.21	3.2.1.-|3.2.1.21|3.2.1.37	β-Glucosidase (EC 3.2.1.21) + these modules cellulose binding function has been demonstrated
**92**	NEMBOFW57_001051	x	x	x	x	x		GH3(91-294)	GH3(13)+CBM1(30)	GH3	GH3+CBM1	3.2.1.21	3.2.1.-|3.2.1.21	3.2.1.-|3.2.1.21	3.2.1.-|3.2.1.21|3.2.1.37	β-Glucosidase (EC 3.2.1.21) + these modules cellulose binding function has been demonstrated
**93**	NEMBOFW57_002806		x					CBM1(20-48)+GH5_5(111-388)	GH5(14)+CBM1(5)	CBM1+GH5_5	GH5_5		3.2.1.4	3.2.1.4	3.2.1.4	Endoglucanase (EC 3.2.1.4)

**94**	NEMBOFW57_009318	x	x					GH5_5(70-263)	GH5(14)+CBM1(5)	GH5_5	GH5_5		3.2.1.4	3.2.1.4	3.2.1.4	Endoglucanase (EC 3.2.1.4)
**95**	NEMBOFW57_004293		x					GH5_5(40-338)	GH5(81)+CBM1(8)	CBM1+GH5_5	GH5_5+CBM1		3.2.1.4	3.2.1.4	3.2.1.4	Endoglucanase (EC 3.2.1.4) + these modules cellulose binding function has been demonstrated
**96**	NEMBOFW57_004769	x	x					GH5_7(63-368)	GH5(43)+CBM1(32)	GH5_7	GH5_7	3.2.1.78	3.2.1.78	3.2.1.78	2.4.1.-|3.2.1.78	Mannan endo-β-(1,4)-mannosidase (EC 3.2.1.78)
**97**	NEMBOFW57_009419	x	x					GH5_16(45-382)	GH5(133)	GH5_16	GH5_16		3.2.1.164	3.2.1.164	3.2.1.164|3.2.1.4	Endo-β-(1,6)-galactanase (EC 3.2.1.164)
**98**	NEMBOFW57_001193	x	x					GH5_23(82-360)	-	GH5_23	GH5_23		3.2.1.-|3.2.1.149	3.2.1.-|3.2.1.149|3.2.1.55|3.2.1.58|3.2.1.75	3.2.1.-|3.2.1.149|3.2.1.21|3.2.1.55|3.2.1.58|3.2.1.75	β-Primeverosidase (EC 3.2.1.149); β-(1,3)-mannanase (EC 3.2.1.-); arabinoxylan-specific endo-β-(1,4)-xylanase (EC 3.2.1.-) β-glycosidase (EC 3.2.1.-); β-rutinosidase /α-L-rhamnose-(1,6)-β-D-glucosidase (EC 3.2.1.-); glucomannan-specific endo-Î²-(1,4)-glucanase (EC 3.2.1.-)
**99**	NEMBOFW57_004785		x		x			GH6(156-454)	GH6(4)+CBM1(3)	CBM1+GH6	GH6		3.2.1.91	3.2.1.4|3.2.1.91	3.2.1.-|3.2.1.4|3.2.1.73|3.2.1.8|3.2.1.91	Cellobiohydrolase (EC 3.2.1.91)
**100**	NEMBOFW57_008641	x	x	x	x	x	x	GH6(62-353)	GH6(4)+CBM1(3)+CBM2(3)+CBM3(5)	GH6	GH6		3.2.1.4|3.2.1.91	3.2.1.4|3.2.1.91	3.2.1.-|3.2.1.4|3.2.1.8|3.2.1.91	Endoglucanase (EC 3.2.1.4); cellobiohydrolase (EC 3.2.1.91)

101	NEMBOFW57_010291	x	x		x			GH7(1-157)	GH7(8)	-	GH7		3.2.1.176	3.2.1.176	3.2.1.132|3.2.1.176|3.2.1.4|3.2.1.73	Reducing end-acting cellobiohydrolase (EC 3.2.1.176)
102	NEMBOFW57_003080	x	x					GH7(19-452)	GH7(1)+CBM1(1)	GH7	GH7		3.2.1.176	3.2.1.132|3.2.1.176	3.2.1.132|3.2.1.176|3.2.1.4|3.2.1.73	Reducing end-acting cellobiohydrolase (EC 3.2.1.176)
103	NEMBOFW57_008456		x					GH7(20-453)	GH7(1)+CBM1(1)	GH7	GH7		3.2.1.176	3.2.1.176	3.2.1.132|3.2.1.176|3.2.1.4|3.2.1.73	Reducing end-acting cellobiohydrolase (EC 3.2.1.176)
104	NEMBOFW57_010165	x	x					GH7(21-417)	GH7(2)+CBM1(9)	GH7	GH7		3.2.1.4|3.2.1.73	3.2.1.176|3.2.1.4|3.2.1.73	3.2.1.132|3.2.1.176|3.2.1.4|3.2.1.73	Endo-β-(1,4)-glucanase (EC 3.2.1.4); endo-β-(1,3)/(1,4)-glucanase (EC 3.2.1.73)
105	NEMBOFW57_003636		x					GH7(23-394)	GH7(2)+CBM1(9)	CBM1+GH7	GH7		3.2.1.4	3.2.1.176|3.2.1.4|3.2.1.73	3.2.1.132|3.2.1.176|3.2.1.4|3.2.1.73	Endo-β-(1,4)-glucanase (EC 3.2.1.4)
106	NEMBOFW57_010290	x	x		x			GH7(24-228)	GH7(8)+CBM1(1)	GH7	GH7		3.2.1.176	3.2.1.132|3.2.1.176	3.2.1.132|3.2.1.176	Reducing end-acting cellobiohydrolase (EC 3.2.1.176)
107	NEMBOFW57_009077	x	x	x	x	x	x	GH7(25-416)	GH7(5)+CBM1(1)	GH7	GH7		3.2.1.4	3.2.1.176|3.2.1.4|3.2.1.73	3.2.1.132|3.2.1.176|3.2.1.4|3.2.1.73	Endo-β-(1,4)-glucanase (EC 3.2.1.4)
108	NEMBOFW57_007704		x	x	x	x	x	GH7(20-452)	GH7(1)+CBM1(1)	CBM1+GH7	GH7+CBM1	3.2.1.176	3.2.1.176	3.2.1.132|3.2.1.176	3.2.1.132|3.2.1.176|3.2.1.4	Reducing end-acting cellobiohydrolase (EC 3.2.1.176) + these modules cellulose binding function has been demonstrated
109	NEMBOFW57_006022	x	x					GH10(20-341)	GH10(56)+CBM1(2)+CBM22(2)	GH10	GH10		3.2.1.8	3.2.1.8	3.1.1.73|3.2.1.8	Endo-β-(1,4)-xylanase (EC 3.2.1.8)

**110**	NEMBOFW57_010962	x	x	x		x	x	GH10(25-332)	GH10(56)+CBM1(2)+CBM22(2)+CBM9(7)	CBM1+GH10	GH10		3.2.1.8	3.2.1.8	3.1.1.73|3.2.1.8	Endo-β-(1,4)-xylanase (EC 3.2.1.8)
**111**	NEMBOFW57_008581	x	x					GH10(29-322)	GH10(4)+CBM1(2)	GH10	GH10		3.2.1.8	3.2.1.8	3.2.1.8	Endo-β-(1,4)-xylanase (EC 3.2.1.8)
**112**	NEMBOFW57_004303	x	x	x	x	x	x	GH10(3-313)	GH10(4)+CBM1(2)+CBM2(23)+CBM22(2)+CBM9(1)	GH10	GH10		3.2.1.8	3.2.1.8	3.1.1.73|3.2.1.8	Endo-β-(1,4)-xylanase (EC 3.2.1.8)
**113**	NEMBOFW57_008598	x	x					GH10(36-351)	GH10(4)+CBM1(2)	GH10	GH10		3.2.1.8	3.2.1.8	3.1.1.73|3.2.1.8	Endo-β-(1,4)-xylanase (EC 3.2.1.8)
**114**	NEMBOFW57_001316	x	x	x	x	x	x	GH10(41-355)	GH10(56)+CBM1(2)+CBM2(23)+CBM22(2)+CBM9(7)	GH10	GH10		3.2.1.8	3.2.1.8	3.2.1.8	Endo-β-(1,4)-xylanase (EC 3.2.1.8)
**115**	NEMBOFW57_003635		x					GH10(24-330)	GH10(58)+CBM1(2)+CBM22(2)+CBM9(7)	CBM1+GH10+3.2.1.8	GH10+CBM1	3.2.1.8	3.2.1.8	3.2.1.8	3.1.1.73|3.2.1.8	Endo-β-(1,4)-xylanase (EC 3.2.1.8) + these modules cellulose binding function has been demonstrated
**116**	NEMBOFW57_009546		x					GH10(25-320)	GH10(4)+CBM1(2)+CBM22(2)+CBM9(7)	CBM1+GH10+3.2.1.8	GH10+CBM1	3.2.1.8	3.2.1.8	3.2.1.8	3.1.1.73|3.2.1.8	Endo-β-(1,4)-xylanase (EC 3.2.1.8) + these modules cellulose binding function has been demonstrated

**117**	NEMBOFW57_006732		x		x			GH10(25-333)	GH10(19)+CBM1(2)+CBM2(23)+CBM22(2)	CBM1+GH10	GH10+CBM1		3.2.1.8	3.2.1.8	3.1.1.73|3.2.1.8	Endo-β-(1,4)-xylanase (EC 3.2.1.8) + these modules cellulose binding function has been demonstrated
**118**	NEMBOFW57_003655	x						GH10(36-335)	GH10(4)+CBM1(2)+CBM2(2)	CBM1+GH10	GH10+CBM1		3.2.1.8	3.2.1.8	3.2.1.8	Endo-β-(1,4)-xylanase (EC 3.2.1.8) + these modules cellulose binding function has been demonstrated
**119**	NEMBOFW57_003719	x	x	x		x	x	GH11(41-217)	CE4(45)+GH11(2)+CBM1(4)+CBM2(27)+CBM36(2)+CBM6(17)+CBM60(1)	GH11+3.2.1.8	GH11	3.2.1.8	3.2.1.8	3.2.1.8	3.2.1.8	Endo-β-(1,4)-xylanase (EC 3.2.1.8)
**120**	NEMBOFW57_008340	x						GH11(48-223)	CE4(45)+GH11(2)+CBM1(4)+CBM10(5)+CBM36(2)+CBM6(17)+CBM60(1)	GH11	GH11		3.2.1.8	3.2.1.8	3.2.1.8	Endo-β-(1,4)-xylanase (EC 3.2.1.8)
**121**	NEMBOFW57_001184	x	x					GH12(98-244)	GH12(6)	GH12	GH12		3.2.1.4	3.2.1.151|3.2.1.4|3.2.1.73	3.2.1.151|3.2.1.4|3.2.1.73	Endoglucanase (EC 3.2.1.4)

122	NEMBOFW57_007690		x					GH12(100-224)+CE1(277-412)	CE1(10)+GH12(6)+CBM2(29)	-	GH12+CE1			3.2.1.4	3.1.1.72|3.1.1.73|3.2.1.151|3.2.1.4|3.2.1.73	Endoglucanase (EC 3.2.1.4) + acetyl xylan esterase (EC 3.1.1.72); feruloyl esterase (EC 3.1.1.73)
123	NEMBOFW57_009282	x		x	x	x	x	GH13_1(59-366)	-	GH13_1	GH13_1		3.2.1.1	2.4.1.25|3.2.1.1|3.2.1.116	2.4.1.-|2.4.1.19|2.4.1.25|3.2.1.1|3.2.1.116|3.2.1.133|3.2.1.2	α-Amylase (EC 3.2.1.1)
124	NEMBOFW57_009709		x		x			GH13_8(250-543)	GH13(94)+CBM48(20)	CBM48+GH13_8	GH13_8+CBM48			2.4.1.18	2.4.1.18	α-(1,4)-Glucan branching enzyme (EC 2.4.1.18) + CBM48 glycogen-binding function, appended to GH13 modules
125	NEMBOFW57_002779			x	x			GH13_25(1094-1547)	GH133(2)	GH13_25+GH133	GH13_25+GH133			2.4.1.25	2.4.1.25|3.2.1.33	4-α-glucanotransferase (EC 2.4.1.25)+ amylo-α-(1,6)-glucosidase (EC 3.2.1.33)
126	NEMBOFW57_004103		x					GH15(140-551)+CBM20(638-728)	GH15(14)+CBM20(5)	CBM20+GH15	GH15+CBM20		3.2.1.3	3.2.1.3	3.2.1.1|3.2.1.3	Glucoamylase (EC 3.2.1.3) + These modules starch-binding binding function has been demonstrated
127	NEMBOFW57_007659	x	x	x	x	x	x	GH15(74-478)+CBM20(530-618)	GH15(14)+CBM20(5)	CBM20+GH15	GH15+CBM20	3.2.1.3	3.2.1.3	3.2.1.3	3.2.1.3	Glucoamylase (EC 3.2.1.3) + These modules starch-binding binding function has been demonstrated
128	NEMBOFW57_009932	x	x	x	x	x	x	GH15(81-489)+CBM20(556-645)	GH15(14)+CBM20(5)	CBM20+GH15+3.2.1.3	GH15+CBM20	3.2.1.3	3.2.1.3	3.2.1.3	3.2.1.1|3.2.1.3	Glucoamylase (EC 3.2.1.3) + These modules starch-binding binding function has been demonstrated
129	NEMBOFW57_009121	x	x					GH16(31-215)	GH16(25)	GH16	GH16		3.2.1.6	3.2.1.39|3.2.1.6	3.2.1.-|3.2.1.39|3.2.1.6|3.2.1.73	Endo-β-(1,3)/(1,4)-glucanase (EC 3.2.1.6)
130	NEMBOFW57_006737		x					GH16(35-283)	GH16(25)+CBM4(7)+CBM6(15)	GH16	GH16		3.2.1.6	3.2.1.6	3.2.1.-|3.2.1.39|3.2.1.6	Endo-β-(1,3)/(1,4)-glucanase (EC 3.2.1.6)

**131**	NEMBOFW57_004987	x	x		x			GH16(37-322)	-	GH16	GH16		3.2.1.73	2.4.1.-|3.2.1.-|3.2.1.39|3.2.1.6|3.2.1.73	2.4.1.-|3.2.1.-|3.2.1.1|3.2.1.35|3.2.1.39|3.2.1.41|3.2.1.6|3.2.1.73	Licheninase (EC 3.2.1.73)
**132**	NEMBOFW57_003172	x			x	x		GH16(41-179)	GH16(22)	GH16	GH16				2.4.1.-|3.2.1.-|3.2.1.39	Endo-β-(1,4)-galactosidase (EC 3.2.1.-); chitin β-(1,6)-glucanosyltransferase (EC 2.4.1.-); β-transglycosidase (EC 2.4.1.-); β-glycosidase (EC 3.2.1.-); β-carrageenase (EC 3.2.1.-)
**133**	NEMBOFW57_008500	x	x	x	x	x	x	GH16(80-221)	GH16(14)	GH16	GH16			2.4.1.-|3.2.1.39	2.4.1.-|3.2.1.-|3.2.1.39	Chitin-β-(1,6)-glucanosyltransferase (EC 2.4.1.-); β-transglycosidase (EC 2.4.1.-); endo-β-(1,3)-glucanase / laminarinase (EC 3.2.1.39)
**134**	NEMBOFW57_000084	x	x	x	x	x	x	GH16(121-264)	GH16(23)+CBM18(8)	CBM18+GH16	GH16+CBM18			2.4.1.-|3.2.1.-	2.4.1.-|3.2.1.-|3.2.1.39	Endo-β-(1,4)-galactosidase (EC 3.2.1.-); chitin-β-(1,6)-glucanosyltransferase (EC 2.4.1.-); β-transglycosidase (EC 2.4.1.-); β-glycosidase (EC 3.2.1.-); β-carrageenase (EC 3.2.1.-) + these modules chitin binding function has been demonstrated
**135**	NEMBOFW57_000164	x	x	x	x	x	x	-	GH17(19)	GH17	GH17				2.4.1.-|3.2.1.-	β-(1,3)-Glucanosyltransglycosylase (EC 2.4.1.-); β-(1,3)-glucosidase (EC 3.2.1.-)
**136**	NEMBOFW57_002419			x	x	x	x	GH17(285-521)	GH17(29)	GH17	GH17		3.2.1.-	3.2.1.-	3.2.1.-	β-(1,3)-Glucosidase (EC 3.2.1.-)

137	NEMBOFW57_007866		x	x	x	x	x	GH18(24-365)	GH18(7)+CBM14(26)	GH18	GH18		3.2.1.14	3.2.1.14	3.2.1.14	Chitinase (EC 3.2.1.14)
138	NEMBOFW57_002998						x	GH18(54-315)	GH18(20)+CBM14(26)	GH18	GH18			3.2.1.14	3.2.1.14	Chitinase (EC 3.2.1.14)
139	NEMBOFW57_004186				x			GH18(9-364)	GH18(20)+CBM14(26)	GH18	GH18		3.2.1.14	3.2.1.14	3.2.1.14	Chitinase (EC 3.2.1.14)
140	NEMBOFW57_006970		x					-	GH18(44)+CBM1(13)	-	GH18+CBM1		3.2.1.14	3.2.1.14	3.2.1.14|3.2.1.17	Chitinase (EC 3.2.1.14) + these modules cellulose binding function has been demonstrated
141	NEMBOFW57_001690	x	x		x			-	-	CBM50+GH18	GH18+CBM50			3.2.1.14	3.2.1.1|3.2.1.14|3.2.1.17|3.2.1.41	Chitinase (EC 3.2.1.14) + chito-pentaose binding function has been demonstrated
142	NEMBOFW57_006926			x	x	x	x	GH18(598-942)	GH18(102)+CBM18(5)	CBM18+CBM50+GH18	GH18+CBM18+CBM50				3.2.1.14	Chitinase (EC 3.2.1.14) + CBM18 chitin binding, CBM50 chitopentaose binding function has been demonstrated
143	NEMBOFW57_003169	x	x	x	x	x		GH20(183-528)	GH20(33)	GH20	GH20			3.2.1.52	3.2.1.52	β-D-Galactofuranosidase (EC 3.2.1.146)
144	NEMBOFW57_004532	x						GH24(103-246)	-	GH24	GH24				3.2.1.17	Lysozyme (EC 3.2.1.17)
145	NEMBOFW57_002920	x	x					CBM35(41-156)+GH26(171-471)	GH26(3)+CBM3(7)+CBM35(3)	CBM35+GH26+3.2.1.78	GH26+CBM35	3.2.1.78	3.2.1.78	3.2.1.151|3.2.1.4|3.2.1.78	3.2.1.100|3.2.1.151|3.2.1.4|3.2.1.73|3.2.1.78|3.2.1.8	β-Mannanase (EC 3.2.1.78) + CBM35 binds to xylan or to mannans and mannooligosaccharides or to β-galactan

146	NEMBOFW57_003773	x	x					-	GH27(15)	GH27	GH27			3.2.1.22	3.2.1.22|3.2.1.88	α-Galactosidase (EC 3.2.1.22)
147	NEMBOFW57_008762	x						GH27(28-221)	GH27(16)	GH27+3.2.1.22	GH27	3.2.1.22	3.2.1.22	3.2.1.22	2.4.1.-|3.2.1.22	α-Galactosidase (EC 3.2.1.22)
148	NEMBOFW57_004007	x						GH28(97-446)	GH28(27)	GH28	GH28		3.2.1.67	3.2.1.67	3.2.1.-|3.2.1.15|3.2.1.67	Exo-polygalacturonase (EC 3.2.1.67)
149	NEMBOFW57_000554				x			GH30_7(35-480)	GH30(13)	GH30_7	GH30_7			3.2.1.-|3.2.1.21|3.2.1.8	3.2.1.-|3.2.1.21|3.2.1.8	Endo-β-(1,4)-xylanase (EC 3.2.1.8); β-glucosidase (3.2.1.21); β-xylosidase [reducing end] (EC 3.2.1.-)
150	NEMBOFW57_009691	x	x					GH31(1205-1731)	GH31(56)	GH31	GH31			3.2.1.20|3.2.1.84	3.2.1.10|3.2.1.177|3.2.1.20|3.2.1.48|3.2.1.84	α-Glucosidase (EC 3.2.1.20); α-(1,3)-glucosidase (EC 3.2.1.84)
151	NEMBOFW57_007820		x					GH31(166-594)	GH31(6)	GH31	GH31					α-xylosidase (EC 3.2.1.177)
152	NEMBOFW57_004860	x	x	x		x	x	GH31(251-780)	GH31(56)	GH31	GH31		3.2.1.20|3.2.1.84	3.2.1.20|3.2.1.84	3.2.1.10|3.2.1.177|3.2.1.20|3.2.1.48|3.2.1.84	α-Glucosidase (EC 3.2.1.20); α-(1,3)-glucosidase (EC 3.2.1.84)
153	NEMBOFW57_009223	x	x		x			GH31(257-697)	GH31(1)	GH31	GH31					α-Xylosidase (EC 3.2.1.177)
154	NEMBOFW57_004148			x	x	x	x	CBM38(42-169)+GH32(228-518)	-	CBM38+GH32+3.2.1.26	GH32+CBM38		3.2.1.26	3.2.1.26	3.2.1.26	Invertase (EC 3.2.1.26) + inulin-binding function has been demonstrated

155	NEMBOFW57_001288	x	x					GH35(33-331)	GH35(3)	GH35	GH35				3.2.1.23	β-Galactosidase (EC 3.2.1.23)
156	NEMBOFW57_001853	x	x					GH35(669-844)	-	GH35	GH35		3.2.1.23	2.4.1.-|3.2.1.-|3.2.1.23	2.4.1.-|3.2.1.-|3.2.1.23	β-Galactosidase (EC 3.2.1.23)
157	NEMBOFW57_003451	x	x	x	x	x		GH37(51-627)	GH37(4)	GH37	GH37			3.2.1.28	3.2.1.28	α,α-Trehalase (EC 3.2.1.28)
158	NEMBOFW57_002602		x					GH38(284-543)	GH38(5)	GH38	GH38			3.2.1.24	3.2.1.24	α-Mannosidase (EC 3.2.1.24)
159	NEMBOFW57_001526	x	x					GH43_1(3-321)	GH43(8)	GH43_1	GH43_1		3.2.1.37	3.2.1.37|3.2.1.55	3.2.1.37|3.2.1.55	β-Xylosidase (EC 3.2.1.37)
160	NEMBOFW57_003627	x						GH43_5(38-315)	GH43(17)	GH43_5	GH43_5			3.2.1.99	3.2.1.99	Endo-α-(1,5)-L-arabinanase [inverting](EC 3.2.1.99)
161	NEMBOFW57_009241	x						GH43_6(33-317)	-	GH43_6+3.2.1.99	GH43_6		3.2.1.99	3.2.1.99	3.2.1.99	Endo-α-(1,5)-L-arabinanase [inverting](EC 3.2.1.99)
162	NEMBOFW57_007945	x	x					GH43_11(4-295)	GH43(27)	GH43_11	GH43_11		3.2.1.37|3.2.1.55	3.2.1.37|3.2.1.55	3.2.1.37|3.2.1.55	β-Xylosidase (EC 3.2.1.37); α-L-Arabinofuranosidase (EC 3.2.1.55)
163	NEMBOFW57_003714	x	x					GH43_14(1-208)	GH43(96)	GH43_14	GH43_14			3.2.1.37	3.2.1.37|3.2.1.55	β-Xylosidase (EC 3.2.1.37)

164	NEMBOFW57_008512	x	x					GH43_14(28-320)	GH43(96)	GH43_14	GH43_14			3.2.1.37	3.2.1.37|3.2.1.55	β-Xylosidase (EC 3.2.1.37)
165	NEMBOFW57_008515	x						GH43_21(97-234)	-	GH43_21	GH43_21			3.2.1.55	3.2.1.55	α-L-Arabinofuranosidase (EC 3.2.1.55)
166	NEMBOFW57_007869	x						GH43_24(44-280)	GH43(103)+CBM13(54)+CBM32(97)+CBM35(21)	CBM35+GH43_24	GH43_24		3.2.1.145	3.2.1.145	3.2.1.145	Exo-β-(1,3)-galactanase (EC 3.2.1.145)
167	NEMBOFW57_004619	x						GH43_24(46-288)	CBM35(35)	CBM35+GH43_24	GH43_24+CBM35			3.2.1.145	3.2.1.145	Exo-β-(1,3)-galactanase (EC 3.2.1.145) + CBM35 binds to xylan or to mannans and mannooligosaccharides or to β-galactan
168	NEMBOFW57_001268	x						GH43_26(28-327)	GH43(6)+CBM42(3)	CBM42+GH43_26+3.2.1.55	GH43_26+CBM42		3.2.1.55	3.2.1.-|3.2.1.55	3.2.1.-|3.2.1.55	α-L-Arabinofuranosidase (EC 3.2.1.55) + Binding to arabinofuranose (present in arabinoxylan) has been demonstrated.
169	NEMBOFW57_009841	x	x					GH43_36(33-275)	GH43(119)	GH43_36	GH43_36				3.2.1.37|3.2.1.55	β-Xylosidase (EC 3.2.1.37); α-L-Arabinofuranosidase (EC 3.2.1.55)
170	NEMBOFW57_000134	x						GH43_36(35-313)	GH43(24)	GH43_36+3.2.1.55	GH43_36		3.2.1.55	3.2.1.55	3.2.1.37|3.2.1.55	α-L-Arabinofuranosidase (EC 3.2.1.55)
171	NEMBOFW57_002500	x	x					GH45(23-224)	GH45(1)+CBM1(6)+CBM10(7)	GH45	GH45		3.2.1.4	3.2.1.4	3.2.1.151|3.2.1.4|3.2.1.78	Endoglucanase (EC 3.2.1.4)

172	NEMBOFW57_009019		x					GH45(24-220)	GH45(1)+CBM1(6)	CBM1+GH45+3.2.1.4	GH45+CBM1		3.2.1.4	3.2.1.4	3.2.1.151|3.2.1.176|3.2.1.4|3.2.1.55|3.2.1.78|3.2.1.8|3.2.1.91	Endoglucanase (EC 3.2.1.4) + these modules cellulose binding function has been demonstrated
173	NEMBOFW57_008471	x	x	x		x		GH47(9-450)	GH47(2)	GH47	GH47		3.2.1.113	3.2.1.113	3.2.1.113	α-Mannosidase (EC 3.2.1.113)
174	NEMBOFW57_009294	x						GH51(14-505)	GH51(7)	GH51	GH51			3.2.1.55	3.2.1.55	α-L-Arabinofuranosidase (EC 3.2.1.55)
175	NEMBOFW57_001185	x	x					GH51(205-644)	GH51(5)	GH51	GH51		3.2.1.55	3.2.1.55	3.2.1.37|3.2.1.55	α-L-Arabinofuranosidase (EC 3.2.1.55)
176	NEMBOFW57_002223	x						GH53(21-346)	GH53(7)	GH53	GH53		3.2.1.89	3.2.1.89	3.2.1.89	Endo-β-(1,4)-galactanase (EC 3.2.1.89)
177	NEMBOFW57_000338	x						GH54(28-342)+CBM42(360-510)	GH54(1)+CBM13(36)+CBM42(1)	CBM42+GH54	GH54+CBM13+CBM42		3.2.1.37|3.2.1.55	3.2.1.37|3.2.1.55	3.2.1.146|3.2.1.20|3.2.1.37|3.2.1.55	α-L-Arabinofuranosidase (EC 3.2.1.55); β-xylosidase (EC 3.2.1.37) + CBM42 binding to arabinofuranose has been demonstrated. CBM13 have been found in a number of other proteins of various functions including glycoside hydrolases and glycosyltransferases
178	NEMBOFW57_008270	x	x					-	-	GH55	GH55			3.2.1.39|3.2.1.58	3.2.1.39|3.2.1.58	Exo-β-(1,3)-glucanase (EC 3.2.1.58); endo-β-(1,3)-glucanase (EC 3.2.1.39)
179	NEMBOFW57_008041	x	x	x	x	x	x	GH55(223-969)	GH55(2)	GH55	GH55			3.2.1.58	3.2.1.39|3.2.1.58	Exo-β-(1,3)-glucanase (EC 3.2.1.58)

**180**	NEMBOFW57_001114	x	x	x	x	x	x	GH55(27-760)	GH0(23)+GH55(2)	GH55	GH55		3.2.1.58	3.2.1.58	3.2.1.39|3.2.1.58	Exo-β-(1,3)-glucanase (EC 3.2.1.58)
**181**	NEMBOFW57_010770			x	x	x		GH55(33-764)	GH55(5)	GH55	GH55		3.2.1.39	3.2.1.39|3.2.1.58	3.2.1.1|3.2.1.39|3.2.1.58	Endo-β-(1,3)-glucanase (EC 3.2.1.39)
**182**	NEMBOFW57_008865		x		x	x		GH55(3-737)	GH55(2)	GH55	GH55			3.2.1.58	3.2.1.39|3.2.1.58	Exo-β-(1,3)-glucanase (EC 3.2.1.58)
**183**	NEMBOFW57_004071	x						GH62(267-496)	GH62(1)+CBM1(23)+CBM13(3)	CBM13+GH62	GH62				3.1.1.72|3.1.1.73|3.2.1.-|3.2.1.55|3.2.1.8	α-L-Arabinofuranosidase (EC 3.2.1.55)
**184**	NEMBOFW57_001269	x	x					GH62(36-324)	GH62(2)+CBM2(69)	GH62+3.2.1.55	GH62	3.2.1.55	3.2.1.55	3.2.1.55	3.1.1.72|3.1.1.73|3.2.1.-|3.2.1.55|3.2.1.8	α-L-Arabinofuranosidase (EC 3.2.1.55)
**185**	NEMBOFW57_009999	x						GH62(30-300)	GH62(1)+CBM1(23)+CBM13(3)	CBM1+GH62	GH62+CBM1		3.2.1.55	3.2.1.55	3.1.1.72|3.1.1.73|3.2.1.-|3.2.1.176|3.2.1.55|3.2.1.8	α-L-Arabinofuranosidase (EC 3.2.1.55) + these modules cellulose binding function has been demonstrated
**186**	NEMBOFW57_004236		x					GH67(17-704)	GH67(3)	GH67	GH67		3.2.1.131|3.2.1.139	3.2.1.131|3.2.1.139	3.2.1.-|3.2.1.131|3.2.1.139	α-Glucuronidase (EC 3.2.1.139); xylan α-(1,2)-glucuronidase (EC 3.2.1.131)
**187**	NEMBOFW57_004454	x	x	x	x	x	x	GH72(16-332)	GH72(3)	GH72	GH72			2.4.1.-	2.4.1.-	β-(1,3)-Glucanosyltransglycosylase (EC 2.4.1.-)
**188**	NEMBOFW57_007083	x	x	x	x	x	x	GH72(31-315)	GH72(3)	GH72	GH72			2.4.1.-	2.4.1.-	β-(1,3)-Glucanosyltransglycosylase (EC 2.4.1.-)

189	NEMBOFW57_005755	x	x	x	x	x	x	GH72(25-328)+CBM43(379-465)	GH72(2)+CBM43(12)	CBM43+GH72	GH72+CBM43			2.4.1.-	2.4.1.-	β-(1,3)-Glucanosyltransglycosylase (EC 2.4.1.-) + the β-(1,3)-glucan binding function has been demonstrated
190	NEMBOFW57_008527	x	x					GH74(82-182)+GH74(570-674)	GH74(1)+CBM1(16)+CBM2(12)	CBM1+GH74	GH74+CBM1		3.2.1.151	3.2.1.151	3.2.1.151	Xyloglucanase (EC 3.2.1.151) + these modules cellulose binding function has been demonstrated
191	NEMBOFW57_006928	x						GH75(8-252)	GH75(2)	GH75	GH75		3.2.1.132	3.2.1.132	3.2.1.132	Chitosanase (EC 3.2.1.132)
192	NEMBOFW57_007041			x	x		x	GH76(25-394)	-	GH76	GH76		3.2.1.101	3.2.1.101	3.2.1.101	α-(1,6)-Mannanase (EC 3.2.1.101)
193	NEMBOFW57_009514	x						GH79(12-368)	GH79(4)	GH79	GH79			3.2.1.-|3.2.1.31	3.2.1.-|3.2.1.166|3.2.1.31	β-Glucuronidase (EC 3.2.1.31); β-4-*O*-methyl-glucuronidase (EC 3.2.1.-)
194	NEMBOFW57_004002	x						GH79(141-369)	GH79(4)	GH79	GH79			3.2.1.-|3.2.1.31	3.2.1.-|3.2.1.166|3.2.1.31	β-Glucuronidase (EC 3.2.1.31); β-4-O-methyl-glucuronidase (EC 3.2.1.-)
195	NEMBOFW57_008132	x						GH79(2-296)	-	GH79	GH79			3.2.1.-|3.2.1.31	3.2.1.-|3.2.1.166|3.2.1.31|3.2.1.36	β-Glucuronidase (EC 3.2.1.31); β-4-O-methyl-glucuronidase (EC 3.2.1.-)
196	NEMBOFW57_003457	x	x					GH79(38-496)	GH79(8)	GH79	GH79		3.2.1.31	3.2.1.-|3.2.1.31	3.2.1.-|3.2.1.166|3.2.1.31	β-Glucuronidase (EC 3.2.1.31)
197	NEMBOFW57_004738	x	x	x	x	x	x	GH79(4-352)+CE1(975-1110)	CE1(25)	GH79	GH79+CE1			3.1.1.73	3.1.1.72|3.1.1.73|3.2.1.-|3.2.1.166|3.2.1.31	β-Glucuronidase (EC 3.2.1.31); heparanase (EC 3.2.1.166); β-4-*O*-methyl-glucuronidase (EC 3.2.1.-) + Feruloyl esterase (EC 3.1.1.73)

**198**	NEMBOFW57_007118	x						GH92(259-766)	GH92(9)	GH92	GH92				3.2.1.-	α-(1,2)-Mannosidase (EC 3.2.1.-); α-(1,3)-mannosidase (EC 3.2.1.-); α-(1,4)-mannosidase (EC 3.2.1.-); mannosyl-1-phosphodiester α-1,P-mannosidase (EC 3.2.1.-)
**199**	NEMBOFW57_007618	x						GH93(130-307)	GH93(1)	GH93	GH93		3.2.1.-	3.2.1.-	3.2.1.-	Exo-α-L-(1,5)-arabinanase (EC 3.2.1.-)
**200**	NEMBOFW57_007691	x	x					GH93(46-356)	GH93(1)	GH93	GH93			3.2.1.-	3.2.1.-	Exo-α-L-(1,5)-arabinanase (EC 3.2.1.-)
**201**	NEMBOFW57_006721	x						GH95(56-486)	GH95(1)	GH95	GH95					α-L-Fucosidase (EC 3.2.1.51); α-(1,2)-L-fucosidase (EC 3.2.1.63); α-L-galactosidase (EC 3.2.1.-)
**202**	NEMBOFW57_004027	x						GH114(33-255)	-	GH114	GH114			3.2.1.109	3.2.1.109	Endo-α-(1,4)-polygalactosaminidase (EC 3.2.1.109)
**203**	NEMBOFW57_005380	x	x					GH115(47-928)	GH115(1)	GH115	GH115			3.2.1.131	3.2.1.131	Xylan α-(1,2)-glucuronidase (3.2.1.131)
**204**	NEMBOFW57_004078	x	x					GH115(74-736)	GH115(1)	GH115	GH115			3.2.1.131	3.2.1.131	Xylan α-(1,2)-glucuronidase (3.2.1.131)
**205**	NEMBOFW57_009130	x	x					GH125(73-514)	GH125(2)	GH125	GH125				3.2.1.-	Exo-α-(1,6)-mannosidase (EC 3.2.1.-)

**206**	NEMBOFW57_004390				x			GH128(213-411)	GH128(5)	GH128	GH128			3.2.1.-|3.2.1.39	3.2.1.-|3.2.1.39	β-(1,3)-Glucanase (EC 3.2.1.39); β-(1,3)-glucosidase (EC 3.2.1.-)
**207**	NEMBOFW57_000683	x	x					GH131(18-257)	GH131(1)+CBM1(21)	GH131	GH131+CBM1			3.2.1.-	3.2.1.-	Broad specificity exo-β-(1,3)/(1,6)-glucanase with endo-β-(1,4)-glucanase activity (EC 3.2.1.-) + these modules cellulose binding function has been demonstrated
**208**	NEMBOFW57_004659	x	x					GH131(21-109)+GH131(108-232)	GH131(1)+CBM1(21)	CBM1+GH131+3.2.1.-	GH131+CBM1		3.2.1.-	3.1.1.-|3.2.1.-|3.2.1.176|3.2.1.4|3.2.1.55|3.2.1.8	3.1.1.-|3.1.1.6|3.1.1.72|3.2.1.-|3.2.1.132|3.2.1.14|3.2.1.151|3.2.1.176|3.2.1.4|3.2.1.55|3.2.1.78|3.2.1.8|3.2.1.91	Broad specificity exo-β-(1,3)/(1,6)-glucanase with endo-β-(1,4)-glucanase activity (EC 3.2.1.-) + these modules cellulose binding function has been demonstrated
**209**	NEMBOFW57_002736	x	x					GH131(35-308)	GH131(2)	GH131	GH131+CBM1					Broad specificity exo-β-(1,3)/(1,6)-glucanase with endo-β-(1,4)-glucanase activity (EC 3.2.1.-) + these modules cellulose binding function has been demonstrated
**210**	NEMBOFW57_002244	x		x	x	x	x	GH132(113-415)	GH132(3)	GH132	GH132			3.2.1.-	3.2.1.-	Activity on β-(1,3)-glucan (curdlan) shown
**211**	NEMBOFW57_007260				x			GH132(59-316)	GH132(2)	GH132	GH132				3.2.1.-	Activity on β-(1,3)-glucan (curdlan) shown
**212**	NEMBOFW57_001379	x						GH146(41-561)	GH146(2)+CBM13(17)	CBM13+GH146	GH146+CBM13					β-L-Arabinofuranosidase (EC 3.2.1.185) + CBM13 have been found in a number of other proteins of various functions

																including glycoside hydrolases and glycosyltransferases
213	NEMBOFW57_004244		x		x			GT20(9-477)	GT20(2)+CBM20(39)	GT20	GT20			2.4.1.15	2.4.1.15	α,α-Trehalose-phosphate synthase [UDP-forming] (EC 2.4.1.15)
214	NEMBOFW57_006478		x		x			GT35(163-878)	GT35(1)	GT35	GT35				2.4.1.1	Glycogen or starch phosphorylase (EC 2.4.1.1)
215	NEMBOFW57_003918				x			GT48(870-1650)	GT48(1)	GT48	GT48			2.4.1.34	2.4.1.34	β-(1,3)-Glucan synthase (EC 2.4.1.34)
216	NEMBOFW57_004511		x					GT90(266-523)+AA9(530-713)	AA9(9)+GT90(8)	GT90	GT90+AA9				2.4.2.-	UDP-Xyl: (mannosyl) glucuronoxylomannan/galactoxylomannan β-(1,2)-xylosyltransferase (EC 2.4.2.-); UDP-Glc: protein *O*-β-glucosyltransferase (EC 2.4.1.-); UDP-Xyl: protein *O*-β-xylosyltransferase (EC 2.4.2.-) + Lytic cellulose monooxygenase (C1-hydroxylating) (EC 1.14.99.54); lytic cellulose monooxygenase (C4-dehydrogenating) (EC 1.14.99.56)
217	NEMBOFW57_008589	x						PL1_4(111-280)	PL1(13)	PL1_4	PL1_4		4.2.2.10	4.2.2.10	4.2.2.10|4.2.2.2	Pectin lyase (EC 4.2.2.10)
218	NEMBOFW57_007080	x						PL1_7(42-144)	-	-	PL1_7			4.2.2.2	4.2.2.10|4.2.2.2|4.2.2.9	Pectate lyase (EC 4.2.2.2)

**219**	NEMBOFW57_010773	x	x					PL1_7(61-243)	PL1(16)	PL1_7	PL1_7			4.2.2.2	4.2.2.2|4.2.2.9	Pectate lyase (EC 4.2.2.2)
**220**	NEMBOFW57_009693	x	x					PL1_10(81-254)	PL1(47)	PL1_10	PL1_10			4.2.2.2	4.2.2.2|4.2.2.9	Pectate lyase (EC 4.2.2.2)
**221**	NEMBOFW57_009003	x	x					PL3_2(116-305)	PL3(10)	PL3_2	PL3_2		4.2.2.2	4.2.2.2	4.2.2.2	Pectate lyase (EC 4.2.2.2)
**222**	NEMBOFW57_006439	x						PL4_3(17-626)	PL4(6)	PL4_3	PL4_3			4.2.2.23	4.2.2.23	Rhamnogalacturonan endolyase (EC 4.2.2.23)
**223**	NEMBOFW57_009327	x	x					PL4_3(33-685)	PL4(6)	PL4_3	PL4_3			4.2.2.23	4.2.2.23	Rhamnogalacturonan endolyase (EC 4.2.2.23)
**224**	NEMBOFW57_004480	x	x					PL26(29-914)	PL26(1)	PL26	PL26		4.2.2.24	4.2.2.24	4.2.2.24	Rhamnogalacturonan exolyase (EC 4.2.2.24)

Supplementary Table S3: Functional prediction of the CAZymes found on maize leaves (MZ) or sugarcane bagasse (SCB) after 7, 14, 21 and 28 days. The proteins were subjected to in-solution tryptic digestion before LC-MS/MS analysis. For the EC classification, a PBLAST search was performed and the top 1, 10, 100 and 99999 EC classifications were fetched for the hits. For ambiguous or unknown CAZyme family prediction results, BLASTP annotations were used to match the CAZyme family.

#	**Accession number**	**SB**				**MZ**				**Family**	**top_1**	**top_10**	**top_100**	**top_99999**	**Non-Blast**	**Function**
		**7**	**14**	**21**	**28**	**7**	**14**	**21**	**28**							
1	NEMBOFW57_001352	X	X	X	X		X	X	X	AA3				1.1.3.13|1.1.3.16|1.1.3.7		Aryl alcohol oxidase (EC 1.1.3.7); alcohol oxidase (EC 1.1.3.13)

2	NEMBOFW57_008032	X								AA3_2				1.1.3.13|1.1.3.16|1.1.3.4|1.1.3.7		Aryl alcohol oxidase (EC 1.1.3.7); alcohol oxidase (EC 1.1.3.13)
3	NEMBOFW57_008083								X	AA3				1.1.3.4|1.1.3.7		Glucose 1-oxidase (EC 1.1.3.4); aryl alcohol oxidase (EC 1.1.3.7)
4	NEMBOFW57_009225								X	AA3_2			1.1.3.4	1.1.3.13|1.1.3.4|1.1.3.7		Glucose 1-oxidase (EC 1.1.3.4); aryl alcohol oxidase (EC 1.1.3.7); alcohol oxidase (EC 1.1.3.13)
5	NEMBOFW57_010289				X				X	AA3				1.1.3.13|1.1.3.7		Aryl alcohol oxidase (EC 1.1.3.7); alcohol oxidase (EC 1.1.3.13)
6	NEMBOFW57_010319	X	X	X	X	X	X	X	X	AA3				1.1.3.13|1.1.3.16|1.1.3.7		Aryl alcohol oxidase (EC 1.1.3.7); alcohol oxidase (EC 1.1.3.13)
7	NEMBOFW57_010449							X	X	AA3				1.1.3.13|1.1.3.16|1.1.3.4|1.1.3.7		Glucose 1-oxidase (EC 1.1.3.4); aryl alcohol oxidase (EC 1.1.3.7); alcohol oxidase (EC 1.1.3.13)
8	NEMBOFW57_010602	X	X					X	X	AA3				1.1.3.13|1.1.3.4|1.1.3.7		Glucose 1-oxidase (EC 1.1.3.4); aryl alcohol oxidase (EC 1.1.3.7); alcohol oxidase (EC 1.1.3.13)
9	NEMBOFW57_008624	X	X	X	X		X	X	X	AA3+AA8				1.1.3.13|1.1.3.7		Aryl alcohol oxidase (EC 1.1.3.7); alcohol oxidase (EC 1.1.3.13) + Iron reductase domain
10	NEMBOFW57_010246	X	X	X	X			X	X	AA3+AA8				1.1.3.13|1.1.3.16|1.1.3.7		Aryl alcohol oxidase (EC 1.1.3.7); alcohol oxidase (EC 1.1.3.13) + Iron reductase domain

11	NEMBOFW57_001011	X	X	X	X					AA3+AA8+CBM1				1.-.-.-|1.1.3.13|1.1.3.7|3.2.1.78|3.2.1.8		Alcohol oxidase (EC 1.1.3.13) + Iron reductase domain + these modules cellulose binding function has been demonstrated
12	NEMBOFW57_003104	X	X	X	X		X	X	X	AA5_1				1.1.3.47|1.1.3.7|1.1.3.9|1.2.3.15|2.4.2.26|3.2.1.58		Galactose oxidase (EC 1.1.3.9); glyoxal oxidase (EC 1.2.3.15)
13	NEMBOFW57_001116								X	AA7			1.1.3.-|3.2.1.58	1.1.3.-|3.2.1.58		Glucooligosaccharide oxidase (EC 1.1.3.-); chitooligosaccharide oxidase (EC 1.1.3.-)
14	NEMBOFW57_001310	X	X	X	X	X	X	X	X	AA7			1.1.3.-	1.1.3.-		Glucooligosaccharide oxidase (EC 1.1.3.-); chitooligosaccharide oxidase (EC 1.1.3.-)
15	NEMBOFW57_004115	X							X	AA7		1.1.3.-	1.1.3.-	1.1.3.-		Glucooligosaccharide oxidase (EC 1.1.3.-); chitooligosaccharide oxidase (EC 1.1.3.-)
16	NEMBOFW57_005776	X								AA7			1.1.3.-	1.1.3.-		Glucooligosaccharide oxidase (EC 1.1.3.-); chitooligosaccharide oxidase (EC 1.1.3.-)
17	NEMBOFW57_008720	X	X	X	X					AA8				1.-.-.-		Oxidoreductase (EC 1.-.-.-)
18	NEMBOFW57_001172	X								AA9					AA9 HMMER, Hotpep,	Lytic cellulose monooxygenase (C1-hydroxylating) (EC 1.14.99.54); lytic cellulose monooxygenase

															DIA- MOND, PBLAST	(C4-dehydrogenating) (EC 1.14.99.56)
19	NEMBOF W57_001 968						X			AA9					AA9 HMMER, Hotpep, DIA- MOND, PBLAST	Lytic cellulose monooxygenase (C1-hydroxylating) (EC 1.14.99.54); lytic cellulose monooxygenase (C4-dehydrogenating) (EC 1.14.99.56)
20	NEMBOF W57_004 556	X								AA9					AA9 HMMER, Hotpep, DIA- MOND, PBLAST	Lytic cellulose monooxygenase (C1-hydroxylating) (EC 1.14.99.54); lytic cellulose monooxygenase (C4-dehydrogenating) (EC 1.14.99.56)
21	NEMBOF W57_005 018	X								AA9					AA9 HMMER, Hotpep, DIA- MOND, PBLAST	Lytic cellulose monooxygenase (C1-hydroxylating) (EC 1.14.99.54); lytic cellulose monooxygenase (C4-dehydrogenating) (EC 1.14.99.56)
22	NEMBOF W57_007 876	X	X							AA9					AA9 HMMER, Hotpep, DIA- MOND, PBLAST	Lytic cellulose monooxygenase (C1-hydroxylating) (EC 1.14.99.54); lytic cellulose monooxygenase (C4-dehydrogenating) (EC 1.14.99.56)

23	NEMBOFW57_001044	X	X	X	X	X		X	X	AA9+CBM1					AA9 HMMER, Hotpep, DIAMOND	Lytic cellulose monooxygenase (C1-hydroxylating) (EC 1.14.99.54); lytic cellulose monooxygenase (C4-dehydrogenating) (EC 1.14.99.56) + these modules cellulose binding function has been demonstrated
24	NEMBOFW57_001466	X	X	X	X		X	X	X	AA9+CBM1					AA9 HMMER, Hotpep, DIAMOND, PBLAST	Lytic cellulose monooxygenase (C1-hydroxylating) (EC 1.14.99.54); lytic cellulose monooxygenase (C4-dehydrogenating) (EC 1.14.99.56) + these modules cellulose binding function has been demonstrated
25	NEMBOFW57_003955	X								AA12			1.-.-.-	1.-.-.-		Oxidoreductase (EC 1.-.-.-)
26	NEMBOFW57_003584				X					CBM1			3.1.1.-|3.2.1.-|3.2.1.176|3.2.1.4|3.2.1.8	3.1.1.-|3.1.1.6|3.1.1.72|3.2.1.-|3.2.1.14|3.2.1.151|3.2.1.176|3.2.1.4|3.2.1.55|3.2.1.78|3.2.1.8|3.2.1.91|4.2.2.10		These modules cellulose binding function has been demonstrated

27	NEMBOFW57_000190	X								CBM52			3.2.1.39	3.2.1.39		Binding to β-(1,3)-glucan demonstrated
28	NEMBOFW57_000278	X								CE1				2.3.1.122|2.3.1.20|3.1.1.-		Cinnamoyl esterase (EC 3.1.1.-); carboxylesterase (EC 3.1.1.1); diacylglycerol *O*-acyltransferase (EC 2.3.1.20); trehalose 6-*O*-mycolyltransferase (EC 2.3.1.122)
29	NEMBOFW57_002054	X								CE1		3.1.1.73	3.1.1.-|3.1.1.72|3.1.1.73	3.1.1.-|3.1.1.6|3.1.1.72|3.1.1.73		Feruloyl esterase (EC 3.1.1.73)
30	NEMBOFW57_004513	X								CE1		3.1.1.73	3.1.1.-|3.1.1.72|3.1.1.73	3.1.1.-|3.1.1.6|3.1.1.72|3.1.1.73		Feruloyl esterase (EC 3.1.1.73)
31	NEMBOFW57_007800	X								CE1		3.1.1.72	3.1.1.-|3.1.1.72|3.1.1.73	3.1.1.-|3.1.1.6|3.1.1.72|3.1.1.73		Acetyl xylan esterase (EC 3.1.1.72)
32	NEMBOFW57_007038	X						X	X	CE2			3.1.1.-|3.1.1.72|3.2.1.4	3.1.1.-|3.1.1.72|3.2.1.4		Acetyl xylan esterase (EC 3.1.1.72)
33	NEMBOFW57_009085	X	X	X	X					CE3			3.1.1.6	3.1.1.6|3.1.1.72		Acetylesterase (EC 3.1.1.6) active on various carbohydrate acetyl esters

34	NEMBOFW57_005718		X						X	CE4			3.1.1.72|3.5.1.41	3.1.1.72|3.2.1.8|3.5.1.41		Acetyl xylan esterase (EC 3.1.1.72); chitin deacetylase (EC 3.5.1.41)
35	NEMBOFW57_009578	X	X	X	X		X		X	CE4			3.1.1.72|3.5.1.41	3.1.1.72|3.5.1.41		Acetyl xylan esterase (EC 3.1.1.72); chitin deacetylase (EC 3.5.1.41)
36	NEMBOFW57_000575	X		X			X		X	CE4+CBM18				3.1.1.72|3.5.1.104|3.5.1.41		Acetyl xylan esterase (EC 3.1.1.72); chitin deacetylase (EC 3.5.1.41); chitooligosaccharide deacetylase (EC 3.5.1.-) + these modules chitin-binding function has been demonstrated
37	NEMBOFW57_005577				X					CE5				3.1.1.3|3.1.1.74		Cutinase (EC 3.1.1.74)
38	NEMBOFW57_007936								X	CE5		3.1.1.72	3.1.1.72	3.1.1.72		Acetyl xylan esterase (EC 3.1.1.72)
39	NEMBOFW57_010955	X								CE5		3.1.1.72	3.1.1.72	3.1.1.72|3.2.1.73|3.2.1.78|3.2.1.8		Acetyl xylan esterase (EC 3.1.1.72)
40	NEMBOFW57_006728	X	X	X	X		X	X	X	CE8		3.1.1.11	3.1.1.11	3.1.1.11		Pectin methylesterase (EC 3.1.1.11)
41	NEMBOFW57_000887	X	X	X	X		X	X	X	CE10					CE10 HMMER, PBLAST	acetylcholinesterase (EC 3.1.1.7)

42	NEMBOFW57_001299			X						CE10			3.1.1.6|3.1.1.73	3.1.1.6|3.1.1.73		Feruloyl esterase (EC 3.1.1.73); Acetylesterase (EC 3.1.1.6)
43	NEMBOFW57_000286						X			CE12				3.1.1.72		Acetyl xylan esterase (EC 3.1.1.72)
44	NEMBOFW57_001439	X	X	X	X		X	X	X	CE12			3.1.1.-	3.1.1.-		Pectin acetylesterase (EC 3.1.1.-); rhamnogalacturonan acetylesterase (EC 3.1.1.-)
45	NEMBOFW57_003741	X								CE15		3.1.1.-	3.1.1.-|3.1.1.72	3.1.1.-|3.1.1.72|3.2.1.40		Pectin acetylesterase (EC 3.1.1.-); rhamnogalacturonan acetylesterase (EC 3.1.1.-)
46	NEMBOFW57_004062	X	X	X	X		X	X	X	CE15		3.1.1.-	3.1.1.-	3.1.1.-|3.1.1.72|3.2.1.39|3.2.1.40		Pectin acetylesterase (EC 3.1.1.-); rhamnogalacturonan acetylesterase (EC 3.1.1.-)
47	NEMBOFW57_006599								X	CE16		3.1.1.6	3.1.1.6	3.1.1.6|3.2.1.51		Acetylesterase (EC 3.1.1.6) active on various carbohydrate acetyl esters
48	NEMBOFW57_000322	X	X	X						GH1	3.2.1.21	3.2.1.21	3.2.1.21	2.4.1.-|3.2.1.117|3.2.1.118|3.2.1.119|3.2.1.125|3.2.1.149|3.2.1.161|3.2.1.21|3.2.1.25|3.2.1.38|3.2.1.74		β-Glucosidase (EC 3.2.1.21)

49	NEMBOFW57_004374	X	X	X	X		X	X	X	GH2			3.2.1.23	3.2.1.23		β-Galactosidase (EC 3.2.1.23)
50	NEMBOFW57_009152	X	X	X	X		X	X	X	GH2			3.2.1.23	3.2.1.23		β-Galactosidase (EC 3.2.1.23)
51	NEMBOFW57_002850			X	X					GH2+CBM32+CBM51+CBM67			3.2.1.23	3.2.1.23		β-Galactosidase (EC 3.2.1.23) + CBM32 binding to polygalacturonic acid, CBM51 Bbnding to galactose, CBM67 L-rhamnose binding activity has been demonstrated.
52	NEMBOFW57_002037	X	X		X			X	X	GH2+CBM42+CBM67				3.2.1.146		β-D-Galactofuranosidase (EC 3.2.1.146) + CBM42 binding to arabinofuranose, CBM67 L-rhamnose binding activity has been demonstrated.
53	NEMBOFW57_002874	X	X	X	X			X	X	GH3	3.2.1.37	3.2.1.37	3.2.1.37|3.2.1.55|3.2.1.8	3.2.1.-|3.2.1.21|3.2.1.37|3.2.1.55|3.2.1.8		Xylan β-(1,4)-xylosidase (EC 3.2.1.37)
54	NEMBOFW57_002909	X	X					X	X	GH3		3.2.1.37	3.2.1.37|3.2.1.55	3.2.1.-|3.2.1.37|3.2.1.55|3.2.1.8		Xylan β-(1,4)-xylosidase (EC 3.2.1.37)
55	NEMBOFW57_005247		X							GH3			3.2.1.21	3.2.1.-|3.2.1.21|3.2.1.37		β-Glucosidase (EC 3.2.1.21)

56	NEMBOFW57_008747	X								GH3			3.2.1.21	3.2.1.-|3.2.1.21|3.2.1.37		β-Glucosidase (EC 3.2.1.21)
57	NEMBOFW57_009222								X	GH3		3.2.1.21	3.2.1.21|3.2.1.37	3.2.1.21|3.2.1.37|3.2.1.55		β-Glucosidase (EC 3.2.1.21)
58	NEMBOFW57_004293	X	X							GH5_5+CBM1		3.2.1.4	3.2.1.4	3.2.1.4		Endoglucanase (EC 3.2.1.4) + these modules cellulose binding function has been demonstrated
59	NEMBOFW57_009419				X				X	GH5_16		3.2.1.164	3.2.1.164	3.2.1.164|3.2.1.4		Endo-β-(1,6)-galactanase (EC 3.2.1.164)
60	NEMBOFW57_001193	X								GH5_23		3.2.1.-|3.2.1.149	3.2.1.-|3.2.1.149|3.2.1.55|3.2.1.58|3.2.1.75	3.2.1.-|3.2.1.149|3.2.1.21|3.2.1.55|3.2.1.58|3.2.1.75		β-Primeverosidase (EC 3.2.1.149); β-(1,3)-mannanase (EC 3.2.1.-); arabinoxylan-specific endo-(β-1,4)-xylanase (EC 3.2.1.-) β-glycosidase (EC 3.2.1.-); β-rutinosidase /α-L-rhamnose-(1,6)-β-D-glucosidase (EC 3.2.1.-); glucomannan-specific endo-Î²-(1,4)-glucanase (EC 3.2.1.-)
61	NEMBOFW57_008641	X	X	X	X		X	X	X	GH6		3.2.1.4|3.2.1.91	3.2.1.4|3.2.1.91	3.2.1.-|3.2.1.4|3.2.1.8|3.2.1.91		Endoglucanase (EC 3.2.1.4); cellobiohydrolase (EC 3.2.1.91)
62	NEMBOFW57_003080	X						X	X	GH7		3.2.1.176	3.2.1.132|3.2.1.176	3.2.1.132|3.2.1.176|3.2.1.4|3.2.1.73		Reducing end-acting cellobiohydrolase (EC 3.2.1.176)

63	NEMBOFW57_008456	X								GH7		3.2.1.176	3.2.1.176	3.2.1.132|3.2.1.176|3.2.1.4|3.2.1.73		Reducing end-acting cellobiohydrolase (EC 3.2.1.176)
64	NEMBOFW57_009077		X	X	X			X	X	GH7		3.2.1.4	3.2.1.176|3.2.1.4|3.2.1.73	3.2.1.132|3.2.1.176|3.2.1.4|3.2.1.73		Endo-β-(1,4)-glucanase (EC 3.2.1.4)
65	NEMBOFW57_010165				X					GH7		3.2.1.4|3.2.1.73	3.2.1.176|3.2.1.4|3.2.1.73	3.2.1.132|3.2.1.176|3.2.1.4|3.2.1.73		Endo-β-(1,4)-glucanase (EC 3.2.1.4); endo-β-(1,3)-(1,4)-glucanase (EC 3.2.1.73)
66	NEMBOFW57_010290	X	X	X	X			X	X	GH7		3.2.1.176	3.2.1.132|3.2.1.176	3.2.1.132|3.2.1.176		Reducing end-acting cellobiohydrolase (EC 3.2.1.176)
67	NEMBOFW57_010291	X	X	X	X	X	X	X	X	GH7		3.2.1.176	3.2.1.176	3.2.1.132|3.2.1.176|3.2.1.4|3.2.1.73		Reducing end-acting cellobiohydrolase (EC 3.2.1.176)
68	NEMBOFW57_007704	X	X	X	X					GH7+CBM1	3.2.1.176	3.2.1.176	3.2.1.132|3.2.1.176	3.2.1.132|3.2.1.176|3.2.1.4		Reducing end-acting cellobiohydrolase (EC 3.2.1.176) + these modules cellulose binding function has been demonstrated
69	NEMBOFW57_001316				X					GH10		3.2.1.8	3.2.1.8	3.2.1.8		Endo-(1,4)-β-xylanase (EC 3.2.1.8)
70	NEMBOFW57_004303	X	X	X	X				X	GH10		3.2.1.8	3.2.1.8	3.1.1.73|3.2.1.8		Endo-(1,4)-β-xylanase (EC 3.2.1.8)
71	NEMBOFW57_006022	X								GH10		3.2.1.8	3.2.1.8	3.1.1.73|3.2.1.8		Endo-(1,4)-β-xylanase (EC 3.2.1.8)

72	NEMBOFW57_008581	X					X	X	X	GH10		3.2.1.8	3.2.1.8	3.2.1.8		Endo-(1,4)-β-xylanase (EC 3.2.1.8)
73	NEMBOFW57_008598	X	X	X	X		X	X	X	GH10		3.2.1.8	3.2.1.8	3.1.1.73|3.2.1.8		Endo-(1,4)-β-xylanase (EC 3.2.1.8)
74	NEMBOFW57_010962	X								GH10		3.2.1.8	3.2.1.8	3.1.1.73|3.2.1.8		Endo-(1,4)-β-xylanase (EC 3.2.1.8)
75	NEMBOFW57_003635	X		X						GH10+CBM1	3.2.1.8	3.2.1.8	3.2.1.8	3.1.1.73|3.2.1.8		Endo-(1,4)-β-xylanase (EC 3.2.1.8) + these modules cellulose binding function has been demonstrated
76	NEMBOFW57_001184							X	X	GH12		3.2.1.4	3.2.1.151|3.2.1.4|3.2.1.73	3.2.1.151|3.2.1.4|3.2.1.73		Endoglucanase (EC 3.2.1.4)
77	NEMBOFW57_007690		X	X						GH12+CE1			3.2.1.4	3.1.1.72|3.1.1.73|3.2.1.151|3.2.1.4|3.2.1.73		Endoglucanase (EC 3.2.1.4) + acetyl xylan esterase (EC 3.1.1.72); feruloyl esterase (EC 3.1.1.73)
78	NEMBOFW57_002472	X								GH15					GH15 HMMER, Hotpep, DIAMOND, PBLAST	α,α-Trehalase (EC 3.2.1.28)
79	NEMBOFW57_007659	X						X	X	GH15+CBM20	3.2.1.3	3.2.1.3	3.2.1.3	3.2.1.3		Glucoamylase (EC 3.2.1.3) + these modules starch-binding binding function has been demonstrated

80	NEMBOFW57_009932		X							GH15+CBM20	3.2.1.3	3.2.1.3	3.2.1.3	3.2.1.1|3.2.1.3		Glucoamylase (EC 3.2.1.3)
81	NEMBOFW57_003172								X	GH16				2.4.1.-|3.2.1.-|3.2.1.39		Endo-β-(1,4)-galactosidase (EC 3.2.1.-); chitin-β-(1,6)-glucanosyltransferase (EC 2.4.1.-); β-transglycosidase (EC 2.4.1.-); β-glycosidase (EC 3.2.1.-); β-carrageenase (EC 3.2.1.-); endo-β-(1,3)-glucanase / laminarinase (EC 3.2.1.39)
82	NEMBOFW57_004987				X					GH16		3.2.1.73	2.4.1.-|3.2.1.-|3.2.1.39|3.2.1.6|3.2.1.73	2.4.1.-|3.2.1.-|3.2.1.1|3.2.1.35|3.2.1.39|3.2.1.41|3.2.1.6|3.2.1.73		Licheninase (EC 3.2.1.73)
83	NEMBOFW57_008500	X		X	X		X	X	X	GH16			2.4.1.-|3.2.1.39	2.4.1.-|3.2.1.-|3.2.1.39		Chitin-β-(1,6)-glucanosyltransferase (EC 2.4.1.-); β-transglycosidase (EC 2.4.1.-); endo-β-(1,3)-glucanase / laminarinase (EC 3.2.1.39)
84	NEMBOFW57_000084							X	X	GH16+CBM18			2.4.1.-|3.2.1.-	2.4.1.-|3.2.1.-|3.2.1.39		Endo-β-(1,4)-galactosidase (EC 3.2.1.-); chitin-β-(1,6)-glucanosyltransferase (EC 2.4.1.-); β-transglycosidase (EC 2.4.1.-); β-glycosidase (EC 3.2.1.-); β-

															carrageenase (EC 3.2.1.-) + these modules chitin binding function has been demonstrated
85	NEMBOFW57_000164	X	X	X	X	X	X	X	X	GH17				2.4.1.-|3.2.1.-		β-1,3-Glucanosyltransglycosylase (EC 2.4.1.-); β-1,3-glucosidase (EC 3.2.1.-)
86	NEMBOFW57_002998	X								GH18			3.2.1.14	3.2.1.14		Chitinase (EC 3.2.1.14)
87	NEMBOFW57_001287							X		GH18+CBM18		3.2.1.14	3.2.1.14	3.2.1.-|3.2.1.14		Chitinase (EC 3.2.1.14) + these modules chitin-binding function has been demonstrated
88	NEMBOFW57_006926		X	X	X		X	X	X	GH18+CBM18+CBM50				3.2.1.14		Chitinase (EC 3.2.1.14) + CBM18 chitin binding, CBM50 chito-pentaose binding function has been demonstrated
89	NEMBOFW57_003169	X	X	X	X					GH20			3.2.1.52	3.2.1.52		β-Hexosaminidase (EC 3.2.1.52)
90	NEMBOFW57_004532								X	GH24				3.2.1.17		Lysozyme (EC 3.2.1.17)
91	NEMBOFW57_003773								X	GH27			3.2.1.22	3.2.1.22|3.2.1.88		α-Galactosidase (EC 3.2.1.22)
92	NEMBOFW57_008762							X		GH27	3.2.1.22	3.2.1.22	3.2.1.22	2.4.1.-|3.2.1.22		α-Galactosidase (EC 3.2.1.22)

93	NEMBOFW57_004007						X	X	X	GH28		3.2.1.67	3.2.1.67	3.2.1.-|3.2.1.15|3.2.1.67		Exo-polygalacturonase (EC 3.2.1.67)
94	NEMBOFW57_009223	X								GH31					GH31 HMMER, Hotpep, DIAMOND, PBLAST	α-Xylosidase (EC 3.2.1.177)
95	NEMBOFW57_001288	X	X		X		X	X	X	GH35				3.2.1.23		β-Galactosidase (EC 3.2.1.23)
96	NEMBOFW57_001853	X	X					X	X	GH35		3.2.1.23	2.4.1.-|3.2.1.-|3.2.1.23	2.4.1.-|3.2.1.-|3.2.1.23		β-Galactosidase (EC 3.2.1.23)
97	NEMBOFW57_003451	X						X	X	GH37			3.2.1.28	3.2.1.28		α,α-Trehalase (EC 3.2.1.28)
98	NEMBOFW57_001526	X								GH43_1		3.2.1.37	3.2.1.37|3.2.1.55	3.2.1.37|3.2.1.55		β-Xylosidase (EC 3.2.1.37)
99	NEMBOFW57_007945	X	X							GH43_11		3.2.1.37|3.2.1.55	3.2.1.37|3.2.1.55	3.2.1.37|3.2.1.55		β-Xylosidase (EC 3.2.1.37); α-L-Arabinofuranosidase (EC 3.2.1.55)
100	NEMBOFW57_003714	X	X	X	X		X	X	X	GH43_14			3.2.1.37	3.2.1.37|3.2.1.55		β-Xylosidase (EC 3.2.1.37)

101	NEMBOFW57_008512	X	X				X	X	X	GH43_14			3.2.1.37	3.2.1.37|3.2.1.55		β-Xylosidase (EC 3.2.1.37)
102	NEMBOFW57_001268						X	X	X	GH43_26+CBM42		3.2.1.55	3.2.1.-|3.2.1.55	3.2.1.-|3.2.1.55		α-L-Arabinofuranosidase (EC 3.2.1.55) + Binding to arabinofuranose (present in arabinoxylan) has been demonstrated.
103	NEMBOFW57_000134	X					X	X	X	GH43_36		3.2.1.55	3.2.1.55	3.2.1.37|3.2.1.55		α-L-Arabinofuranosidase (EC 3.2.1.55)
104	NEMBOFW57_009841	X							X	GH43_36				3.2.1.37|3.2.1.55		β-Xylosidase (EC 3.2.1.37); α-L-Arabinofuranosidase (EC 3.2.1.55)
105	NEMBOFW57_002500	X	X	X	X			X	X	GH45		3.2.1.4	3.2.1.4	3.2.1.151|3.2.1.4|3.2.1.78		Endoglucanase (EC 3.2.1.4)
106	NEMBOFW57_002223	X								GH53		3.2.1.89	3.2.1.89	3.2.1.89		Endo-β-1,4-galactanase (EC 3.2.1.89)
107	NEMBOFW57_000338	X						X	X	GH54+CBM13+CBM42		3.2.1.37|3.2.1.55	3.2.1.37|3.2.1.55	3.2.1.146|3.2.1.20|3.2.1.37|3.2.1.55		α-L-Arabinofuranosidase (EC 3.2.1.55); β-xylosidase (EC 3.2.1.37) + CBM42 binding to arabinofuranose has been demonstrated. CBM13 have been found in a number of other proteins of various functions including glycoside hydrolases and glycosyltransferases

108	NEMBOFW57_001114	X								GH55		3.2.1.58	3.2.1.58	3.2.1.39|3.2.1.58		Exo-β-(1,3)-glucanase (EC 3.2.1.58)
109	NEMBOFW57_008041	X	X	X	X		X	X	X	GH55			3.2.1.58	3.2.1.39|3.2.1.58		Exo-β-(1,3)-glucanase (EC 3.2.1.58)
110	NEMBOFW57_008270	X	X	X	X		X	X	X	GH55			3.2.1.39|3.2.1.58	3.2.1.39|3.2.1.58		Exo-β-(1,3)-glucanase (EC 3.2.1.58); endo-β-(1,3)-glucanase (EC 3.2.1.39)
111	NEMBOFW57_001269	X			X			X	X	GH62	3.2.1.55	3.2.1.55	3.2.1.55	3.1.1.72|3.1.1.73|3.2.1.-|3.2.1.55|3.2.1.8		α-L-Arabinofuranosidase (EC 3.2.1.55)
112	NEMBOFW57_004236				X					GH67		3.2.1.131|3.2.1.139	3.2.1.131|3.2.1.139	3.2.1.-|3.2.1.131|3.2.1.139		α-Glucuronidase (EC 3.2.1.139); xylan α-(1,2)-glucuronidase (EC 3.2.1.131)
113	NEMBOFW57_005284			X					X	GH71					GH71 DIAMOND, PBLAST	α-(1,3)-Glucanase (EC 3.2.1.59)
114	NEMBOFW57_004454	X	X		X			X		GH72			2.4.1.-	2.4.1.-		β-(1,3)-Glucanosyltransglycosylase (EC 2.4.1.-)
115	NEMBOFW57_007083	X	X	X	X		X	X		GH72			2.4.1.-	2.4.1.-		β-(1,3)-Glucanosyltransglycosylase (EC 2.4.1.-)
116	NEMBOFW57_005755	X	X		X			X	X	GH72+CBM43			2.4.1.-	2.4.1.-		β-(1,3)-Glucanosyltransglycosylase (EC 2.4.1.-) + the β-(1,3)-glucan

																binding function has been demonstrated
117	NEMBOFW57_008527	X							X	GH74+CBM1		3.2.1.151	3.2.1.151	3.2.1.151		Xyloglucanase (EC 3.2.1.151) + these modules cellulose binding function has been demonstrated
118	NEMBOFW57_006928	X	X	X	X					GH75		3.2.1.132	3.2.1.132	3.2.1.132		Chitosanase (EC 3.2.1.132)
119	NEMBOFW57_004002	X					X	X	X	GH79			3.2.1.-|3.2.1.31	3.2.1.-|3.2.1.166|3.2.1.31		β-Glucuronidase (EC 3.2.1.31); β-4-*O*-methyl-glucuronidase (EC 3.2.1.-)
120	NEMBOFW57_004738		X	X	X				X	GH79+CE1			3.1.1.73	3.1.1.72|3.1.1.73|3.2.1.-|3.2.1.166|3.2.1.31		β-Glucuronidase (EC 3.2.1.31); heparanase (EC 3.2.1.166); β-4-*O*-methyl-glucuronidase (EC 3.2.1.-) + Feruloyl esterase (EC 3.1.1.73)
121	NEMBOFW57_007691	X	X	X	X		X	X	X	GH93			3.2.1.-	3.2.1.-		Exo-α-L-(1,5)-arabinanase (EC 3.2.1.-)
122	NEMBOFW57_006721			X			X		X	GH95					GH95 HMMER, Hotpep, DIAMOND, PBLAST	α-L-Fucosidase (EC 3.2.1.51); α-(1,2)-L-fucosidase (EC 3.2.1.63); α-L-galactosidase (EC 3.2.1.-)
123	NEMBOFW57_005380	X						X	X	GH115			3.2.1.131	3.2.1.131		Xylan α-(1,2)-glucuronidase (3.2.1.131)

124	NEMBOFW57_009130		X	X	X					GH125				3.2.1.-		Exo-α-(1,6)-mannosidase (EC 3.2.1.-)
125	NEMBOFW57_002736								X	GH131					GH131 HMMER, Hotpep, DIA-MOND, PBLAST	Broad specificity exo-β-(1,3)/(1,6)-glucanase with endo-β-(1,4)-glu-canase activity (EC 3.2.1.-)
126	NEMBOFW57_004659	X								GH131+CBM1		3.2.1.-	3.1.1.-|3.2.1.-|3.2.1.176|3.2.1.4|3.2.1.55|3.2.1.8	3.1.1.-|3.1.1.6|3.1.1.72|3.2.1.-|3.2.1.132|3.2.1.14|3.2.1.151|3.2.1.176|3.2.1.4|3.2.1.55|3.2.1.78|3.2.1.8|3.2.1.91		Broad specificity exo-β-(1,3)/(1,6)-glucanase with endo-β-(1,4)-glu-canase activity (EC 3.2.1.-) + these modules cellulose binding function has been demonstrated
127	NEMBOFW57_000683	X	X	X	X		X			GH131+CMB1			3.2.1.-	3.2.1.-		Broad specificity exo-β-(1,3)/(1,6)-glucanase with endo-β-(1,4)-glu-canase activity (EC 3.2.1.-) + these modules cellulose binding function has been demonstrated
128	NEMBOFW57_005402	X								GH154					GH154 HMMER, Hotpep, DIAMOND	β-Glucuronidase (3.2.1.31)

129	NEMBOFW57_009579	X								GT1			2.4.1.-	2.4.1.-		Ecdysteroid UDP-glucosyltransferase (EC 2.4.1.-); salicylic acid β-glucosyltransferase (EC 2.4.1.-); anthocyanin 3-*O*-galactosyltransferase (EC 2.4.1.-); anthocyanin 5-*O*-glucosyltransferase (EC 2.4.1.-); dTDP-β-2-deoxy-L-fucose: α-L-2-deoxyfucosyltransferase (EC 2.4.1.-); UDP-β-L-rhamnose: α-L-rhamnosyltransferase (EC 2.4.1.-); zeaxanthin glucosyltransferase (EC 2.4.1.-); UDP-Glc: flavone-6-*C*-glucosytransferase (EC 2.4.1.-); UDP-Glc: hydroxycinnamic acid *O*-β-glucosyltransferase (EC 2.4.1.-); UDP-Glc: cinnamoyl *O*-β-glucosyltransferase (EC 2.4.1.-); UDP-Arap: flavone-*C*-arabinosyltransferase (EC 2.4.1.-)
**130**	NEMBOFW57_010773		X	X	X		X	X	X	PL1_7			4.2.2.2	4.2.2.2|4.2.2.9		Pectate lyase (EC 4.2.2.2)
**131**	NEMBOFW57_009693		X	X	X			X	X	PL1_10			4.2.2.2	4.2.2.2|4.2.2.9		Pectate lyase (EC 4.2.2.2)

**132**	NEMBOFW57_009003	X		X						PL3_2		4.2.2.2	4.2.2.2	4.2.2.2		Pectate lyase (EC 4.2.2.2)
**133**	NEMBOFW57_009327						X	X	X	PL4_3			4.2.2.23	4.2.2.23		Rhamnogalacturonan endolyase (EC 4.2.2.23)
**134**	NEMBOFW57_004480								X	PL26		4.2.2.24	4.2.2.24	4.2.2.24		Rhamnogalacturonan exolyase (EC 4.2.2.24)

Supplementary Table S4. Chitinase activity of 28 fungal strains (Brandt et al. 2018).

Working Name	DSMZ ID	Unit	standard deviation	Unit/mL	standard deviation	Unit/mg	standard deviation
FW35	106735	0.0233	0.0011	0.1163	0.0053	0.1548	0.0066
Fsh101	107513	0.0104	0.0002	0.0519	0.0012	0.1306	0.0034
FF1	104516	0.0107	0.0048	0.0534	0.0238	0.1286	0.0646
FW57	105789	0.0160	0.0004	0.0800	0.0019	0.1111	0.0026
Fsh6	106727	0.0128	0.0004	0.0639	0.0018	0.0964	0.0028
SF7	106361	0.0139	0.0005	0.0696	0.0026	0.0839	0.0034
FL6	106243	0.0142	0.0001	0.0712	0.0007	0.0621	0.0007
FH4	106231	0.0097	0.0001	0.0487	0.0004	0.0611	0.0005
FH101	106237	0.0062	0.0001	0.0311	0.0003	0.0510	0.0006
Fsh201	107514	0.0089	0.0003	0.0447	0.0013	0.0476	0.0015
FH3	106232	0.0091	0.0002	0.0456	0.0011	0.0423	0.0010
FL10	106248	0.0090	0.0002	0.0452	0.0010	0.0420	0.0008
Fi22	106464	0.0087	0.0001	0.0434	0.0006	0.0411	0.0006
Fi39	106482	0.0072	0.0001	0.0359	0.0007	0.0403	0.0007
FW36	106736	0.0096	0.0004	0.0481	0.0018	0.0398	0.0015
Fi43	106485	0.0154	0.0008	0.0768	0.0042	0.0374	0.0015
FL18	106255	0.0081	0.0002	0.0405	0.0008	0.0372	0.0007
FL101	106261	0.0098	0.0001	0.0491	0.0007	0.0367	0.0005
Fi21	106463	0.0092	0.0001	0.0462	0.0007	0.0363	0.0005
SF19.1	106380	0.0071	0.0001	0.0354	0.0004	0.0344	0.0004
FL100	106490	0.0077	0.0001	0.0383	0.0006	0.0337	0.0004
FL2	106239	0.0056	0.0001	0.0281	0.0004	0.0323	0.0005
Fi34	106474	0.0067	0.0000	0.0337	0.0002	0.0310	0.0002
Fsh102	105790	0.0044	0.0001	0.0220	0.0003	0.0294	0.0004
Fsh200	107515	0.0049	0.0000	0.0245	0.0002	0.0293	0.0003
Fi10.2	106451	0.0016	0.0000	0.0082	0.0001	0.0289	0.0004

FW24	106709	0.0033	0.0000	0.0163	0.0001	0.0243	0.0001
Fi24	106466	0.0053	0.0001	0.0264	0.0004	0.0232	0.0003
FW63	106754	0.0003	0.0000	0.0016	0.0000	0.0018	0.0000

Supplementary Table S5. Proteins of the fungal isolate FW57 induced on different chitin-like substrates and biomass substrates (MZ and SCB). The proteins were separated by SDS-PAGE followed by in-gel tryptic digestion and LC-MS/MS analysis. The accession number, coverage (%), number of peptides (# peptides), peptide-to-spectrum matches (# PSMs), number of unique peptides (# Unique Peptides), molecular weight in kDa (MW [kDa]), calculated isoelectric point (calc. pI), Score Sequest HT and number of Peptides Sequest HT (# Peptides Sequest HT) were compared with the automated translation of the genome of the fungal isolate *Staphylotrichum longicolleum* DSM105789. Pfam annotation (query name) and Pfam accession code (query accession) were used for functional annotation. For ambiguous or unknown Pfam results, BLASTP annotations was used to match the cellular function.

Accession number	Coverage [%]	# Peptides	# PSMs	# Unique Peptides	MW [kDa]	calc. pI	Score Sequest HT: Sequest HT	# Peptides (by Search Engine): Sequest HT	MZ	SCB	CS			C	query name	query accession	BLAST	Function
											L	M	H					
NEMBOFW57_008641	54	17	63	17	42.2	6.27	157.47	17	x	x	x	x	x	x	Glyco_hydro_6	PF01341.18		CAZYME
NEMBOFW57_008624	50	27	56	27	82.0	6.28	136.2	27	x	x					CDH-cyt	PF16010.6		CAZYME
NEMBOFW57_010319	62	28	59	28	63.3	6.84	130.17	28	x	x	x	x	x	x	GMC_oxred_N	PF00732.20		CAZYME
NEMBOFW57_010291	91	13	57	13	17.3	5.3	126.24	13	x	x		x			Glyco_hydro_7	PF00840.21		CAZYME
NEMBOFW57_000164	52	16	32	16	41.7	5.35	120.16	16	x	x	x	x	x	x	Glyco_hydro_17	PF00332.19		CAZYME
NEMBOFW57_008581	74	15	63	15	35.4	8.46	118.41	15	x	x					Glyco_hydro_10	PF00331.21		CAZYME
NEMBOFW57_010290	33	9	69	9	35.2	7.83	112.6	9	x	x		x			Glyco_hydro_7	PF00840.21		CAZYME
NEMBOFW57_009932	25	11	28	11	70.5	6.55	109.17	11	x	x	x	x	x	x	Glyco_hydro_15	PF00723.22		CAZYME
NEMBOFW57_009152	41	31	65	31	93.9	7.43	108.74	31	x	x			x		Glyco_hydro2_C5	PF18565.2		CAZYME
NEMBOFW57_004062	62	16	48	16	41.4	8.41	108.47	16	x	x					DUF463	PF04317.13		CAZYME
NEMBOFW57_001288	48	23	48	23	64.8	7.61	100.04	23	x	x					Glyco_hydro_35	PF01301.20		CAZYME

NEMBOFW57_001051	21	14	30	14	114.5	5.05	97.56	14	x	x	x	x	x		Glyco_hydro_3_C	PF01915.23		CAZYME
NEMBOFW57_008598	65	19	53	16	39.7	7.44	95.76	19	x	x					Glyco_hydro_10	PF00331.21		CAZYME
NEMBOFW57_010289	40	21	40	21	71.2	6.7	92.35	21	x	x	x	x	x		GMC_oxred_N	PF00732.20		CAZYME
NEMBOFW57_006440	41	14	25	14	65.1	8.9	91.51	14	x	x	x	x	x	x	GMC_oxred_N	PF00732.20		CAZYME
NEMBOFW57_004303	70	19	42	16	36.3	7.2	90.29	19	x	x	x	x	x	x	Glyco_hydro_10	PF00331.21		CAZYME
NEMBOFW57_008041	20	9	23	9	100.9	5.92	88.14	9	x	x	x	x	x	x	Pectate_lyase_3	PF12708.8		CAZYME
NEMBOFW57_010246	37	17	34	17	58.2	7.17	81.86	17	x	x					GMC_oxred_N	PF00732.20		CAZYME
NEMBOFW57_008500	30	8	20	8	39.2	5.39	75.82	8	x	x	x	x	x	x	Glyco_hydro_16	PF00722.22		CAZYME
NEMBOFW57_001439	62	10	44	10	27.6	8.91	74.02	10	x	x					Lipase_GDSL_2	PF13472.7		CAZYME
NEMBOFW57_001316	41	11	31	11	41.7	6.76	73.41	11	x	x	x	x	x	x	Glyco_hydro_10	PF00331.21		CAZYME
NEMBOFW57_003169	45	17	36	17	64.9	5.91	72.28	17	x	x	x	x	x		Glyco_hydro_20	PF00728.23		CAZYME
NEMBOFW57_001044	39	10	39	10	32.9	7.18	72.21	10	x	x	x	x	x		AA9	PF03443.15		CAZYME
NEMBOFW57_001114	21	9	19	9	82.0	5.33	63.89	9	x	x	x	x	x	x	Pectate_lyase_3	PF12708.8		CAZYME
NEMBOFW57_005755	27	12	15	12	57.0	5.02	62.39	12	x	x	x	x	x	x	Glyco_hydro_72	PF03198.15		CAZYME
NEMBOFW57_004148	23	10	20	10	73.6	6.77	60.8	10			x	x	x	x	Glyco_hydro_32N	PF00251.21		CAZYME
NEMBOFW57_004374	28	24	30	24	97.2	9.0	59.76	24	x	x					Glyco_hydro2_C5	PF18565.2		CAZYME
NEMBOFW57_009691	14	21	29	21	201.2	8.25	59.64	21	x	x					Glyco_hydro_31	PF01055.27		CAZYME
NEMBOFW57_003451	37	18	31	18	77.8	5.94	59.26	18	x	x	x	x	x		Trehalase	PF01204.19		CAZYME
NEMBOFW57_009085	36	7	31	7	25.7	7.9	58.91	7	x	x	x	x			Lipase_GDSL_2	PF13472.7		CAZYME
NEMBOFW57_007704	43	19	36	18	56.9	5.78	58.79	19		x	x	x	x	x	Glyco_hydro_7	PF00840.21		CAZYME
NEMBOFW57_001352	46	21	27	21	67.4	8.38	57.9	21	x	x					GMC_oxred_N	PF00732.20		CAZYME
NEMBOFW57_003104	17	9	17	9	104.3	5.4	56.16	9	x	x	x	x	x	x	WSC	PF01822.20		CAZYME
NEMBOFW57_004454	21	8	16	8	51.3	5.4	55.27	8	x	x	x	x	x	x	Glyco_hydro_72	PF03198.15		CAZYME
NEMBOFW57_003714	51	19	30	19	51.3	7.52	53.86	19	x	x					Glyco_hydro_43	PF04616.15		CAZYME
NEMBOFW57_001310	38	15	24	15	51.2	7.8	53.7	15	x	x	x	x	x	x	FAD_binding_4	PF01565.24		CAZYME
NEMBOFW57_000084	21	5	16	5	47.3	5.5	53.22	5	x	x	x	x	x	x	Glyco_hydro_16	PF00722.22		CAZYME
NEMBOFW57_008561	23	10	16	10	54.0	6.89	51.95	10	x	x	x	x	x		FAD_binding_4	PF01565.24		CAZYME

NEMBOFW57_005380	37	26	31	26	103.3	7.42	51.09	26	x	x					Glyco_hydro_115	PF15979.6		CAZYME
NEMBOFW57_008270	36	12	26	12	55.3	7.81	50.57	12	x	x					Pectate_lyase_3	PF12708.8		CAZYME
NEMBOFW57_001466	52	14	28	14	33.3	7.65	49.33	14	x	x					AA9	PF03443.15		CAZYME
NEMBOFW57_002419	17	7	16	7	54.0	5.38	47.47	7			x	x	x	x	zf-Nse	PF11789.9		CAZYME
NEMBOFW57_001269	34	11	23	11	39.0	5.88	45.16	11	x	x					Glyco_hydro_62	PF03664.14		CAZYME
NEMBOFW57_010602	40	16	20	16	62.8	8.13	44.75	16	x	x	x	x	x	x	GMC_oxred_N	PF00732.20		CAZYME
NEMBOFW57_004078	24	19	20	19	121.5	7.18	43.97	19	x	x					Glyco_hydro_115	PF15979.6		CAZYME
NEMBOFW57_001011	25	16	20	16	87.8	8.12	43.79	16	x	x					CDH-cyt	PF16010.6		CAZYME
NEMBOFW57_007083	18	6	13	6	49.5	8.22	43.29	6	x	x	x	x	x	x	Glyco_hydro_72	PF03198.15		CAZYME
NEMBOFW57_006022	28	9	17	9	40.6	7.49	42.83	9	x	x					Glyco_hydro_10	PF00331.21		CAZYME
NEMBOFW57_009282	25	8	11	8	58.7	5.54	41.69	8	x		x	x	x	x	Alpha-amylase	PF00128.25		CAZYME
NEMBOFW57_004071	33	9	18	9	57.6	6.02	41.14	9	x						Glyco_hydro_62	PF03664.14		CAZYME
NEMBOFW57_000338	28	11	29	11	52.7	6.28	40.61	11	x						ArabFuran-catal	PF09206.12		CAZYME
NEMBOFW57_001299	19	10	17	10	66.5	6.13	39.66	10	x	x					COesterase	PF00135.29		CAZYME
NEMBOFW57_010418	17	6	11	6	65.2	5.3	38.52	6			x	x		x	GMC_oxred_N	PF00732.20		CAZYME
NEMBOFW57_006599	46	12	20	12	37.2	5.83	38.41	12	x	x	x	x	x		Lipase_GDSL_2	PF13472.7		CAZYME
NEMBOFW57_005247	27	13	15	13	95.3	6.54	37.71	13	x	x					Glyco_hydro_3_C	PF01915.23		CAZYME
NEMBOFW57_007659	33	14	18	14	69.2	7.06	37.71	14	x	x	x	x	x	x	Glyco_hydro_15	PF00723.22		CAZYME
NEMBOFW57_000286	22	12	18	12	56.9	6.92	37.25	12	x	x					Lipase_GDSL_2	PF13472.7		CAZYME
NEMBOFW57_006926	7	7	12	7	147.8	5.5	37.09	7			x	x	x	x	Glyco_hydro_18	PF00704.29		CAZYME
NEMBOFW57_002244	19	4	10	4	44.0	6.04	37.0	4	x		x	x	x	x	SUN	PF03856.14		CAZYME
NEMBOFW57_004007	36	16	21	16	50.2	7.02	36.35	16	x						Glyco_hydro_28	PF00295.18		CAZYME
NEMBOFW57_010449	32	12	13	12	67.1	8.16	35.12	12	x	x	x	x			GMC_oxred_N	PF00732.20		CAZYME
NEMBOFW57_007883	31	16	18	16	77.1	7.06	35.05	16	x	x					Glyco_hydro_3_C	PF01915.23		CAZYME
NEMBOFW57_000683	78	14	29	14	29.3	6.43	33.33	14	x	x					GH131_N	PF18271.2		CAZYME
NEMBOFW57_008527	25	14	15	14	89.2	6.48	32.49	14	x	x					Sortilin-Vps10	PF15902.6		CAZYME
NEMBOFW57_006728	59	14	18	14	35.4	8.84	30.99	14	x	x					Pectinesterase	PF01095.20		CAZYME

NEMBOFW57_010962	33	11	18	10	45.2	7.34	30.7	11	x	x	x		x	x	Glyco_hydro_10	PF00331.21		CAZYME
NEMBOFW57_004002	48	12	17	6	47.4	8.44	30.27	12	x						Glyco_hydro_79C	PF16862.6		CAZYME
NEMBOFW57_010773	45	11	16	11	32.2	8.92	29.92	11	x	x					Pectate_lyase_4	PF00544.20		CAZYME
NEMBOFW57_007876	41	8	22	8	27.2	8.06	28.31	8	x	x					AA9	PF03443.15		CAZYME
NEMBOFW57_008032	34	12	13	12	54.4	8.02	28.22	12	x	x					GMC_oxred_N	PF00732.20		CAZYME
NEMBOFW57_010165	36	10	19	10	45.6	6.16	27.87	10	x	x					Glyco_hydro_7	PF00840.21		CAZYME
NEMBOFW57_004513	38	7	13	7	31.2	8.24	27.34	7	x	x					Esterase_PHB	PF10503.10		CAZYME
NEMBOFW57_009222	20	12	13	12	83.7	6.23	26.9	12	x	x					Glyco_hydro_3	PF00933.22		CAZYME
NEMBOFW57_004115	22	9	14	9	54.2	7.61	26.17	9	x	x					FAD_binding_4	PF01565.24		CAZYME
NEMBOFW57_000575	27	7	10	7	37.5	5.3	25.59	7	x	x					Polysacc_deac_1	PF01522.22		CAZYME
NEMBOFW57_000887	25	12	13	12	74.9	8.51	25.59	12	x	x		x	x		COesterase	PF00135.29		CAZYME
NEMBOFW57_002779	7	9	11	9	176.4	6.28	24.81	9			x	x			hDGE_amylase	PF14701.7		CAZYME
NEMBOFW57_006478	11	11	11	11	142.9	6.77	24.78	11		x		x			Phosphorylase	PF00343.21		CAZYME
NEMBOFW57_002736	32	8	16	8	34.1	8.66	24.65	8	x	x					GH131_N	PF18271.2		CAZYME
NEMBOFW57_002054	40	6	14	6	25.9	8.09	24.57	6	x	x					Esterase_PHB	PF10503.10		CAZYME
NEMBOFW57_009327	29	14	18	14	77.5	5.92	24.15	14	x	x					fn3_3	PF14686.7		CAZYME
NEMBOFW57_001526	33	10	13	10	37.2	5.06	23.8	10	x	x					Glyco_hydro_43	PF04616.15		CAZYME
NEMBOFW57_002602	19	16	18	16	124.6	6.67	23.58	16		x					Glyco_hydro_38N	PF01074.23		CAZYME
NEMBOFW57_002874	33	13	14	13	71.4	6.2	23.24	13	x	x					Glyco_hydro_3_C	PF01915.23		CAZYME
NEMBOFW57_009709	13	7	8	7	80.4	6.23	23.02	7		x		x			Alpha-amylase_C	PF02806.19		CAZYME
NEMBOFW57_007691	16	6	10	6	42.6	6.7	22.79	6	x	x					BNR_2	PF13088.7		CAZYME
NEMBOFW57_002037	24	10	11	10	58.0	5.99	21.95	10	x	x	x	x	x	x	Glyco_hydro_2_N	PF02837.19		CAZYME
NEMBOFW57_003457	27	9	14	9	55.9	7.9	21.49	9	x	x					Glyco_hydro_79C	PF16862.6		CAZYME
NEMBOFW57_008865	18	8	10	8	78.4	6.1	21.31	8		x		x	x		Pectate_lyase_3	PF12708.8		CAZYME
NEMBOFW57_003080	33	12	12	12	49.2	4.78	21.28	12	x	x					Glyco_hydro_7	PF00840.21		CAZYME
NEMBOFW57_004738	11	9	9	9	132.1	6.51	21.21	9	x	x	x	x	x	x	COesterase	PF00135.29		CAZYME
NEMBOFW57_007866	27	7	9	7	40.5	8.44	21.19	7		x	x	x	x	x	Glyco_hydro_18	PF00704.29		CAZYME

NEMBOFW57_000322	20	10	13	10	53.9	5.44	20.37	10	x	x					Glyco_hydro_1	PF00232.19		CAZYME
NEMBOFW57_001184	41	6	11	6	26.9	7.94	19.19	6	x	x					Glyco_hydro_12	PF01670.17		CAZYME
NEMBOFW57_001185	20	9	10	9	73.3	7.88	18.58	9	x	x					Alpha-L-AF_C	PF06964.13		CAZYME
NEMBOFW57_003719	17	2	5	2	23.5	8.88	18.58	2	x	x	x		x	x	Glyco_hydro_11	PF00457.18		CAZYME
NEMBOFW57_007041	8	3	7	3	45.2	5.54	18.34	3			x	x		x	Glyco_hydro_76	PF03663.15		CAZYME
NEMBOFW57_009514	33	7	11	1	46.4	6.86	18.11	7	x						Glyco_hydro_79C	PF16862.6		CAZYME
NEMBOFW57_004236	12	10	10	10	94.6	6.87	17.73	10		x					Glyco_hydro_67M	PF07488.13		CAZYME
NEMBOFW57_004785	14	5	7	5	51.7	6.42	17.62	5		x		x			Glyco_hydro_6	PF01341.18		CAZYME
NEMBOFW57_004027	26	7	7	7	41.1	4.55	17.53	7	x						Glyco_hydro_114	PF03537.14		CAZYME
NEMBOFW57_009578	30	5	10	5	27.2	7.83	17.05	5	x	x				x	Polysacc_deac_1	PF01522.22		CAZYME
NEMBOFW57_009841	13	5	6	5	56.2	7.94	16.52	5	x	x					Glyco_hydro_43	PF04616.15		CAZYME
NEMBOFW57_010770	10	4	5	4	81.3	6.76	16.28	4			x	x	x		Pectate_lyase_3	PF12708.8		CAZYME
NEMBOFW57_008471	25	6	6	6	51.3	5.01	16.14	6	x	x	x		x		Glyco_hydro_47	PF01532.21		CAZYME
NEMBOFW57_002850	10	8	10	8	109.7	6.1	16.02	8		x		x			Glyco_hydro_2_C	PF02836.18		CAZYME
NEMBOFW57_009077	20	7	10	7	45.9	6.87	15.49	7	x	x	x	x	x	x	Glyco_hydro_7	PF00840.21		CAZYME
NEMBOFW57_000190	3	2	4	2	92.2	6.3	15.28	2	x	x	x	x	x		CorA	PF01544.19		CAZYME
NEMBOFW57_006732	12	3	4	2	44.2	5.24	14.67	3		x		x			Glyco_hydro_10	PF00331.21		CAZYME
NEMBOFW57_004103	15	7	7	7	78.1	8.56	14.57	7		x					Glyco_hydro_15	PF00723.22		CAZYME
NEMBOFW57_003857	16	4	6	4	32.2	7.27	14.39	4	x	x					AA9	PF03443.15		CAZYME
NEMBOFW57_010245	12	3	7	3	23.6	8.68	14.38	3		x				x	AA9	PF03443.15		CAZYME
NEMBOFW57_004987	8	5	5	5	71.9	4.79	14.29	5	x	x		x			Mitofilin	PF09731.10		CAZYME
NEMBOFW57_007947	16	4	4	4	56.2	8.34	14.1	4	x		x		x		GMC_oxred_N	PF00732.20		CAZYME
NEMBOFW57_001690	9	4	4	4	96.0	4.42	14.06	4	x	x		x			LysM	PF01476.21		CAZYME
NEMBOFW57_003505	8	5	5	5	89.3	5.74	13.56	5	x	x					Glyco_hydro_3_C	PF01915.23		CAZYME
NEMBOFW57_006469	12	6	8	6	84.8	6.04	13.12	6	x	x					Polysacc_deac_1	PF01522.22		CAZYME
NEMBOFW57_003741	18	5	5	5	44.1	8.7	12.49	5	x	x	x	x		x	AXE1	PF05448.13		CAZYME
NEMBOFW57_010681	9	6	6	6	101.5	5.76	12.47	6		x					Ig_GlcNase	PF18368.2		CAZYME

NEMBOFW57_002920	9	5	7	5	52.0	5.71	12.4	5	x	x					Glyco_hydro_26	PF02156.16		CAZYME
NEMBOFW57_005718	27	4	6	4	26.0	6.2	12.36	4	x	x					Polysacc_deac_1	PF01522.22		CAZYME
NEMBOFW57_003773	38	10	10	10	36.3	8.27	11.86	10	x	x					Melibiase_2	PF16499.6		CAZYME
NEMBOFW57_008260	65	5	9	5	19.9	7.02	11.84	5	x	x					Lipase_GDSL_2	PF13472.7		CAZYME
NEMBOFW57_000134	25	10	10	10	60.6	7.93	11.83	10	x						Glyco_hydro_43	PF04616.15		CAZYME
NEMBOFW57_008132	21	6	7	6	36.3	6.89	11.61	6	x						Glyco_hydro_79C	PF16862.6		CAZYME
NEMBOFW57_002928	36	5	8	5	21.8	6.54	11.58	5	x	x					FMN_red	PF03358.16		CAZYME
NEMBOFW57_009419	20	7	7	7	46.6	7.53	11.54	7	x	x					Cellulase	PF00150.19		CAZYME
NEMBOFW57_001193	23	9	9	9	40.3	7.81	11.26	9	x	x					Cellulase	PF00150.19		CAZYME
NEMBOFW57_008059	18	3	3	3	33.2	8.21	11.13	3					x		AA9	PF03443.15		CAZYME
NEMBOFW57_001853	4	5	8	5	145.4	5.64	10.77	5	x	x					Glyco_hydro_35	PF01301.20		CAZYME
NEMBOFW57_008720	19	3	8	3	23.2	5.5	10.63	3	x	x					CDH-cyt	PF16010.6		CAZYME
NEMBOFW57_004659	15	5	8	5	36.1	9.07	10.12	5	x	x					GH131_N	PF18271.2		CAZYME
NEMBOFW57_004532	25	5	6	5	27.7	5.67	10.03	5	x						Phage_lysozyme	PF00959.20		CAZYME
NEMBOFW57_002775	28	4	5	4	28.7	6.61	10.02	4	x	x					GMC_oxred_C	PF05199.14		CAZYME
NEMBOFW57_007936	27	4	6	3	23.3	8.0	10.02	4	x	x					Cutinase	PF01083.23		CAZYME
NEMBOFW57_002197	11	5	6	5	51.8	8.34	9.38	5	x	x					COesterase	PF00135.29		CAZYME
NEMBOFW57_010955	8	2	6	1	31.7	7.94	9.37	2		x	x	x	x	x	Cutinase	PF01083.23		CAZYME
NEMBOFW57_006922	9	3	4	3	47.4	6.64	9.24	3	x	x					LysM	PF01476.21		CAZYME
NEMBOFW57_002500	27	6	10	6	23.7	7.42	9.23	6	x	x					Glyco_hydro_45	PF02015.17		CAZYME
NEMBOFW57_000554	5	3	3	3	106.0	6.24	9.22	3				x			HK	PF02110.16		CAZYME
NEMBOFW57_001379	10	5	6	5	66.7	8.75	9.21	5	x						Glyco_hydro_127	PF07944.13		CAZYME
NEMBOFW57_006928	33	8	8	8	47.0	4.7	8.94	8	x						Glyco_hydro_75	PF07335.12		CAZYME
NEMBOFW57_004860	8	5	7	5	99.4	6.21	8.87	5	x	x	x		x	x	Glyco_hydro_31	PF01055.27		CAZYME
NEMBOFW57_004634	5	4	4	3	71.1	7.55	7.96	4	x		x	x			GMC_oxred_N	PF00732.20		CAZYME
NEMBOFW57_007800	33	6	10	6	33.5	8.03	7.86	6	x	x					Esterase_PHB	PF10503.10		CAZYME
NEMBOFW57_007945	9	3	3	3	61.2	6.13	7.8	3	x	x					Glyco_hydro_43	PF04616.15		CAZYME

NEMBOFW57_008512	10	4	4	4	62.6	6.49	7.74	4	x	x					Glyco_hydro_43	PF04616.15		CAZYME
NEMBOFW57_003636	13	4	5	4	50.1	6.37	7.73	4		x					Glyco_hydro_7	PF00840.21		CAZYME
NEMBOFW57_010597	10	1	2	1	24.1	8.16	7.6	1				x	x		AA9	PF03443.15		CAZYME
NEMBOFW57_008762	11	4	4	4	48.2	5.74	7.28	4	x						Melibiase_2	PF16499.6		CAZYME
NEMBOFW57_009601	9	2	3	2	41.7	6.54	7.06	2		x					CBM_1	PF00734.19		CAZYME
NEMBOFW57_002906	25	4	5	4	39.6	8.13	6.86	4		x		x		x	Esterase_PHB	PF10503.10		CAZYME
NEMBOFW57_002909	11	5	5	5	60.5	5.48	6.83	5	x	x					Glyco_hydro_3	PF00933.22		CAZYME
NEMBOFW57_002035	12	3	3	3	33.0	7.78	6.56	3		x					AA9	PF03443.15		CAZYME
NEMBOFW57_002908	10	2	3	2	31.3	9.45	6.55	2	x	x					Glyco_hydro_3_C	PF01915.23		CAZYME
NEMBOFW57_008083	9	5	5	5	68.5	8.76	6.53	5	x	x					GMC_oxred_N	PF00732.20		CAZYME
NEMBOFW57_009223	4	3	3	3	85.4	5.48	6.22	3	x	x		x			Glyco_hydro_31	PF01055.27		CAZYME
NEMBOFW57_009241	8	2	2	2	34.3	8.37	5.99	2	x						Glyco_hydro_43	PF04616.15		CAZYME
NEMBOFW57_004792	9	3	4	3	44.9	8.22	5.99	3		x	x		x		AXE1	PF05448.13		CAZYME
NEMBOFW57_004293	10	2	2	2	39.7	5.8	5.78	2		x					Cellulase	PF00150.19		CAZYME
NEMBOFW57_002792	4	3	3	3	99.8	9.42	5.78	3		x					COesterase	PF00135.29		CAZYME
NEMBOFW57_004244	6	3	3	3	58.9	5.91	5.76	3		x		x			Glyco_transf_20	PF00982.22		CAZYME
NEMBOFW57_002223	12	4	4	4	38.4	8.79	5.58	4	x						Glyco_hydro_53	PF07745.14		CAZYME
NEMBOFW57_001268	20	6	6	6	50.5	8.7	5.47	6	x						Glyco_hydro_43	PF04616.15		CAZYME
NEMBOFW57_004373	21	5	5	5	41.9	8.15	5.37	5	x									CAZYME
NEMBOFW57_003584	9	2	2	2	35.3	6.11	5.31	2		x					CBM_1	PF00734.19		CAZYME
NEMBOFW57_009003	12	3	3	3	34.2	7.37	5.21	3	x	x					Pectate_lyase	PF03211.14		CAZYME
NEMBOFW57_009999	7	2	2	2	40.7	7.65	5.12	2	x						Glyco_hydro_62	PF03664.14		CAZYME
NEMBOFW57_009019	22	5	9	4	31.7	7.83	5.09	5		x					Glyco_hydro_45	PF02015.17		CAZYME
NEMBOFW57_007820	4	3	3	3	95.1	6.54	5.01	3		x					Glyco_hydro_31	PF01055.27		CAZYME
NEMBOFW57_009275	5	2	2	2	61.9	6.13	4.78	2		x					COesterase	PF00135.29		CAZYME
NEMBOFW57_009693	15	4	4	4	36.3	6.96	4.74	4	x	x					Pectate_lyase_4	PF00544.20		CAZYME
NEMBOFW57_007166	4	3	3	1	78.6	5.59	4.64	3		x				x	GMC_oxred_N	PF00732.20		CAZYME

NEMBOFW57_000278	13	3	3	3	31.7	6.06	4.53	3		x					Esterase	PF00756.21		CAZYME
NEMBOFW57_008456	5	2	2	1	48.9	5.31	4.52	2		x					Glyco_hydro_7	PF00840.21		CAZYME
NEMBOFW57_009130	8	3	3	3	57.0	5.34	4.19	3	x	x					Glyco_hydro_125	PF06824.12		CAZYME
NEMBOFW57_007796	7	4	4	2	69.5	6.46	3.99	4		x					GMC_oxred_C	PF05199.14		CAZYME
NEMBOFW57_002806	8	3	4	3	44.0	7.05	3.88	3		x					Cellulase	PF00150.19		CAZYME
NEMBOFW57_001116	11	5	5	5	63.3	8.46	3.87	5	x	x					FAD_binding_4	PF01565.24		CAZYME
NEMBOFW57_006970	13	3	4	3	24.6	4.54	3.85	3		x					Glyco_hydro_18	PF00704.29		CAZYME
NEMBOFW57_004619	14	5	6	5	54.4	7.02	3.77	5	x						Glyco_hydro_43	PF04616.15		CAZYME
NEMBOFW57_004186	3	1	1	1	44.9	6.11	3.76	1				x			Glyco_hydro_18	PF00704.29		CAZYME
NEMBOFW57_008515	16	3	3	3	29.6	9.38	3.72	3	x									CAZYME
NEMBOFW57_001464	4	2	2	2	63.1	6.19	3.71	2	x						FAD_binding_4	PF01565.24		CAZYME
NEMBOFW57_009225	8	2	2	2	33.5	8.7	3.7	2	x						GMC_oxred_N	PF00732.20		CAZYME
NEMBOFW57_005252	2	1	1	1	110.7	5.06	3.59	1			x				WSC	PF01822.20		CAZYME
NEMBOFW57_010266	10	2	2	2	22.9	8.02	3.04	2	x	x					LPMO_10	PF03067.16		CAZYME
NEMBOFW57_007260	3	1	1	1	46.3	7.03	2.89	1				x			SUN	PF03856.14		CAZYME
NEMBOFW57_003172	7	2	2	2	33.1	9.01	2.72	2	x			x	x		Glyco_hydro_16	PF00722.22		CAZYME
NEMBOFW57_004769	7	2	3	2	45.9	6.67	2.58	2	x	x					Cellulase	PF00150.19		CAZYME
NEMBOFW57_003132	4	1	2	1	39.6	8.18	2.58	1	x	x					peroxidase	PF00141.24		CAZYME
NEMBOFW57_006721	7	2	2	2	54.0	6.84	2.26	2	x						Glyco_hyd_65N_2	PF14498.7		CAZYME
NEMBOFW57_004511	2	1	1	1	82.6	6.57	2.26	1		x					AA9	PF03443.15		CAZYME
NEMBOFW57_003918	0	1	1	1	220.7	7.85	2.22	1				x			Glucan_synthase	PF02364.16		CAZYME
NEMBOFW57_005701	3	1	1	1	24.8	8.4	2.21	1	x						AA9	PF03443.15		CAZYME
NEMBOFW57_001172	9	2	2	2	25.8	6.39	2.11	2	x						AA9	PF03443.15		CAZYME
NEMBOFW57_008589	9	1	1	1	35.8	9.64	2.09	1	x						Pectate_lyase_4	PF00544.20		CAZYME
NEMBOFW57_003627	14	2	3	2	34.3	8.5	2.04	2	x						Glyco_hydro_43	PF04616.15		CAZYME
NEMBOFW57_009318	22	5	5	5	30.7	6.79	2.01	5	x	x					Cellulase	PF00150.19		CAZYME
NEMBOFW57_007618	12	2	2	2	36.0	8.88	2.0	2	x						BNR	PF02012.21		CAZYME

NEMBOFW57_009121	4	1	1	1	23.1	6.18	2.0	1	x	x					Glyco_hydro_16	PF00722.22		CAZYME
NEMBOFW57_006439	6	3	3	3	70.5	8.16	1.88	3	x						CBM-like	PF14683.7		CAZYME
NEMBOFW57_006737	10	3	3	3	32.0	5.92	1.88	3		x					Glyco_hydro_16	PF00722.22		CAZYME
NEMBOFW57_007038	18	3	4	3	38.1	6.3	1.87	3	x	x					CE2_N	PF17996.2		CAZYME
NEMBOFW57_009711	4	1	1	1	28.7	8.48	1.84	1	x						Esterase_PHB	PF10503.10		CAZYME
NEMBOFW57_009348	4	1	1	1	21.3	6.28	1.82	1		x					Abhydrolase_2	PF02230.17		CAZYME
NEMBOFW57_007690	6	2	2	2	55.5	7.69	1.81	2		x					Glyco_hydro_12	PF01670.17		CAZYME
NEMBOFW57_003635	2	1	1	1	41.4	7.28	1.8	1		x					Glyco_hydro_10	PF00331.21		CAZYME
NEMBOFW57_008340	4	1	2	1	25.1	9.13	1.73	1	x						Glyco_hydro_11	PF00457.18		CAZYME
NEMBOFW57_002052	15	3	3	3	38.5	7.96	1.67	3		x					Esterase_PHB	PF10503.10		CAZYME
NEMBOFW57_002998	3	1	1	1	37.4	9.09	1.65	1						x	Glyco_hydro_18	PF00704.29		CAZYME
NEMBOFW57_010300	2	1	1	1	63.4	5.71	1.62	1	x	x					Cu-oxidase_3	PF07732.16		CAZYME
NEMBOFW57_007118	4	3	3	3	89.8	5.25	0.0	3	x						Glyco_hydro_92	PF07971.13		CAZYME
NEMBOFW57_001166	9	2	2	2	34.5	7.17	0.0	2	x						Esterase_PHB	PF10503.10		CAZYME
NEMBOFW57_007869	5	2	2	2	49.5	8.66	0.0	2	x						Glyco_hydro_43	PF04616.15		CAZYME
NEMBOFW57_003655	4	1	1	1	36.3	8.34	0.0	1	x						Glyco_hydro_10	PF00331.21		CAZYME
NEMBOFW57_007080	6	1	1	1	20.3	5.49	0.0	1	x						Pectate_lyase_4	PF00544.20		CAZYME
NEMBOFW57_009294	2	1	1	1	56.9	5.49	0.0	1	x						Alpha-L-AF_C	PF06964.13		CAZYME
NEMBOFW57_001206	4	2	2	2	67.3	6.87	0.0	2		x					Cu-oxidase_3	PF07732.16		CAZYME
NEMBOFW57_009546	4	1	1	1	40.8	7.31	0.0	1		x					Glyco_hydro_10	PF00331.21		CAZYME
NEMBOFW57_008990	3	1	1	1	86.8	6.07	0.0	1		x					NHL	PF01436.22		CAZYME
NEMBOFW57_008747	2	1	1	1	77.5	5.34	0.0	1		x					Glyco_hydro_3_C	PF01915.23		CAZYME
NEMBOFW57_004294	4	1	1	1	36.5	8.09	0.0	1		x					CBM_1	PF00734.19		CAZYME
NEMBOFW57_004390	3	1	1	1	45.1	6.09	0.0	1				x			Glyco_hydro_cc	PF11790.9		CAZYME
NEMBOFW57_004480	24	19	20	19	101.0	8.05	25.45	19	x	x								CAZYME
NEMBOFW57_002584	69	19	85	19	46.1	5.31	322.28	19	x	x	x	x	x	x	Enolase_C	PF00113.23		Energy metabolism

NEMBOFW57_006748	51	18	74	18	44.5	7.11	261.11	18	x	x	x	x	x	x	PGK	PF00162.20		Energy metabolism
NEMBOFW57_004828	41	11	47	11	40.4	5.83	200.48	11	x	x	x	x	x	x	F_bP_aldolase	PF01116.21		Energy metabolism
NEMBOFW57_008695	44	14	49	14	52.3	7.5	185.67	14	x	x	x	x	x	x	Iso_dh	PF00180.21		Energy metabolism
NEMBOFW57_001745	46	15	44	15	35.1	5.66	169.19	15	x	x	x	x	x	x	TAL_FSA	PF00923.20		Energy metabolism
NEMBOFW57_006037	37	12	43	12	60.5	7.53	167.53	12	x	x	x	x	x	x	ICL	PF00463.22		Energy metabolism
NEMBOFW57_007133	34	12	39	12	60.8	6.7	148.53	12	x	x	x	x	x	x	PGI	PF00342.20		Energy metabolism
NEMBOFW57_009613	57	18	48	18	41.6	6.01	113.83	18	x	x					Aldose_epim	PF01263.21		Energy metabolism
NEMBOFW57_000294	62	15	32	14	34.3	6.58	113.48	15	x	x	x	x	x	x	Ldh_1_C	PF02866.19		Energy metabolism
NEMBOFW57_002476	28	10	30	10	57.0	6.8	103.42	10	x	x	x	x	x	x	6PGD	PF00393.20		Energy metabolism
NEMBOFW57_009946	64	19	48	19	36.2	7.39	95.4	19	x	x					Gp_dh_C	PF02800.21		Energy metabolism
NEMBOFW57_001429	36	11	28	11	52.3	8.68	88.63	11		x	x	x	x	x	Citrate_synt	PF00285.22		Energy metabolism
NEMBOFW57_006303	49	13	37	13	40.6	7.96	81.08	13	x	x					Dioxygenase_C	PF00775.22		Energy metabolism
NEMBOFW57_001718	37	6	18	6	34.6	7.78	72.34	6		x	x	x	x	x	MmgE_PrpD	PF03972.15		Energy metabolism
NEMBOFW57_003824	26	8	20	8	56.7	5.59	66.34	8		x	x	x	x	x	iPGM_N	PF06415.14		Energy metabolism

NEMBOFW57_003890	18	13	22	13	92.1	6.57	65.26	13	x	x	x	x	x	x	XFP_N	PF09364.11		Energy metabolism
NEMBOFW57_009772	34	21	34	21	73.7	6.47	63.95	21	x	x		x			AMP-binding	PF00501.29	acetate--CoA ligase [Thermothielavioides terrestris NRRL 8126]	Energy metabolism
NEMBOFW57_008876	42	7	16	7	32.9	5.99	62.9	7	x	x	x	x	x	x	Glyoxalase	PF00903.26		Energy metabolism
NEMBOFW57_004367	29	10	22	10	52.2	8.22	62.61	10	x	x	x	x	x	x	Citrate_synt	PF00285.22		Energy metabolism
NEMBOFW57_010533	28	16	23	16	84.8	7.01	54.05	16	x	x	x	x		x	Aconitase	PF00330.21		Energy metabolism
NEMBOFW57_007899	43	13	25	12	35.4	8.37	50.56	13	x	x		x			Ldh_1_C	PF02866.19		Energy metabolism
NEMBOFW57_009861	21	4	16	4	11.7	9.19	48.8	4	x		x	x	x	x	Cytochrom_C	PF00034.22		Energy metabolism
NEMBOFW57_009796	30	9	20	9	44.3	5.92	44.23	9	x	x					Aldose_epim	PF01263.21		Energy metabolism
NEMBOFW57_005119	22	9	10	9	72.4	6.32	40.09	9	x	x	x	x		x	Transketolase_N	PF00456.22		Energy metabolism
NEMBOFW57_001469	26	8	11	8	61.1	7.14	38.45	8			x	x		x	ICL	PF00463.22		Energy metabolism
NEMBOFW57_000867	36	8	11	8	27.0	5.54	37.2	8	x	x	x	x	x	x	TIM	PF00121.19		Energy metabolism
NEMBOFW57_004369	8	6	8	6	98.8	5.22	29.26	6		x	x	x			E1-E2_ATPase	PF00122.21		Energy metabolism
NEMBOFW57_006365	14	4	7	4	48.9	5.29	26.17	4				x			PMI_typeI	PF01238.22		Energy metabolism
NEMBOFW57_003315	31	6	9	6	33.8	5.3	25.9	6	x	x	x	x	x	x	Aldose_epim	PF01263.21		Energy metabolism

NEMBOFW57_003087	22	4	7	4	29.9	6.04	24.79	4		x		x		x	PMM	PF03332.14		Energy metabolism
NEMBOFW57_001645	18	8	10	8	60.9	8.38	24.58	8		x	x	x			Malate_synthase	PF01274.23		Energy metabolism
NEMBOFW57_007789	29	6	8	6	29.3	5.52	23.64	6	x	x	x	x	x	x	Rib_5-P_isom_A	PF06026.15		Energy metabolism
NEMBOFW57_000528	21	4	7	4	38.7	5.58	21.05	4	x	x	x	x		x	Iso_dh	PF00180.21		Energy metabolism
NEMBOFW57_008852	9	4	10	4	65.5	6.11	18.33	4	x	x					PEPCK_ATP	PF01293.21		Energy metabolism
NEMBOFW57_009220	20	3	5	3	36.4	5.73	16.34	3				x	x	x	HpcH_HpaI	PF03328.15		Energy metabolism
NEMBOFW57_007991	16	3	4	3	32.6	6.52	13.32	3			x	x	x		HpcH_HpaI	PF03328.15		Energy metabolism
NEMBOFW57_000478	23	4	5	4	28.4	5.24	12.77	4		x		x			Glucosamine_iso	PF01182.21		Energy metabolism
NEMBOFW57_002646	10	4	4	4	59.6	6.49	12.68	4		x		x			PGM_PMM_I	PF02878.17		Energy metabolism
NEMBOFW57_003985	15	6	7	6	57.9	6.57	12.2	6	x	x					PK	PF00224.22		Energy metabolism
NEMBOFW57_002141	22	3	3	3	27.1	5.52	11.9	3			x	x	x	x	Ribul_P_3_epim	PF00834.20		Energy metabolism
NEMBOFW57_004223	46	9	9	9	36.5	5.07	9.7	9	x	x		x			Aldose_epim	PF01263.21		Energy metabolism
NEMBOFW57_002822	11	3	5	3	42.7	8.87	9.49	3	x	x					Iso_dh	PF00180.21		Energy metabolism
NEMBOFW57_001618	3	1	2	1	68.1	5.47	9.26	1				x			HMGL-like	PF00682.20		Energy metabolism

NEMBOFW57_004455	8	4	5	4	53.1	6.3	8.9	4	x	x					Citrate_bind	PF16114.6		Energy metabolism
NEMBOFW57_010857	31	4	4	4	16.5	8.38	8.02	4	x	x					Isochorismatase	PF00857.21		Energy metabolism
NEMBOFW57_002459	6	1	3	1	28.9	5.88	7.09	1				x			HpcH_HpaI	PF03328.15		Energy metabolism
NEMBOFW57_010459	15	3	3	3	19.6	4.94	6.38	3	x	x				x	FKBP_C	PF00254.29		Energy metabolism
NEMBOFW57_001303	16	4	4	4	28.4	5.87	6.13	4	x	x					PEP_mutase	PF13714.7		Energy metabolism
NEMBOFW57_003165	19	8	9	8	58.1	7.03	6.06	8	x	x					G6PD_C	PF02781.17		Energy metabolism
NEMBOFW57_009706	1	1	2	1	117.3	6.8	5.05	1	x	x	x				Transket_pyr	PF02779.25		Energy metabolism
NEMBOFW57_010920	5	3	3	3	89.5	5.96	4.66	3		x					XFP_N	PF09364.11		Energy metabolism
NEMBOFW57_009289	7	3	3	3	62.2	6.49	4.47	3		x					FAD_binding_2	PF00890.25	Fumarate reductase 2 [Madurella mycetomatis]	Energy metabolism
NEMBOFW57_003064	5	1	1	1	28.9	6.44	3.42	1				x			Aldolase_II	PF00596.22		Energy metabolism
NEMBOFW57_002393	3	1	1	1	40.6	6.86	3.36	1				x			DAHP_synth_1	PF00793.21		Energy metabolism
NEMBOFW57_001863	2	2	2	2	92.2	7.61	3.34	2		x		x			PFK	PF00365.21		Energy metabolism
NEMBOFW57_009875	5	2	2	2	46.0	5.41	3.19	2	x	x		x		x	Glyoxalase	PF00903.26		Energy metabolism
NEMBOFW57_000555	10	1	1	1	21.9	7.42	3.09	1				x			Isochorismatase	PF00857.21		Energy metabolism

NEMBOFW57_007364	6	1	2	1	41.2	8.46	2.71	1	x	x					Iso_dh	PF00180.21		Energy metabolism
NEMBOFW57_001229	4	1	1	1	71.7	6.57	2.66	1				x			FAD_binding_3	PF01494.20	Pyruvate dehydrogenase E1 component subunit beta, mitochondrial [Madurella mycetomatis]	Energy metabolism
NEMBOFW57_001970	6	2	2	2	40.7	7.3	2.66	2		x		x			Transket_pyr	PF02779.25		Energy metabolism
NEMBOFW57_009795	4	2	2	2	56.0	5.54	2.49	2				x			ATP-synt_ab	PF00006.26		Energy metabolism
NEMBOFW57_010045	4	1	1	1	37.3	9.57	2.35	1				x			Epimerase	PF01370.22		Energy metabolism
NEMBOFW57_008904	4	1	1	1	37.8	6.06	2.23	1	x						FBPase	PF00316.21		Energy metabolism
NEMBOFW57_001919	2	1	1	1	57.5	5.43	0.0	1		x					Hexokinase_1	PF00349.22		Energy metabolism
NEMBOFW57_006667	4	1	1	1	41.0	6.61	0.0	1		x					Epimerase	PF01370.22		Energy metabolism
NEMBOFW57_007193	3	1	1	1	31.2	4.98	0.0	1		x					F_bP_aldolase	PF01116.21		Energy metabolism
NEMBOFW57_005802	19	8	16	8	63.9	8.05	51.74	8		x	x	x	x	x	ILVD_EDD	PF00920.22		Lipid metabolism
NEMBOFW57_008976	30	15	17	15	80.9	6.55	41.15	15	x	x	x	x	x	x	Ceramidase_alk	PF04734.14		Lipid metabolism
NEMBOFW57_003158	39	11	16	11	46.2	8.03	38.8	11	x	x					Lipase_GDSL_2	PF13472.7		Lipid metabolism
NEMBOFW57_006771	20	9	10	9	70.5	7.36	27.31	9	x	x					COesterase	PF00135.29		Lipid metabolism
NEMBOFW57_002516	10	5	7	5	69.1	5.19	22.06	5	x	x	x	x	x	x	PLA2_B	PF01735.19		Lipid metabolism
NEMBOFW57_005494	22	7	9	7	46.0	7.08	17.83	7	x	x					Lipase_GDSL_2	PF13472.7		Lipid metabolism
NEMBOFW57_009882	28	10	11	10	57.8	6.86	13.41	10	x	x	x	x	x	x	COesterase	PF00135.29		Lipid metabolism
NEMBOFW57_005455	9	3	5	3	49.8	9.11	4.94	3		x					Acyl-CoA_dh_1	PF00441.25		Lipid metabolism

NEMBOFW57_001169	0	1	2	1	256.4	6.57	4.86	1				x			ACC_central	PF08326.13		Lipid metabolism
NEMBOFW57_005021	4	2	2	2	79.1	6.16	4.2	2		x					Ceramidase_alk	PF04734.14		Lipid metabolism
NEMBOFW57_005248	8	3	3	3	35.5	5.63	3.62	3		x					Lipase_3	PF01764.26		Lipid metabolism
NEMBOFW57_008069	2	1	1	1	62.8	6.7	3.02	1			x				Abhydrolase_4	PF08386.11		Lipid metabolism
NEMBOFW57_005534	4	1	1	1	37.6	5.57	2.77	1		x					Abhydrolase_3	PF07859.14		Lipid metabolism
NEMBOFW57_006648	1	1	1	1	204.2	6.2	1.75	1		x					Fas_alpha_ACP	PF18325.2		Lipid metabolism
NEMBOFW57_010938	5	1	1	1	39.3	5.35	0.0	1		x					FAA_hydrolase	PF01557.19		Lipid metabolism
NEMBOFW57_002854	22	4	20	4	30.8	7.8	84.81	4			x	x	x	x			hypothetical protein CHGG_01263 [Chaetomium globosum CBS 148.51]	NON CONSERVED PATTERN
NEMBOFW57_010651	43	10	30	10	20.5	6.54	64.13	10	x	x	x	x	x	x			hypothetical protein MMYC01_205089 [Madurella mycetomatis]	NON CONSERVED PATTERN
NEMBOFW57_000156	46	7	24	7	26.5	6.67	51.29	7	x	x			x				hypothetical protein CHGG_05466 [Chaetomium globosum CBS 148.51]	NON CONSERVED PATTERN
NEMBOFW57_002711	42	5	16	5	21.4	6.11	47.15	5		x	x	x		x			uncharacterized protein PODANS_1_6850 [Podospora anserina S mat+]	NON CONSERVED PATTERN
NEMBOFW57_003995	24	2	14	2	9.9	7.83	26.07	2	x	x							Putative protein of unknown function [Podospora comata]	NON CONSERVED PATTERN
NEMBOFW57_003347	48	10	11	10	19.1	5.06	21.41	10	x	x							hypothetical protein CHGG_01803 [Chaetomium globosum CBS 148.51]	NON CONSERVED PATTERN
NEMBOFW57_001211	41	7	13	7	23.7	8.53	19.27	7	x	x	x	x	x	x			hypothetical protein CHGG_08078 [Chaetomium globosum CBS 148.51]	NON CONSERVED PATTERN

NEMBOFW57_002979	13	2	6	2	23.8	4.94	18.18	2	x	x	x	x	x	x			predicted protein [Chaetomium globosum CBS 148.51]	NON CONSERVED PATTERN
NEMBOFW57_008003	22	6	9	6	44.6	7.5	14.2	6	x	x							hypothetical protein CHGG_04436 [Chaetomium glo-bosum CBS 148.51]	NON CONSERVED PATTERN
NEMBOFW57_003599	31	7	7	7	34.7	6.76	13.76	7	x	x							hypothetical protein CHGG_01992 [Chaetomium glo-bosum CBS 148.51]	NON CONSERVED PATTERN
NEMBOFW57_004865	5	1	4	1	26.5	5.88	12.81	1			x	x	x	x			hypothetical protein NCU04603 [Neurospora crassa OR74A]	NON CONSERVED PATTERN
NEMBOFW57_006925	11	2	3	2	33.8	9.67	10.99	2			x	x	x				hypothetical protein DL767_005724 [Monosporascus sp. MG133]	NON CONSERVED PATTERN
NEMBOFW57_005291	26	2	6	2	10.2	7.02	10.95	2		x							Putative protein of unknown function [Podospora comata]	NON CONSERVED PATTERN
NEMBOFW57_002919	28	4	7	4	19.7	7.42	9.07	4		x							Cu,Zn superoxide dismutase-like protein [Neurospora tetrasperma FGSC 2509]	NON CONSERVED PATTERN
NEMBOFW57_004728	28	3	7	3	14.6	5.24	8.3	3		x							predicted protein [Chaetomium globosum CBS 148.51]	NON CONSERVED PATTERN
NEMBOFW57_003604	18	3	5	3	18.9	7.71	7.94	3	x	x							predicted protein [Chaetomium globosum CBS 148.51]	NON CONSERVED PATTERN
NEMBOFW57_005507	17	3	4	3	22.1	7.21	6.35	3	x	x							hypothetical protein CHGG_08369 [Chaetomium glo-bosum CBS 148.51]	NON CONSERVED PATTERN
NEMBOFW57_006302	23	1	2	1	8.1	9.33	5.78	1			x	x					Putative protein of unknown function [Podospora comata]	NON CONSERVED PATTERN

NEMBOFW57_009194	17	2	3	2	19.0	8.92	5.6	2	x	x							hypothetical protein SAPIO_CDS8815 [Scedosporium apiospermum]	NON CONSERVED PATTERN
NEMBOFW57_000064	14	2	2	2	18.4	6.76	4.61	2	x								hypothetical protein PUNSTDRAFT_55734 [Punctularia strigosozonata HHB-11173 SS5]	NON CONSERVED PATTERN
NEMBOFW57_003593	5	1	1	1	34.5	5.39	4.37	1			x	x	x				hypothetical protein DL546_005043 [Coniochaeta pul-veracea]	NON CONSERVED PATTERN
NEMBOFW57_001308	23	5	5	5	32.5	6.81	4.36	5	x	x							hypothetical protein CHGG_03685 [Chaetomium glo-bosum CBS 148.51]	NON CONSERVED PATTERN
NEMBOFW57_000161	4	1	1	1	53.6	5.63	3.98	1				x					hypothetical protein MMYC01_201089 [Madurella mycetomatis]	NON CONSERVED PATTERN
NEMBOFW57_004177	7	1	1	1	27.9	8.76	3.17	1			x						hypothetical protein MMYC01_208396 [Madurella mycetomatis]	NON CONSERVED PATTERN
NEMBOFW57_004962	5	1	1	1	48.8	5.35	3.03	1				x					uncharacterized protein THITE_2106442 [Thermothielavi-oides terrestris NRRL 8126]	NON CONSERVED PATTERN
NEMBOFW57_006948	6	1	1	1	35.5	5.5	2.88	1				x					hypothetical protein CHGG_00621 [Chaetomium glo-bosum CBS 148.51]	NON CONSERVED PATTERN
NEMBOFW57_001076	3	1	1	1	30.4	5.12	2.86	1	x	x							hypothetical protein [Mycena sanguinolenta]	NON CONSERVED PATTERN

NEMBOFW57_000279	9	1	1	1	11.0	4.98	2.71	1	x	x							uncharacterized protein THITE_2171055 [Thermothielavioides terrestris NRRL 8126]	NON CONSERVED PATTERN
NEMBOFW57_010657	29	2	2	2	16.5	5.08	2.67	2	x								hypothetical protein DL767_006635 [Monosporascus sp. MG133]	NON CONSERVED PATTERN
NEMBOFW57_009312	10	2	2	2	28.3	6.07	2.59	2	x	x							hypothetical protein MMYC01_210652 [Madurella mycetomatis]	NON CONSERVED PATTERN
NEMBOFW57_009268	10	2	2	2	32.1	4.96	2.13	2	x								hypothetical protein CHGG_02164 [Chaetomium globosum CBS 148.51]	NON CONSERVED PATTERN
NEMBOFW57_009755	6	1	1	1	32.0	6.14	2.09	1		x							Uncharacterized protein in dcmA 3'region [Madurella mycetomatis]	NON CONSERVED PATTERN
NEMBOFW57_001296	3	1	1	1	30.1	5.5	2.09	1				x					hypothetical protein CGCVW01_v014413 [Colletotrichum viniferum]	NON CONSERVED PATTERN
NEMBOFW57_007044	1	1	1	1	106.8	6.24	1.87	1				x					hypothetical protein CIMG_09306 [Coccidioides immitis RS]	NON CONSERVED PATTERN
NEMBOFW57_001071	20	2	2	2	17.5	5.76	1.79	2		x							predicted protein [Chaetomium globosum CBS 148.51]	NON CONSERVED PATTERN
NEMBOFW57_008930	6	1	1	1	18.6	6.52	1.66	1	x								hypothetical protein MMYC01_204064 [Madurella mycetomatis]	NON CONSERVED PATTERN

NEMBOFW57_004550	2	1	1	1	139.3	4.42	0.0	1	x								uncharacterized protein THITE_2124634 [Thermothielavioides terrestris NRRL 8126]	NON CONSERVED PATTERN
NEMBOFW57_001633	2	1	1	1	35.3	6.46	0.0	1	x								hypothetical protein E8E14_014929 [Neopestalotiopsis sp. 37M]	NON CONSERVED PATTERN
NEMBOFW57_002801	4	1	1	1	34.2	7.44	0.0	1		x							hypothetical protein MMYC01_202648 [Madurella mycetomatis]	NON CONSERVED PATTERN
NEMBOFW57_000126	15	1	1	1	13.4	5.12	0.0	1		x							hypothetical protein MMYC01_207442 [Madurella mycetomatis]	NON CONSERVED PATTERN
NEMBOFW57_001353	38	4	12	4	20.5	7.24	40.81	4	x	x	x	x	x	x	Cupin_5	PF06172.12		Other biological process
NEMBOFW57_005811	33	20	67	20	79.5	4.97	247.45	20	x	x	x	x	x	x	HSP90	PF00183.19		Other biological process
NEMBOFW57_007777	34	13	42	13	49.1	6.18	147.56	13	x	x	x	x	x	x	AdoHcyase	PF05221.18		Other biological process
NEMBOFW57_009732	33	17	39	17	62.4	6.02	138.12	17	x	x	x	x	x	x	Cpn60_TCP1	PF00118.25		Other biological process
NEMBOFW57_008333	44	9	38	9	35.8	5.87	136.44	9		x	x	x	x	x	Aha1_N	PF09229.12		Other biological process
NEMBOFW57_000919	30	14	36	14	86.2	6.6	123.06	14	x	x	x	x	x	x	Meth_synt_2	PF01717.19		Other biological process
NEMBOFW57_010755	54	13	27	13	33.1	7.11	102.79	13	x	x	x	x	x	x	NmrA	PF05368.14		Other biological process
NEMBOFW57_009733	52	6	22	6	17.9	7.18	101.44	6	x	x	x	x	x	x	Cofilin_ADF	PF00241.21		Other biological process

NEMBOFW57_007982	31	6	24	6	27.6	6.16	100.13	6	x	x	x	x	x	x	DLH	PF01738.19		Other biological process
NEMBOFW57_007544	47	14	30	14	51.3	6.01	97.11	14		x	x	x	x		GDI	PF00996.19		Other biological process
NEMBOFW57_010231	38	23	49	23	70.9	5.21	95.36	23	x	x	x	x	x	x	HSP70	PF00012.21		Other biological process
NEMBOFW57_008011	55	17	52	17	42.4	7.49	95.03	17	x	x					Lactonase	PF10282.10		Other biological process
NEMBOFW57_005989	37	11	42	11	41.6	5.69	89.91	11	x	x	x	x	x	x	Actin	PF00022.20		Other biological process
NEMBOFW57_009987	23	7	21	7	62.9	6.71	83.8	7	x	x	x	x	x	x	PhoD	PF09423.11		Other biological process
NEMBOFW57_006536	47	9	23	8	28.7	7.36	67.55	9	x	x	x	x	x	x	DLH	PF01738.19		Other biological process
NEMBOFW57_008685	25	10	18	10	88.2	4.53	59.05	10		x	x	x		x	AMPK1_CBM	PF16561.6		Other biological process
NEMBOFW57_003962	31	5	16	5	25.5	6.61	57.48	5		x	x	x	x	x	Pro_CA	PF00484.20		Other biological process
NEMBOFW57_002974	35	7	16	7	41.3	6.7	56.86	7		x	x	x	x	x	Thiolase_N	PF00108.24		Other biological process
NEMBOFW57_007088	52	16	23	16	46.6	5.38	53.93	16	x	x					Amidase	PF01425.22		Other biological process
NEMBOFW57_009162	28	6	14	6	40.8	5.33	52.21	6	x	x	x	x	x	x	Lyase_1	PF00206.21		Other biological process
NEMBOFW57_001593	22	9	15	9	66.6	7.97	51.78	9	x	x	x	x		x	ATP-sulfurylase	PF01747.18		Other biological process
NEMBOFW57_001731	55	13	23	13	28.1	6.54	51.12	13	x	x	x	x	x	x	DLH	PF01738.19		Other biological process

NEMBOFW57_004626	35	8	21	8	46.4	5.05	46.79	8	x	x					AltA1	PF16541.6		Other biological process
NEMBOFW57_001267	21	9	14	9	75.8	6.8	46.61	9		x	x	x		x	TPP_enzyme_N	PF02776.19		Other biological process
NEMBOFW57_005969	12	8	15	8	107.6	5.35	41.8	8		x	x	x			CAS_CSE1	PF03378.16		Other biological process
NEMBOFW57_008905	32	9	13	9	43.5	7.27	40.98	9		x		x		x	Thiolase_N	PF00108.24		Other biological process
NEMBOFW57_003497	33	6	11	6	28.9	5.26	38.74	6			x	x	x	x	Hydrolase	PF00702.27	(S)-2-haloacid dehalogenase [Madurella mycetomatis]	Other biological process
NEMBOFW57_003346	18	3	9	3	23.7	5.45	38.19	3	x	x	x	x	x	x	GPI-anchored	PF10342.10		Other biological process
NEMBOFW57_001057	21	3	10	3	26.0	6.09	38.09	3		x	x	x	x	x	SnoaL_2	PF12680.8		Other biological process
NEMBOFW57_005781	43	3	10	3	14.5	6.27	37.78	3	x	x	x	x	x	x	Cerato-platanin	PF07249.13		Other biological process
NEMBOFW57_005231	23	4	11	4	29.5	4.93	36.93	4			x	x	x	x	HAD_2	PF13419.7		Other biological process
NEMBOFW57_000540	42	4	12	4	17.9	5.91	36.77	4		x	x	x	x	x	ARD	PF03079.15		Other biological process
NEMBOFW57_008393	28	16	20	16	72.2	5.03	36.48	16	x	x	x	x	x	x	HSP70	PF00012.21		Other biological process
NEMBOFW57_000009	30	4	12	4	15.7	7.05	35.71	4	x	x	x	x	x	x	MF_alpha_N	PF05436.12		Other biological process
NEMBOFW57_009639	24	6	13	6	51.3	7.72	35.28	6	x	x	x	x		x	NAD_binding_8	PF13450.7	Beta-cyclopiazonate dehydrogenase [Madurella mycetomatis]	Other biological process

NEMBOFW57_006372	26	6	12	6	35.2	8.02	34.6	6	x	x	x	x	x	x	NmrA	PF05368.14		Other biological process
NEMBOFW57_007379	18	6	15	6	40.4	6.44	33.44	6	x	x	x	x			Lactonase	PF10282.10		Other biological process
NEMBOFW57_000979	42	5	14	4	11.4	11.36	32.72	5	x	x		x			CENP-T_C	PF15511.7		Other biological process
NEMBOFW57_007841	18	4	12	4	32.1	5.5	32.51	4			x	x	x	x	Abhydrolase_1	PF00561.21	Dihydrolipoyllysine-residue acetyltransferase component of acetoin cleaving system [Madurella mycetomatis]	Other biological process
NEMBOFW57_008958	17	1	7	1	13.7	4.92	31.59	1	x	x	x	x	x	x	NTF2	PF02136.21		Other biological process
NEMBOFW57_002347	39	4	10	4	20.6	6.01	31.22	4	x	x	x	x	x	x	DMRL_synthase	PF00885.20		Other biological process
NEMBOFW57_009883	12	8	9	8	131.2	5.76	30.38	8	x	x	x	x		x	Sec16_C	PF12931.8		Other biological process
NEMBOFW57_000071	38	4	7	4	21.6	5.16	29.05	4	x		x	x	x	x	GPI-anchored	PF10342.10		Other biological process
NEMBOFW57_004791	31	10	16	10	42.5	6.24	28.75	10	x	x					PAN_4	PF14295.7		Other biological process
NEMBOFW57_004326	42	5	11	1	11.3	11.36	28.3	5		x					CENP-T_C	PF15511.7		Other biological process
NEMBOFW57_007942	24	7	9	7	43.9	7.31	27.85	7	x	x	x	x		x	Thiolase_N	PF00108.24		Other biological process
NEMBOFW57_002501	15	8	9	8	104.5	5.07	26.89	8		x		x		x	Het-C	PF07217.12		Other biological process
NEMBOFW57_010280	18	2	6	2	21.5	6.29	26.51	2	x	x	x	x	x	x	AltA1	PF16541.6		Other biological process

NEMBOFW57_002875	18	5	8	5	60.0	5.55	26.31	5				x		x	Amidohydro_3	PF07969.12		Other biological process
NEMBOFW57_001002	14	6	7	6	67.5	5.67	25.42	6		x	x	x			Creatinase_N_2	PF16189.6		Other biological process
NEMBOFW57_002880	24	7	9	7	43.6	9.16	25.13	7			x	x	x	x	PAP2	PF01569.22		Other biological process
NEMBOFW57_005449	12	4	9	4	39.9	5.1	25.0	4			x	x	x	x	polyprenyl_synt	PF00348.18		Other biological process
NEMBOFW57_001785	12	5	13	5	56.3	6.35	24.87	5	x	x					Fasciclin	PF02469.23		Other biological process
NEMBOFW57_006018	21	4	9	4	33.2	5.99	24.5	4		x	x	x	x	x	Spermine_synth	PF01564.18		Other biological process
NEMBOFW57_005488	20	4	9	4	34.9	7.39	24.07	4			x	x	x	x	Porphobil_deam	PF01379.21		Other biological process
NEMBOFW57_010263	36	12	14	12	45.9	5.64	23.12	12		x					Meth_synt_2	PF01717.19		Other biological process
NEMBOFW57_007284	10	4	6	4	72.5	8.47	22.07	4		x		x		x	Carn_acyltransf	PF00755.21		Other biological process
NEMBOFW57_006110	18	3	7	3	36.5	6.52	21.88	3			x	x			Amidohydro_1	PF01979.21		Other biological process
NEMBOFW57_003752	14	8	8	8	55.4	5.83	21.71	8	x	x					Beta-lactamase	PF00144.25		Other biological process
NEMBOFW57_006554	20	2	5	2	19.6	9.31	20.75	2	x			x		x	Inositol_P	PF00459.26		Other biological process
NEMBOFW57_000378	11	2	6	2	31.8	9.23	19.04	2				x		x	ECH_1	PF00378.21		Other biological process
NEMBOFW57_003047	19	5	7	5	32.6	5.44	18.84	5	x	x		x		x	Pyrophosphatase	PF00719.20		Other biological process

NEMBOFW57_009840	29	3	6	3	22.8	4.51	18.83	3		x	x	x		x	CS	PF04969.17		Other biological process
NEMBOFW57_010621	29	6	8	6	33.3	6.37	18.0	6	x	x					NmrA	PF05368.14		Other biological process
NEMBOFW57_000673	9	6	9	6	90.5	5.17	17.85	6		x				x	AAA	PF00004.30		Other biological process
NEMBOFW57_007637	30	4	4	4	34.5	5.08	17.01	4				x		x	PfkB	PF00294.25		Other biological process
NEMBOFW57_010469	29	3	8	2	14.2	10.55	16.77	3	x	x					Histone_H2A_C	PF16211.6		Other biological process
NEMBOFW57_009628	23	5	5	5	45.8	5.83	16.38	5		x	x	x		x	Globin	PF00042.23		Other biological process
NEMBOFW57_002503	9	5	6	5	90.1	6.16	16.35	5		x		x			Amidohydro_1	PF01979.21		Other biological process
NEMBOFW57_001068	26	5	8	4	28.9	8.0	16.18	5	x	x					DLH	PF01738.19		Other biological process
NEMBOFW57_005895	7	4	6	4	64.7	5.83	16.13	4		x		x			Metallophos	PF00149.29		Other biological process
NEMBOFW57_005040	10	3	5	3	58.7	5.9	16.12	3		x		x			NAD_binding_5	PF07994.13	Inositol-3-phosphate synthase [Madurella mycetomatis]	Other biological process
NEMBOFW57_007353	18	9	10	9	72.5	6.14	16.1	9	x	x		x	x		HSP70	PF00012.21		Other biological process
NEMBOFW57_005717	39	4	11	4	20.0	4.79	16.09	4	x	x		x	x		AltA1	PF16541.6		Other biological process
NEMBOFW57_001646	12	3	4	3	57.6	8.66	16.01	3		x	x	x		x	Lyase_1	PF00206.21		Other biological process
NEMBOFW57_000705	15	7	9	7	65.7	5.11	16.0	7	x	x		x	x	x	Alk_phosphatase	PF00245.21		Other biological process

NEMBOFW57_010500	9	4	5	4	63.5	6.6	15.94	4	x	x	x	x			TPP_enzyme_N	PF02776.19		Other biological process
NEMBOFW57_006975	23	8	8	8	59.9	7.65	15.79	8		x		x		x	SLAC1	PF03595.18		Other biological process
NEMBOFW57_004820	44	3	6	3	16.1	5.62	15.68	3	x	x	x	x	x	x	Opy2	PF09463.11		Other biological process
NEMBOFW57_006734	4	1	5	1	44.4	6.09	15.31	1			x	x		x	Beta-lactamase	PF00144.25		Other biological process
NEMBOFW57_010270	21	3	7	2	15.2	10.35	15.13	3	x	x					Histone	PF00125.25		Other biological process
NEMBOFW57_004725	13	5	8	5	38.9	6.32	14.93	5	x	x					Lactonase	PF10282.10		Other biological process
NEMBOFW57_003248	12	5	10	5	66.7	5.39	14.88	5	x	x		x			HSP70	PF00012.21		Other biological process
NEMBOFW57_005906	15	6	7	6	51.5	6.39	14.81	6	x	x					Questin_oxidase	PF14027.7		Other biological process
NEMBOFW57_004325	20	4	10	4	15.4	11.15	14.39	4	x	x					Histone	PF00125.25		Other biological process
NEMBOFW57_009145	2	6	10	6	313.8	7.46	14.32	6		x					Gcn1_N	PF12074.9		Other biological process
NEMBOFW57_009340	27	5	8	5	34.0	6.51	14.23	5	x	x					AltA1	PF16541.6		Other biological process
NEMBOFW57_007020	17	6	6	6	46.6	6.16	13.72	6		x					Cupin_1	PF00190.23		Other biological process
NEMBOFW57_009102	31	4	8	4	15.6	5.39	13.63	4	x	x	x	x	x	x	Hce2	PF14856.7		Other biological process
NEMBOFW57_005840	39	7	8	7	22.4	5.38	13.61	7	x						Nitroreductase	PF00881.25		Other biological process

NEMBOFW57_007484	24	3	4	3	14.1	6.15	13.52	3	x	x	x	x	x	x	Profilin	PF00235.20		Other biological process
NEMBOFW57_002660	8	3	6	3	40.7	5.55	13.45	3	x	x					Pil1	PF13805.7		Other biological process
NEMBOFW57_007040	39	5	7	5	18.1	8.15	13.43	5	x	x					Ricin_B_lectin	PF00652.23		Other biological process
NEMBOFW57_000113	10	3	4	3	66.8	5.73	13.36	3			x	x		x	Pro-kuma_activ	PF09286.12		Other biological process
NEMBOFW57_010230	36	4	4	4	27.8	6.37	12.87	4			x	x			DLH	PF01738.19		Other biological process
NEMBOFW57_007159	52	7	11	7	18.9	6.64	12.43	7		x					GFA	PF04828.15		Other biological process
NEMBOFW57_008405	5	1	3	1	33.5	6.46	12.41	1			x	x	x		PNP_UDP_1	PF01048.21		Other biological process
NEMBOFW57_004407	14	3	5	3	32.6	9.85	12.16	3	x	x					Bac_rhodopsin	PF01036.19		Other biological process
NEMBOFW57_004585	11	3	7	3	34.0	9.82	12.12	3	x	x					Mito_carr	PF00153.28		Other biological process
NEMBOFW57_008556	20	6	8	6	49.9	4.87	12.04	6	x	x	x		x		Tubulin	PF00091.26		Other biological process
NEMBOFW57_004578	11	4	6	4	50.0	5.19	11.91	4	x	x	x			x	Tubulin	PF00091.26		Other biological process
NEMBOFW57_010943	9	8	9	8	123.6	7.33	11.58	8	x	x					Tannase	PF07519.12		Other biological process
NEMBOFW57_000025	12	2	4	2	31.4	5.83	11.46	2		x	x	x	x	x	SurE	PF01975.18		Other biological process
NEMBOFW57_006163	4	1	3	1	30.5	5.52	11.06	1			x	x			Motile_Sperm	PF00635.27		Other biological process

NEMBOFW57_008366	6	2	5	2	43.9	6.05	10.99	2				x	x	x	Inositol_P	PF00459.26		Other biological process
NEMBOFW57_004068	28	8	8	8	32.0	5.54	10.88	8	x	x					NmrA	PF05368.14		Other biological process
NEMBOFW57_007655	11	4	5	4	81.4	8.35	10.22	4	x	x					CENP-F_N	PF10481.10		Other biological process
NEMBOFW57_003209	4	2	4	2	57.9	4.91	9.98	2		x	x	x	x	x	HSP70	PF00012.21		Other biological process
NEMBOFW57_006097	23	7	7	7	30.0	9.13	9.95	7		x					Porin_3	PF01459.23		Other biological process
NEMBOFW57_000769	25	2	2	2	28.9	4.92	9.64	2				x	x		Ran_BP1	PF00638.19		Other biological process
NEMBOFW57_001422	5	3	3	3	115.5	6.9	9.41	3			x	x			GDC-P	PF02347.17		Other biological process
NEMBOFW57_006710	8	2	3	2	45.1	5.49	9.38	2				x			Beta-lactamase	PF00144.25		Other biological process
NEMBOFW57_007758	13	3	3	3	41.9	5.57	9.24	3						x	Svf1_C	PF17187.5		Other biological process
NEMBOFW57_007859	11	3	3	3	40.2	6.4	9.22	3			x				2-Hacid_dh_C	PF02826.20		Other biological process
NEMBOFW57_001065	6	2	3	2	57.2	6.39	9.1	2				x		x	PALP	PF00291.26		Other biological process
NEMBOFW57_001419	11	1	2	1	33.1	5.39	8.9	1	x	x	x	x		x	CAP	PF00188.27		Other biological process
NEMBOFW57_008030	1	1	4	1	57.4	6.96	8.45	1	x	x					APH	PF01636.24		Other biological process
NEMBOFW57_010112	8	3	7	3	38.4	6.64	8.33	3		x					Band_7	PF01145.26		Other biological process

NEMBOFW57_005812	48	5	5	5	12.7	5.5	8.31	5	x	x		x	x	x	FKBP_C	PF00254.29		Other biological process
NEMBOFW57_010470	21	4	11	4	15.3	10.18	8.27	4	x	x	x	x	x	x	Histone	PF00125.25		Other biological process
NEMBOFW57_006808	14	5	5	5	47.5	8.16	8.18	5		x		x		x	Acyl-CoA_dh_1	PF00441.25		Other biological process
NEMBOFW57_006592	13	4	4	4	40.3	6.54	8.11	4		x					NMO	PF03060.16		Other biological process
NEMBOFW57_009532	12	2	6	1	21.1	4.75	7.77	2		x					AltA1	PF16541.6		Other biological process
NEMBOFW57_007559	11	8	9	8	85.7	5.1	7.75	8		x		x			HSP70	PF00012.21		Other biological process
NEMBOFW57_007262	9	2	2	2	44.6	8.6	7.71	2		x	x	x		x	Thiolase_N	PF00108.24		Other biological process
NEMBOFW57_000397	11	3	4	3	18.7	4.56	7.55	3	x	x		x			TCTP	PF00838.18		Other biological process
NEMBOFW57_004658	2	2	3	2	58.6	6.8	7.5	2	x	x					AcetylCoA_hydro	PF02550.16		Other biological process
NEMBOFW57_001734	5	2	3	2	61.3	8.87	7.27	2				x			Carboxyl_trans	PF01039.23		Other biological process
NEMBOFW57_004171	11	3	3	3	37.1	7.99	6.92	3				x			ECH_1	PF00378.21		Other biological process
NEMBOFW57_003345	8	2	3	2	44.7	5.08	6.88	2	x	x					Tubulin	PF00091.26		Other biological process
NEMBOFW57_002309	7	2	2	2	51.1	8.92	6.88	2				x			GCV_T	PF01571.22		Other biological process
NEMBOFW57_006449	2	4	4	4	167.0	6.44	6.88	4	x			x		x	NIR_SIR	PF01077.23		Other biological process

NEMBOFW57_007496	6	3	3	3	54.8	6.05	6.81	3				x			Pyridoxal_deC	PF00282.20		Other biological process
NEMBOFW57_006953	13	3	3	3	24.7	8.35	6.76	3		x					Cu_bind_like	PF02298.18		Other biological process
NEMBOFW57_007686	8	1	2	1	22.9	6.3	6.71	1				x			GLTP	PF08718.12		Other biological process
NEMBOFW57_003460	12	4	4	4	61.0	5.0	6.69	4		x					PH_6	PF15406.7		Other biological process
NEMBOFW57_010052	14	4	4	4	49.2	7.24	6.69	4		x					Thiolase_N	PF00108.24		Other biological process
NEMBOFW57_007452	2	1	3	1	54.6	7.33	6.65	1	x	x					Septin	PF00735.19		Other biological process
NEMBOFW57_006345	25	2	2	2	20.2	6.52	6.64	2	x						Cupin_5	PF06172.12		Other biological process
NEMBOFW57_002746	10	2	2	2	36.7	6.89	6.52	2			x	x			Carb_kinase	PF01256.18		Other biological process
NEMBOFW57_003353	25	6	6	6	33.8	8.57	6.44	6		x					MaoC_dehydratas	PF01575.20		Other biological process
NEMBOFW57_000020	8	2	2	2	34.8	5.43	6.4	2			x	x	x		Rhodanese	PF00581.21		Other biological process
NEMBOFW57_004933	58	5	7	5	14.8	6.98	6.36	5	x	x					GFA	PF04828.15		Other biological process
NEMBOFW57_010438	11	2	4	2	31.7	7.09	6.18	2		x					Gpr1_Fun34_YaaH	PF01184.20		Other biological process
NEMBOFW57_004875	15	2	2	2	21.9	5.17	6.07	2		x		x			CMD	PF02627.21		Other biological process
NEMBOFW57_002001	4	2	2	2	85.8	5.8	6.06	2				x			PUL	PF08324.12		Other biological process

NEMBOFW57_001125	9	2	2	2	43.5	6.89	5.95	2				x			TauD	PF02668.17		Other biological process
NEMBOFW57_005678	12	3	5	3	34.6	8.87	5.85	3	x	x					CoA_binding	PF02629.20		Other biological process
NEMBOFW57_008336	11	3	3	3	42.4	5.95	5.7	3		x					Beta-lactamase	PF00144.25		Other biological process
NEMBOFW57_001021	9	3	3	3	33.2	9.64	5.64	3		x					HABP4_PAI-RBP1	PF04774.16		Other biological process
NEMBOFW57_005962	3	1	2	1	65.4	7.52	5.64	1			x	x		x	G_glu_transpept	PF01019.22		Other biological process
NEMBOFW57_001612	4	2	2	2	53.7	5.86	5.44	2						x	HAD	PF12710.8		Other biological process
NEMBOFW57_006563	38	3	3	3	11.5	4.97	5.27	3	x	x	x	x	x		ACBP	PF00887.20		Other biological process
NEMBOFW57_001465	13	2	2	2	23.5	4.88	5.26	2		x					SGL	PF08450.13		Other biological process
NEMBOFW57_010199	8	2	2	2	29.5	6.8	5.25	2				x		x	ECH_1	PF00378.21		Other biological process
NEMBOFW57_008256	6	2	2	2	105.7	4.64	5.04	2		x					Ad_cyc_g-alpha	PF08509.12		Other biological process
NEMBOFW57_000933	16	3	3	3	38.2	5.3	4.99	3	x	x					PfkB	PF00294.25		Other biological process
NEMBOFW57_003065	13	5	5	5	63.3	5.14	4.95	5		x					Calreticulin	PF00262.19		Other biological process
NEMBOFW57_009414	7	2	2	2	54.2	7.25	4.94	2				x		x	TauD	PF02668.17		Other biological process
NEMBOFW57_009916	3	2	2	2	106.5	5.33	4.89	2		x					CRM1_C	PF08767.12		Other biological process

NEMBOFW57_006301	11	2	2	2	17.0	6.14	4.79	2		x		x			Rhodanese	PF00581.21		Other biological process
NEMBOFW57_008417	12	4	4	4	55.1	7.01	4.63	4		x					PH	PF00169.30		Other biological process
NEMBOFW57_005662	4	1	2	1	33.8	7.81	4.62	1		x					ECH_1	PF00378.21		Other biological process
NEMBOFW57_003535	16	3	3	3	34.2	5.53	4.57	3		x					Methyltransf_25	PF13649.7		Other biological process
NEMBOFW57_004499	23	6	6	6	44.1	5.45	4.55	6		x					Beta-lactamase	PF00144.25		Other biological process
NEMBOFW57_005563	11	2	2	2	17.6	9.31	4.39	2	x	x					PBP	PF01161.21		Other biological process
NEMBOFW57_004967	6	1	1	1	31.3	8.62	4.38	1			x	x			Flocculin_t3	PF13928.7		Other biological process
NEMBOFW57_005129	6	2	2	2	80.7	6.28	4.36	2				x			CN_hydrolase	PF00795.23		Other biological process
NEMBOFW57_009679	4	3	3	3	103.0	6.83	4.29	3		x					Amidohydro_3	PF07969.12		Other biological process
NEMBOFW57_000965	1	1	2	1	102.3	5.82	4.22	1		x		x	x		AAA_2	PF07724.15		Other biological process
NEMBOFW57_004764	5	1	1	1	66.9	6.47	4.17	1			x	x			FAD_binding_4	PF01565.24	6-hydroxy-D-nicotine oxidase [Madurella mycetomatis]	Other biological process
NEMBOFW57_002235	13	3	3	3	44.2	6.16	4.11	3	x			x			S-AdoMet_synt_C	PF02773.17		Other biological process
NEMBOFW57_006477	10	3	3	2	39.5	5.96	4.05	3		x					Pkinase	PF00069.26		Other biological process
NEMBOFW57_005479	27	3	3	3	18.8	4.96	3.99	3		x					Tropomyosin_1	PF12718.8		Other biological process

NEMBOFW57_001149	6	2	3	2	35.3	9.89	3.98	2		x					Mito_carr	PF00153.28		Other biological process
NEMBOFW57_004306	3	1	2	1	32.3	9.31	3.94	1	x	x					Mito_carr	PF00153.28		Other biological process
NEMBOFW57_000742	2	1	1	1	93.2	6.04	3.9	1					x		Vps16_N	PF04841.14		Other biological process
NEMBOFW57_008837	12	2	2	2	19.1	8.97	3.85	2		x					RITA	PF17066.6		Other biological process
NEMBOFW57_003903	7	1	1	1	35.3	5.87	3.85	1				x		x	Thi4	PF01946.18		Other biological process
NEMBOFW57_009879	3	1	1	1	57.7	6.2	3.74	1				x			Amino_oxidase	PF01593.25		Other biological process
NEMBOFW57_008873	4	4	4	4	131.8	5.08	3.73	4		x		x			Crescentin	PF19220.1		Other biological process
NEMBOFW57_002901	13	1	1	1	22.0	11.6	3.69	1					x		zf-RING_2	PF13639.7		Other biological process
NEMBOFW57_005874	11	1	1	1	17.0	4.27	3.64	1		x		x			EF-hand_1	PF00036.33		Other biological process
NEMBOFW57_005318	3	2	2	2	71.9	7.21	3.61	2	x	x					Alk_phosphatase	PF00245.21		Other biological process
NEMBOFW57_001361	10	1	1	1	20.6	5.9	3.6	1				x			Haem_degrading	PF03928.15		Other biological process
NEMBOFW57_001857	14	2	2	2	21.8	4.86	3.57	2		x				x	NAC	PF01849.19		Other biological process
NEMBOFW57_008631	3	2	2	2	72.5	6.09	3.54	2				x			CH	PF00307.32		Other biological process
NEMBOFW57_006619	8	1	1	1	33.0	7.64	3.51	1				x			PHP	PF02811.20		Other biological process

NEMBOFW57_005451	7	2	2	2	34.4	6.33	3.5	2		x					RmlD_sub_bind	PF04321.18		Other biological process
NEMBOFW57_002461	5	1	1	1	34.9	7.37	3.5	1				x			CHORD	PF04968.13		Other biological process
NEMBOFW57_006184	5	2	2	1	46.3	5.63	3.49	2		x					Pkinase	PF00069.26		Other biological process
NEMBOFW57_000340	26	5	6	5	35.3	7.5	3.45	5	x	x					Fasciclin	PF02469.23		Other biological process
NEMBOFW57_003792	3	1	1	1	58.0	5.33	3.42	1				x			Amidohydro_1	PF01979.21		Other biological process
NEMBOFW57_006141	8	2	3	2	30.5	9.23	3.41	2	x	x					Band_7	PF01145.26		Other biological process
NEMBOFW57_004378	7	1	1	1	33.7	7.72	3.41	1						x	Cu_bind_like	PF02298.18		Other biological process
NEMBOFW57_003009	3	1	1	1	48.3	7.24	3.39	1					x		COPIIcoated_ERV	PF07970.13		Other biological process
NEMBOFW57_008466	7	3	3	3	44.2	6.74	3.32	3		x					ESCRT-II	PF05871.13		Other biological process
NEMBOFW57_010227	0	1	3	1	273.5	4.81	3.3	1	x						CRT-like	PF08627.11		Other biological process
NEMBOFW57_004128	3	1	1	1	48.5	7.42	3.25	1			x				AAA	PF00004.30		Other biological process
NEMBOFW57_010267	14	2	4	2	22.1	9.38	3.23	2	x	x					CDH-cyt	PF16010.6		Other biological process
NEMBOFW57_007746	3	1	1	1	74.7	9.14	3.18	1				x			TPP_enzyme_N	PF02776.19		Other biological process
NEMBOFW57_008173	10	1	1	1	20.6	5.58	3.16	1	x		x				WW_like	PF17890.2		Other biological process

NEMBOFW57_000479	5	1	1	1	33.4	5.54	3.11	1				x		x	Hydrolase_6	PF13344.7		Other biological process
NEMBOFW57_003005	2	1	1	1	145.9	5.25	3.01	1				x			NUP214	PF16755.6		Other biological process
NEMBOFW57_006864	20	1	1	1	16.1	5.5	3.0	1				x		x	CoA_binding_2	PF13380.7		Other biological process
NEMBOFW57_008342	1	1	1	1	140.7	5.29	2.96	1				x			NPC1_N	PF16414.6		Other biological process
NEMBOFW57_002843	7	1	1	1	36.4	7.81	2.88	1				x			ECH_1	PF00378.21		Other biological process
NEMBOFW57_002203	15	1	1	1	10.1	8.5	2.84	1			x				Herpes_UL56	PF04534.13		Other biological process
NEMBOFW57_002121	3	1	1	1	36.7	7.34	2.84	1				x			P34-Arc	PF04045.15		Other biological process
NEMBOFW57_005053	7	1	1	1	30.6	7.06	2.74	1		x		x			ECH_1	PF00378.21		Other biological process
NEMBOFW57_002761	1	1	1	1	294.2	6.3	2.71	1			x				DRIM	PF07539.13		Other biological process
NEMBOFW57_005020	3	1	1	1	57.1	5.63	2.69	1				x			Kelch_3	PF13415.7		Other biological process
NEMBOFW57_006310	4	1	1	1	36.5	6.23	2.68	1		x					Amidohydro_2	PF04909.15		Other biological process
NEMBOFW57_002680	1	1	1	1	94.3	8.57	2.6	1				x			VTC	PF09359.11		Other biological process
NEMBOFW57_002752	1	1	1	1	190.1	5.39	2.44	1				x			Clathrin	PF00637.21		Other biological process
NEMBOFW57_008991	4	1	1	1	33.5	6.68	2.4	1				x			WD40	PF00400.33	Putative transporter [Podospora comata]	Other biological process

NEMBOFW57_005790	6	1	1	1	18.5	4.28	2.38	1	x	x					CFEM	PF05730.12		Other biological process
NEMBOFW57_001897	6	2	2	2	32.7	6.24	2.38	2	x	x					QRPTase_C	PF01729.20		Other biological process
NEMBOFW57_001387	11	2	2	2	22.3	5.03	2.36	2		x					Cupin_2	PF07883.12		Other biological process
NEMBOFW57_000465	3	1	1	1	39.8	6.57	2.31	1		x					KH_1	PF00013.30		Other biological process
NEMBOFW57_010598	10	2	2	2	28.9	6.09	2.31	2	x	x				x	NPP1	PF05630.12		Other biological process
NEMBOFW57_007552	5	1	1	1	22.4	9.47	2.25	1				x			Rhodanese	PF00581.21		Other biological process
NEMBOFW57_002674	3	1	1	1	36.4	6.37	2.23	1		x					Pil1	PF13805.7		Other biological process
NEMBOFW57_005575	1	1	1	1	195.2	6.27	2.23	1				x			ABC_tran	PF00005.28		Other biological process
NEMBOFW57_003921	13	1	1	1	15.1	6.73	2.2	1		x					HIT	PF01230.24		Other biological process
NEMBOFW57_007843	15	1	1	1	8.3	11.55	2.18	1		x							putative transcriptional repressor protein [Chaetomium thermophilum var. thermophilum DSM 1495]	Other biological process
NEMBOFW57_004268	3	1	1	1	43.0	5.62	2.17	1		x					Meth_synt_2	PF01717.19		Other biological process
NEMBOFW57_005795	1	1	1	1	55.2	7.53	2.17	1				x			TPR_2	PF07719.18		Other biological process
NEMBOFW57_002489	3	1	1	1	86.2	7.3	2.13	1		x					TFR_dimer	PF04253.16		Other biological process

NEMBOFW57_000814	2	1	2	1	58.0	5.31	2.1	1		x					Cpn60_TCP1	PF00118.25		Other biological process
NEMBOFW57_005189	35	4	4	4	19.6	5.4	2.09	4		x					Cyanate_lyase	PF02560.15		Other biological process
NEMBOFW57_010544	1	1	1	1	125.0	4.88	2.08	1				x			Importin_rep_6	PF18829.2		Other biological process
NEMBOFW57_008305	1	1	1	1	53.9	9.1	1.99	1	x	x					UQ_con	PF00179.27		Other biological process
NEMBOFW57_003663	19	3	3	3	23.0	7.56	1.98	3		x					PMSR	PF01625.22		Other biological process
NEMBOFW57_009326	4	1	1	1	20.4	5.22	1.89	1	x						Lipocalin_2	PF08212.13		Other biological process
NEMBOFW57_006193	8	3	3	3	56.7	5.57	1.89	3	x	x					Spherulin4	PF12138.9		Other biological process
NEMBOFW57_008481	3	1	1	1	39.9	7.55	1.86	1	x						NTP_transferase	PF00483.24		Other biological process
NEMBOFW57_003920	16	3	4	3	22.0	4.51	1.84	3		x					Mog1	PF04603.13		Other biological process
NEMBOFW57_003986	3	1	1	1	37.5	5.97	1.82	1		x					CN_hydrolase	PF00795.23		Other biological process
NEMBOFW57_004943	8	2	2	2	31.3	6.54	1.81	2		x					HAD_2	PF13419.7		Other biological process
NEMBOFW57_001542	5	1	1	1	19.9	6.7	1.81	1		x					ARPC4	PF05856.13		Other biological process
NEMBOFW57_005972	3	1	1	1	32.9	6.06	1.81	1				x			ABC_tran	PF00005.28		Other biological process
NEMBOFW57_006369	5	2	2	2	43.8	6.06	1.79	2		x					Actin	PF00022.20		Other biological process

NEMBOFW57_007827	3	1	1	1	29.3	5.14	1.79	1		x					Methyltransf_23	PF13489.7		Other biological process
NEMBOFW57_007197	1	1	1	1	106.7	5.41	1.78	1				x			Adaptin_N	PF01602.21		Other biological process
NEMBOFW57_000112	4	1	1	1	27.4	5.62	1.74	1		x					Methyltransf_24	PF13578.7		Other biological process
NEMBOFW57_003422	2	1	2	1	55.8	9.5	1.72	1		x					RabGAP-TBC	PF00566.19		Other biological process
NEMBOFW57_002551	7	2	2	2	28.4	4.64	1.71	2		x					PCNA_N	PF00705.19		Other biological process
NEMBOFW57_005120	1	1	1	1	64.5	5.11	1.71	1		x					ERM	PF00769.20		Other biological process
NEMBOFW57_009017	14	2	2	2	19.9	6.92	1.7	2	x						DEC-1_N	PF04625.14		Other biological process
NEMBOFW57_004279	9	1	1	1	15.3	6.73	1.7	1		x					zf-nanos	PF05741.14		Other biological process
NEMBOFW57_002709	2	1	2	1	40.8	9.32	1.7	1	x	x					Mito_carr	PF00153.28		Other biological process
NEMBOFW57_009228	6	1	1	1	12.1	4.3	1.7	1		x					CVNH	PF08881.11		Other biological process
NEMBOFW57_002172	3	1	1	1	25.7	6.28	1.7	1		x					Ank_2	PF12796.8		Other biological process
NEMBOFW57_003202	8	1	2	1	21.1	6.05	1.66	1	x	x					Arf	PF00025.22		Other biological process
NEMBOFW57_003591	1	1	1	1	51.1	5.71	1.65	1		x					Ferritin_2	PF13668.7		Other biological process
NEMBOFW57_008334	4	1	1	1	28.5	6.73	1.65	1				x			CN_hydrolase	PF00795.23		Other biological process

NEMBOFW57_000668	1	1	1	1	140.7	5.6	1.63	1		x					CLU	PF13236.7		Other biological process
NEMBOFW57_007257	3	1	2	1	37.3	9.6	1.61	1		x					FYVE_2	PF02318.17		Other biological process
NEMBOFW57_007602	6	1	1	1	19.4	4.94	1.61	1	x	x					E1_DerP2_DerF2	PF02221.16		Other biological process
NEMBOFW57_010262	2	1	1	1	59.4	6.29	1.6	1		x					Abhydrolase_1	PF00561.21	3-oxoadipate enol-lactonase 2 [Madurella mycetomatis]	Other biological process
NEMBOFW57_005395	3	1	1	1	45.6	4.45	1.6	1		x					NAP	PF00956.19		Other biological process
NEMBOFW57_010071	11	1	1	1	10.0	4.97	0.0	1	x						HMA	PF00403.27		Other biological process
NEMBOFW57_005745	9	1	1	1	30.4	5.78	0.0	1	x						Spherulin4	PF12138.9		Other biological process
NEMBOFW57_003716	3	1	1	1	47.2	6.47	0.0	1	x						Phosphoesterase	PF04185.15		Other biological process
NEMBOFW57_004070	1	1	1	1	82.7	6.28	0.0	1	x						HET	PF06985.12		Other biological process
NEMBOFW57_009237	5	1	2	1	61.1	8.54	0.0	1	x						Sugar_tr	PF00083.25		Other biological process
NEMBOFW57_006217	24	1	3	1	13.1	4.7	0.0	1	x						Herpes_LMP1	PF05297.12		Other biological process
NEMBOFW57_002587	3	1	1	1	160.7	7.39	0.0	1	x						F-box-like	PF12937.8		Other biological process
NEMBOFW57_006699	2	1	1	1	80.1	5.26	0.0	1	x						Phytase	PF02333.16		Other biological process
NEMBOFW57_009888	7	1	2	1	18.6	9.73	0.0	1		x					S10_plectin	PF03501.16		Other biological process

NEMBOFW57_007069	5	1	1	1	30.6	9.94	0.0	1		x					TMEM33_Pom33	PF03661.14		Other biological process
NEMBOFW57_009341	6	3	3	3	42.9	5.07	0.0	3		x					Methyltransf_23	PF13489.7		Other biological process
NEMBOFW57_010939	16	2	2	2	19.7	5.38	0.0	2		x					Cupin_2	PF07883.12		Other biological process
NEMBOFW57_001895	1	1	1	1	95.6	8.35	0.0	1		x					NAT	PF04768.14		Other biological process
NEMBOFW57_007694	2	2	2	2	117.0	6.18	0.0	2		x					ABC_tran	PF00005.28		Other biological process
NEMBOFW57_006702	17	1	1	1	10.5	7.4	0.0	1		x					Chitin_bind_1	PF00187.20		Other biological process
NEMBOFW57_001928	3	1	1	1	27.6	10.46	0.0	1		x					SUR7	PF06687.13		Other biological process
NEMBOFW57_005150	4	1	1	1	52.8	8.73	0.0	1		x					DKCLD	PF08068.13		Other biological process
NEMBOFW57_004324	6	1	1	1	24.8	7.46	0.0	1		x					DSBA	PF01323.21		Other biological process
NEMBOFW57_008678	2	2	2	2	100.3	7.23	0.0	2		x					FTHFS	PF01268.20		Other biological process
NEMBOFW57_000091	4	1	1	1	35.4	5.59	0.0	1		x					PhzC-PhzF	PF02567.17		Other biological process
NEMBOFW57_008381	3	1	1	1	75.2	6.99	0.0	1		x					AMP-binding	PF00501.29		Other biological process
NEMBOFW57_009769	8	1	1	1	19.2	4.77	0.0	1		x					NuiA	PF07924.12		Other biological process
NEMBOFW57_008363	2	1	1	1	41.1	6.33	0.0	1		x					Lactonase	PF10282.10		Other biological process

NEMBOFW57_002896	14	1	1	1	12.2	6.55	0.0	1	x	x					ABM	PF03992.17		Other biological process
NEMBOFW57_004502	3	1	1	1	39.8	6.3	0.0	1		x					HMGL-like	PF00682.20		Other biological process
NEMBOFW57_005261	5	1	2	1	16.7	4.89	0.0	1		x					PGA2	PF07543.13		Other biological process
NEMBOFW57_006211	1	1	1	1	99.8	6.65	0.0	1		x					PPDK_N	PF01326.20		Other biological process
NEMBOFW57_007664	3	1	2	1	44.5	5.08	0.0	1		x					DSPc	PF00782.21		Other biological process
NEMBOFW57_008319	1	1	1	1	136.4	6.37	0.0	1	x	x					NAD_binding_4	PF07993.13	male sterility protein [Colleto-trichum tofieldiae]	Other biological process
NEMBOFW57_004493	1	1	1	1	63.0	6.49	0.0	1		x					Pyridoxal_deC	PF00282.20		Other biological process
NEMBOFW57_009166	1	1	1	1	85.7	5.11	0.0	1		x					UCH	PF00443.30		Other biological process
NEMBOFW57_006075	4	1	1	1	36.6	6.92	0.0	1		x					Syntaxin	PF00804.26		Other biological process
NEMBOFW57_006834	9	1	1	1	21.6	5.49	0.0	1		x					Rho_GDI	PF02115.18		Other biological process
NEMBOFW57_009803	2	1	1	1	43.7	6.05	0.0	1		x					ATP-grasp_2	PF08442.11		Other biological process
NEMBOFW57_004154	2	1	1	1	49.2	5.36	0.0	1		x					Kelch_6	PF13964.7		Other biological process
NEMBOFW57_006053	4	1	1	1	35.9	4.88	0.0	1		x					Methyltransf_23	PF13489.7		Other biological process
NEMBOFW57_006841	1	1	1	1	114.1	4.81	0.0	1		x					IBN_N	PF03810.20		Other biological process

NEMBOFW57_002887	4	1	1	1	30.7	5.96	0.0	1		x					Hemerythrin	PF01814.24		Other biological process
NEMBOFW57_000642	22	17	44	17	116.7	5.85	174.38	17	x	x	x	x	x	x	Catalase	PF00199.20		Oxidation-reduction
NEMBOFW57_002578	43	18	37	18	55.1	4.86	146.09	18	x	x	x	x	x	x	Thioredoxin	PF00085.21		Oxidation-reduction
NEMBOFW57_007517	35	11	40	11	51.7	5.71	143.42	11	x	x	x	x	x	x	NAD_binding_8	PF13450.7		Oxidation-reduction
NEMBOFW57_005608	29	15	43	15	65.9	7.31	137.36	15	x	x	x	x	x	x	FAD_binding_4	PF01565.24		Oxidation-reduction
NEMBOFW57_005888	47	14	32	14	46.9	8.6	129.8	14	x	x	x	x	x	x	2-Hacid_dh_C	PF02826.20		Oxidation-reduction
NEMBOFW57_008312	28	11	26	11	64.5	8.03	78.43	11	x	x	x	x	x	x	FAD_binding_4	PF01565.24		Oxidation-reduction
NEMBOFW57_010142	49	18	36	18	47.0	6.32	78.23	18	x	x	x	x		x	Aldedh	PF00171.23		Oxidation-reduction
NEMBOFW57_007830	27	5	19	5	35.8	6.46	74.84	5			x	x	x	x	Aldo_ket_red	PF00248.22		Oxidation-reduction
NEMBOFW57_000013	33	20	36	20	84.4	6.07	71.58	20	x	x	x	x		x	peroxidase	PF00141.24		Oxidation-reduction
NEMBOFW57_000054	23	7	20	7	55.4	6.8	68.72	7	x		x	x	x	x	FAD_binding_4	PF01565.24		Oxidation-reduction
NEMBOFW57_009779	34	8	14	8	37.6	6.86	48.31	8		x	x	x			ADH_N	PF08240.13		Oxidation-reduction
NEMBOFW57_000548	24	7	12	7	51.9	5.49	45.97	7			x	x	x	x	GSH_synth_ATP	PF03917.18		Oxidation-reduction
NEMBOFW57_005744	27	3	8	3	26.7	6.3	40.21	3	x	x	x	x	x	x	Sod_Fe_C	PF02777.19		Oxidation-reduction

NEMBOFW57_005220	32	7	12	7	28.1	6.47	38.86	7		x	x	x	x	x	HAGH_C	PF16123.6		Oxidation-reduction
NEMBOFW57_007667	36	15	17	15	68.3	7.59	37.67	15	x	x	x				FAD_binding_4	PF01565.24		Oxidation-reduction
NEMBOFW57_009958	23	6	13	6	46.3	8.25	37.32	6		x	x	x		x	Coprogen_oxidas	PF01218.19		Oxidation-reduction
NEMBOFW57_010407	42	8	14	8	25.0	5.88	35.86	8	x	x	x	x	x	x	AhpC-TSA	PF00578.22		Oxidation-reduction
NEMBOFW57_001747	48	6	17	6	15.9	6.52	35.35	6	x	x	x	x	x	x	Sod_Cu	PF00080.21		Oxidation-reduction
NEMBOFW57_006888	25	5	9	5	24.5	7.81	34.77	5	x	x	x	x	x	x	Rieske	PF00355.27		Oxidation-reduction
NEMBOFW57_010695	45	10	10	10	38.1	7.5	32.5	10		x		x			ADH_zinc_N	PF00107.27	NADP-dependent alkenal double bond reductase P2 [Madurella mycetomatis]	Oxidation-reduction
NEMBOFW57_006984	36	5	9	5	33.0	6.33	32.19	5		x	x	x		x	Aldo_ket_red	PF00248.22		Oxidation-reduction
NEMBOFW57_008947	19	4	10	4	46.5	5.1	31.26	4				x		x	DAO	PF01266.25		Oxidation-reduction
NEMBOFW57_003365	29	8	15	8	36.3	5.9	31.18	8	x	x					Aldo_ket_red	PF00248.22		Oxidation-reduction
NEMBOFW57_010439	16	4	8	4	51.8	6.13	29.67	4		x	x	x		x	FAD_binding_2	PF00890.25	fumarate reductase-like protein [Chaetomium thermophilum var. thermophilum DSM 1495]	Oxidation-reduction
NEMBOFW57_001246	18	10	12	10	81.9	6.42	28.5	10	x	x	x	x		x	Catalase	PF00199.20		Oxidation-reduction
NEMBOFW57_002966	30	13	21	13	66.3	7.12	28.3	13	x	x	x	x		x	FAD_binding_2	PF00890.25	Fumarate reductase [Madurella mycetomatis]	Oxidation-reduction

NEMBOFW57_006495	22	13	13	13	95.7	8.38	28.21	13		x					adh_short	PF00106.26		Oxidation-reduction
NEMBOFW57_002570	24	6	11	6	29.2	5.78	27.37	6	x	x	x	x	x		Sod_Cu	PF00080.21		Oxidation-reduction
NEMBOFW57_002948	25	4	12	4	24.3	6.64	25.77	4	x	x	x	x	x	x	Thioredoxin_4	PF13462.7		Oxidation-reduction
NEMBOFW57_003990	35	8	9	8	28.4	7.61	25.08	8	x	x		x		x	NAD_binding_10	PF13460.7	NAD dependent epimerase/de-hydratase [Coniochaeta ligniaria NRRL 30616]	Oxidation-reduction
NEMBOFW57_008796	32	7	7	7	46.1	6.81	24.35	7			x	x			Oxidored_FMN	PF00724.21	Putative NADPH dehydrogenase [Podospora comata]	Oxidation-reduction
NEMBOFW57_003641	23	7	8	7	39.3	7.21	21.42	7	x	x	x	x			Aldo_ket_red	PF00248.22		Oxidation-reduction
NEMBOFW57_009620	28	5	6	5	25.1	7.75	18.92	5	x	x	x	x		x	Sod_Fe_C	PF02777.19		Oxidation-reduction
NEMBOFW57_007624	20	6	9	6	39.8	6.96	18.07	6		x	x	x			GST_N_2	PF13409.7		Oxidation-reduction
NEMBOFW57_008180	20	4	5	4	30.5	7.59	17.84	4	x	x					Dioxygenase_C	PF00775.22		Oxidation-reduction
NEMBOFW57_005754	14	3	5	3	40.3	6.11	17.75	3	x	x	x	x	x	x	GFO_IDH_MocA	PF01408.23		Oxidation-reduction
NEMBOFW57_005057	23	8	10	8	40.3	6.54	17.02	8		x					ADH_N	PF08240.13		Oxidation-reduction
NEMBOFW57_003548	23	7	11	7	40.1	6.68	16.81	7	x	x					ADH_N	PF08240.13		Oxidation-reduction
NEMBOFW57_002877	21	3	5	3	25.9	8.05	16.33	3			x	x	x		adh_short_C2	PF13561.7		Oxidation-reduction

NEMBOFW57_000149	16	4	7	4	29.1	5.72	15.56	4	x	x					Aldo_ket_red	PF00248.22		Oxidation-reduction
NEMBOFW57_005823	11	4	5	4	61.4	7.14	15.21	4	x		x	x	x	x	FAD_binding_4	PF01565.24	Putative isoamyl alcohol oxidase [Podospora comata]	Oxidation-reduction
NEMBOFW57_001470	20	3	5	3	30.3	6.24	12.86	3				x			adh_short_C2	PF13561.7		Oxidation-reduction
NEMBOFW57_001478	39	7	7	7	25.6	6.4	12.44	7	x	x				x	GST_N_3	PF13417.7		Oxidation-reduction
NEMBOFW57_001631	12	2	4	2	29.9	6.76	12.14	2				x			LigB	PF02900.19		Oxidation-reduction
NEMBOFW57_002963	14	3	3	3	41.1	4.94	12.03	3				x			2OG-FeII_Oxy	PF03171.21		Oxidation-reduction
NEMBOFW57_002363	21	8	8	8	59.5	5.43	11.57	8		x		x			PGM_PMM_I	PF02878.17		Oxidation-reduction
NEMBOFW57_009431	15	2	3	2	24.2	5.3	11.14	2	x	x		x			GST_N	PF02798.21		Oxidation-reduction
NEMBOFW57_002652	15	3	3	3	41.8	6.33	10.46	3			x	x			Oxidored_FMN	PF00724.21	NADH:flavin oxidoreductase/NADH oxidase-like protein	Oxidation-reduction
NEMBOFW57_003198	8	5	6	5	74.6	6.57	9.04	5	x	x		x		x	FAD_binding_2	PF00890.25	putative succinate dehydrogenase [ubiquinone] flavoprotein subunit, mitochondrial [Neonectria ditissima]	Oxidation-reduction
NEMBOFW57_009567	6	2	7	1	25.7	6.86	8.76	2	x	x					adh_short_C2	PF13561.7		Oxidation-reduction
NEMBOFW57_000406	8	3	3	3	60.8	6.19	8.7	3				x		x	FAD-oxidase_C	PF02913.20	D-lactate dehydrogenase [cytochrome] 2, mitochondrial [Madurella mycetomatis]	Oxidation-reduction

NEMBOFW57_000150	43	2	2	2	11.2	4.75	8.07	2		x		x		x	Thioredoxin	PF00085.21		Oxidation-reduction
NEMBOFW57_010751	11	3	7	2	27.8	7.88	8.02	3	x	x					adh_short_C2	PF13561.7		Oxidation-reduction
NEMBOFW57_007194	17	5	5	5	46.6	5.1	7.98	5		x					NAD_binding_11	PF14833.7	3-hydroxyisobutyrate dehydrogenase [Neurospora crassa OR74A]	Oxidation-reduction
NEMBOFW57_002856	11	5	6	5	53.3	5.71	7.86	5		x		x	x		Thioredoxin	PF00085.21		Oxidation-reduction
NEMBOFW57_000410	9	4	7	4	45.6	8.15	7.72	4	x	x					E1_dh	PF00676.21		Oxidation-reduction
NEMBOFW57_010467	9	3	7	3	46.2	6.86	7.72	3	x	x					2-oxoacid_dh	PF00198.24		Oxidation-reduction
NEMBOFW57_002432	9	3	3	3	48.5	5.97	7.65	3			x	x			DUF3500	PF12006.9		Oxidation-reduction
NEMBOFW57_004388	14	5	5	5	42.8	5.59	7.45	5		x					GFO_IDH_MocA	PF01408.23		Oxidation-reduction
NEMBOFW57_002978	13	3	3	3	31.9	8.78	7.36	3	x	x		x		x	Fer2_3	PF13085.7		Oxidation-reduction
NEMBOFW57_000660	13	3	3	3	35.1	6.43	7.35	3		x		x			ADH_N	PF08240.13		Oxidation-reduction
NEMBOFW57_008741	21	6	6	6	40.7	7.97	7.33	6		x		x			Aldo_ket_red	PF00248.22		Oxidation-reduction
NEMBOFW57_006666	20	6	6	6	35.3	7.61	7.32	6		x					ADH_zinc_N	PF00107.27		Oxidation-reduction
NEMBOFW57_008184	20	5	5	5	30.5	5.49	7.27	5	x	x		x			NAD_binding_10	PF13460.7	NAD(P)H azoreductase [Madurella mycetomatis]	Oxidation-reduction

NEMBOFW57_007647	11	3	3	3	32.8	9.38	7.13	3		x					adh_short_C2	PF13561.7		Oxidation-reduction
NEMBOFW57_008362	20	4	5	4	33.7	6.04	6.98	4		x					Dioxygenase_C	PF00775.22		Oxidation-reduction
NEMBOFW57_003796	5	1	2	1	46.9	8.29	6.84	1				x			Acyl-CoA_dh_N	PF02771.17		Oxidation-reduction
NEMBOFW57_003066	10	3	3	3	58.3	7.47	6.33	3		x	x	x		x	Aldedh	PF00171.23		Oxidation-reduction
NEMBOFW57_005837	12	3	3	3	29.1	8.6	6.23	3		x					adh_short_C2	PF13561.7		Oxidation-reduction
NEMBOFW57_006331	10	4	4	4	41.8	6.16	6.23	4		x		x			Aldo_ket_red	PF00248.22		Oxidation-reduction
NEMBOFW57_008812	6	2	2	2	76.5	6.33	6.22	2			x	x			Cu_amine_oxid	PF01179.21		Oxidation-reduction
NEMBOFW57_005822	18	4	4	4	28.7	7.18	6.15	4		x					GST_N_3	PF13417.7		Oxidation-reduction
NEMBOFW57_008061	13	4	4	4	37.7	5.5	6.07	4		x					GFO_IDH_MocA	PF01408.23		Oxidation-reduction
NEMBOFW57_004923	6	2	2	2	47.3	6.13	5.66	2				x		x	Aldedh	PF00171.23		Oxidation-reduction
NEMBOFW57_005347	5	1	2	1	33.5	8.62	5.22	1			x	x			3HCDH_N	PF02737.19		Oxidation-reduction
NEMBOFW57_010893	14	1	1	1	24.7	7.77	4.85	1			x				Peroxidase_2	PF01328.18		Oxidation-reduction
NEMBOFW57_006552	5	1	1	1	47.5	5.74	4.66	1			x				Dioxygenase_C	PF00775.22		Oxidation-reduction
NEMBOFW57_001628	4	1	1	1	45.1	6.01	4.36	1				x		x	DAO	PF01266.25		Oxidation-reduction

NEMBOFW57_010752	5	2	2	2	43.8	7.49	4.19	2		x					Aldo_ket_red	PF00248.22		Oxidation-reduction
NEMBOFW57_007852	2	1	2	1	46.2	6.61	4.06	1	x	x					2-oxoacid_dh	PF00198.24		Oxidation-reduction
NEMBOFW57_007971	9	3	3	3	42.7	6.55	4.03	3		x					GFO_IDH_MocA	PF01408.23		Oxidation-reduction
NEMBOFW57_002450	8	4	4	4	64.4	8.57	4.02	4		x	x				Aldedh	PF00171.23		Oxidation-reduction
NEMBOFW57_005394	3	1	2	1	51.3	6.55	3.96	1	x	x					2-Hacid_dh_C	PF02826.20		Oxidation-reduction
NEMBOFW57_006149	9	2	2	2	29.9	5.91	3.94	2		x					adh_short	PF00106.26		Oxidation-reduction
NEMBOFW57_004842	16	3	3	3	29.4	6.44	3.73	3		x					GST_C_3	PF14497.7		Oxidation-reduction
NEMBOFW57_008224	14	3	3	3	24.3	6.2	3.65	3		x		x			adh_short	PF00106.26		Oxidation-reduction
NEMBOFW57_004929	5	1	1	1	34.5	6.18	3.45	1		x		x			ADH_zinc_N	PF00107.27		Oxidation-reduction
NEMBOFW57_006232	5	1	1	1	37.8	7.4	3.31	1						x	ADH_N	PF08240.13		Oxidation-reduction
NEMBOFW57_006135	9	4	4	4	57.5	7.53	3.14	4		x					Acyl-CoA_dh_1	PF00441.25		Oxidation-reduction
NEMBOFW57_001431	2	1	1	1	81.3	5.11	2.84	1				x			Thioredox_DsbH	PF03190.16		Oxidation-reduction
NEMBOFW57_008654	2	1	1	1	56.9	8.4	2.83	1		x					Aldedh	PF00171.23		Oxidation-reduction
NEMBOFW57_005222	3	1	1	1	49.1	5.66	2.83	1				x			Sacchrp_dh_C	PF16653.6		Oxidation-reduction

NEMBOFW57_008591	3	1	1	1	63.0	7.68	2.81	1						x	p450	PF00067.23		Oxidation-reduction
NEMBOFW57_010036	4	1	1	1	31.4	8.73	2.5	1						x	adh_short_C2	PF13561.7		Oxidation-reduction
NEMBOFW57_007437	9	2	2	2	33.2	5.72	2.38	2	x	x					NAD_binding_2	PF03446.16	putative oxidoreductase YfjR [Madurella mycetomatis]	Oxidation-reduction
NEMBOFW57_004165	3	1	1	1	28.4	6.23	2.32	1	x	x					adh_short_C2	PF13561.7		Oxidation-reduction
NEMBOFW57_005917	2	1	1	1	51.8	6.71	2.2	1				x			ECH_2	PF16113.6		Oxidation-reduction
NEMBOFW57_010349	9	2	2	2	40.4	8.9	2.13	2		x					Acyl-CoA_dh_1	PF00441.25		Oxidation-reduction
NEMBOFW57_002625	10	3	3	3	51.9	6.19	2.01	3		x					Aldedh	PF00171.23		Oxidation-reduction
NEMBOFW57_003058	1	1	1	1	72.9	5.72	1.93	1		x					FAD_binding_3	PF01494.20	Putative monooxygenase [Podospora comata]	Oxidation-reduction
NEMBOFW57_000420	4	1	1	1	32.0	9.01	1.9	1		x					adh_short	PF00106.26		Oxidation-reduction
NEMBOFW57_001531	6	2	2	2	34.2	6.54	1.87	2		x					Aldo_ket_red	PF00248.22		Oxidation-reduction
NEMBOFW57_001574	7	1	1	1	14.7	9.54	1.82	1	x	x					COX6A	PF02046.16		Oxidation-reduction
NEMBOFW57_009846	8	2	2	2	26.1	7.62	1.8	2		x					adh_short	PF00106.26		Oxidation-reduction
NEMBOFW57_001451	1	1	1	1	106.6	5.57	1.8	1		x					FAD_binding_4	PF01565.24	FAD-dependent monooxygenase yanF [Lachnellula arida]	Oxidation-reduction
NEMBOFW57_003056	2	1	1	1	42.0	9.44	1.79	1		x					adh_short	PF00106.26		Oxidation-reduction

NEMBOFW57_004469	5	3	3	3	71.3	7.9	1.78	3		x					FAD_binding_3	PF01494.20	Phenol 2-monooxygenase [Madurella mycetomatis]	Oxidation-reduction
NEMBOFW57_001627	4	1	1	1	27.6	6.96	1.76	1		x					adh_short	PF00106.26		Oxidation-reduction
NEMBOFW57_005340	6	1	1	1	18.6	5.74	1.68	1		x					GSHPx	PF00255.20		Oxidation-reduction
NEMBOFW57_002493	2	1	3	1	40.7	5.68	1.67	1		x					AlaDh_PNT_N	PF05222.16		Oxidation-reduction
NEMBOFW57_005228	3	1	1	1	39.4	7.4	1.64	1		x					Thioredoxin	PF00085.21		Oxidation-reduction
NEMBOFW57_010051	5	2	2	2	51.3	5.63	0.0	2		x					Aldedh	PF00171.23		Oxidation-reduction
NEMBOFW57_009889	10	2	2	2	37.3	6.54	0.0	2		x					ADH_N	PF08240.13		Oxidation-reduction
NEMBOFW57_003456	6	2	2	2	53.4	6.35	0.0	2		x					Aldedh	PF00171.23		Oxidation-reduction
NEMBOFW57_003571	2	1	1	1	55.4	8.12	0.0	1		x					Peroxidase_2	PF01328.18		Oxidation-reduction
NEMBOFW57_005343	3	1	1	1	46.0	6.54	0.0	1		x					GFO_IDH_MocA	PF01408.23		Oxidation-reduction
NEMBOFW57_007835	2	1	1	1	67.8	7.11	0.0	1		x					FAD_binding_4	PF01565.24	related to isoamyl alcohol oxidase [Phialocephala subalpina]	Oxidation-reduction
NEMBOFW57_004990	17	1	1	1	13.0	5.6	0.0	1		x					Cyt-b5	PF00173.29		Oxidation-reduction
NEMBOFW57_001733	3	1	1	1	45.4	8.05	0.0	1		x					Acyl-CoA_dh_1	PF00441.25		Oxidation-reduction
NEMBOFW57_002936	3	1	1	1	38.0	5.5	0.0	1		x					Iso_dh	PF00180.21		Oxidation-reduction

NEMBOFW57_005896	3	1	1	1	31.2	5.88	0.0	1		x		x			adh_short_C2	PF13561.7		Oxidation-reduction
NEMBOFW57_004761	40	11	35	11	45.9	9.11	130.11	11	x	x	x	x	x	x	Aminotran_1_2	PF00155.22		Protein metabolism
NEMBOFW57_007373	23	14	26	14	93.2	6.81	89.8	14	x	x	x	x		x	GTP_EFTU	PF00009.28		Protein metabolism
NEMBOFW57_002142	44	10	27	10	43.9	6.11	89.14	10	x	x	x	x	x	x	Aminotran_5	PF00266.20		Protein metabolism
NEMBOFW57_006799	32	7	23	7	32.5	6.65	81.9	7		x	x	x	x	x	SOR_SNZ	PF01680.18		Protein metabolism
NEMBOFW57_002751	21	20	25	20	131.2	6.38	81.31	20	x	x	x	x			CPSase_L_D2	PF02786.18		Protein metabolism
NEMBOFW57_000856	24	7	24	7	56.7	8.28	79.7	7		x	x	x	x	x	Aminotran_3	PF00202.22		Protein metabolism
NEMBOFW57_004362	37	10	22	10	48.4	6.18	74.51	10		x	x	x	x	x	Cys_Met_Meta_PP	PF01053.21		Protein metabolism
NEMBOFW57_001610	32	12	33	12	49.9	9.13	67.97	12	x	x	x	x	x	x	GTP_EFTU	PF00009.28		Protein metabolism
NEMBOFW57_009407	20	6	17	6	60.5	6.0	65.78	6			x	x	x	x	TPP_enzyme_N	PF02776.19		Protein metabolism
NEMBOFW57_001883	41	8	17	8	38.9	6.79	64.93	8	x	x	x	x	x	x	Semialdhyde_dhC	PF02774.19		Protein metabolism
NEMBOFW57_002515	23	15	23	15	92.9	5.85	64.1	15			x	x		x	Histidinol_dh	PF00815.21		Protein metabolism
NEMBOFW57_008836	26	6	17	6	50.5	6.07	57.38	6	x	x	x	x	x	x	HgmA	PF04209.14		Protein metabolism
NEMBOFW57_008699	29	6	16	6	40.3	6.02	57.31	6		x	x	x	x	x	Pro_isomerase	PF00160.22		Protein metabolism

NEMBOFW57_000790	58	15	34	15	28.5	8.95	54.02	15	x	x					Ribosomal_S3_C	PF00189.21		Protein metabolism
NEMBOFW57_005936	45	13	20	13	38.8	10.62	52.58	13		x					Ribosomal_L4	PF00573.23		Protein metabolism
NEMBOFW57_004447	32	8	14	8	46.1	5.67	48.66	8		x	x	x	x	x	Arginosuc_synth	PF00764.20		Protein metabolism
NEMBOFW57_004340	33	7	13	7	38.5	6.92	44.83	7	x		x	x	x	x	OTCace	PF00185.25		Protein metabolism
NEMBOFW57_001977	48	16	21	16	41.0	8.0	41.15	16		x					Tyrosinase	PF00264.21		Protein metabolism
NEMBOFW57_005475	17	3	10	3	22.6	7.65	39.3	3		x	x	x	x	x	Pro_isomerase	PF00160.22		Protein metabolism
NEMBOFW57_002414	26	7	11	7	36.3	5.85	38.46	7			x	x		x	HisG	PF01634.19		Protein metabolism
NEMBOFW57_002891	45	7	18	7	20.4	6.79	34.6	7	x	x					Pro_isomerase	PF00160.22		Protein metabolism
NEMBOFW57_010437	31	6	18	6	28.7	10.4	32.94	6	x	x					Ribosomal_S5	PF00333.21		Protein metabolism
NEMBOFW57_003385	30	4	10	4	26.4	6.38	31.89	4	x		x	x	x	x	FAA_hydrolase	PF01557.19		Protein metabolism
NEMBOFW57_009823	36	7	19	7	24.1	11.46	31.58	7	x	x		x			Ribosomal_L15e	PF00827.18		Protein metabolism
NEMBOFW57_003108	42	3	7	3	12.8	5.3	29.24	3		x	x	x	x	x	SBDS	PF01172.19		Protein metabolism
NEMBOFW57_001692	32	7	13	7	29.5	10.15	27.47	7	x	x					Ribosomal_S4e	PF00900.21		Protein metabolism
NEMBOFW57_010101	34	7	16	7	31.8	4.84	27.22	7	x	x		x			Ribosomal_S2	PF00318.21		Protein metabolism

NEMBOFW57_003018	45	7	15	7	15.9	10.32	26.12	7		x					Ribosomal_S9	PF00380.20		Protein metabolism
NEMBOFW57_009920	18	4	7	4	47.6	7.61	26.01	4	x	x	x	x	x	x	Aminotran_5	PF00266.20		Protein metabolism
NEMBOFW57_005382	38	8	17	8	25.2	10.67	25.56	8	x	x					Ribosomal_S3Ae	PF01015.19		Protein metabolism
NEMBOFW57_004587	24	5	9	5	22.5	8.84	24.75	5	x	x					Ribosomal_S7	PF00177.22		Protein metabolism
NEMBOFW57_000934	24	5	15	5	31.4	11.09	23.37	5	x	x					Ribosomal_L2_C	PF03947.19		Protein metabolism
NEMBOFW57_001719	46	3	6	3	15.5	5.77	23.33	3			x	x		x	MmgE_PrpD	PF03972.15		Protein metabolism
NEMBOFW57_008913	24	6	14	6	29.4	10.27	23.22	6		x					Ribosomal_L7Ae	PF01248.27		Protein metabolism
NEMBOFW57_000203	33	6	13	6	20.3	9.8	23.13	6	x	x					Ribosomal_S11	PF00411.20		Protein metabolism
NEMBOFW57_002291	47	6	17	6	21.8	9.48	21.43	6	x	x					Ribosomal_L6	PF00347.24		Protein metabolism
NEMBOFW57_001562	38	9	12	9	16.0	9.79	20.37	9	x	x		x			Ribosomal_S4	PF00163.20		Protein metabolism
NEMBOFW57_009768	14	5	8	5	44.1	10.14	20.04	5	x	x		x			Ribosomal_L3	PF00297.23		Protein metabolism
NEMBOFW57_000202	26	6	13	6	30.5	10.11	19.59	6	x	x					Ribosomal_L30_N	PF08079.13		Protein metabolism
NEMBOFW57_001335	34	7	10	7	23.0	10.29	18.29	7	x	x					Ribosomal_S7e	PF01251.19		Protein metabolism
NEMBOFW57_003386	38	4	6	4	20.5	5.58	18.19	4				x		x	3-HAO	PF06052.13		Protein metabolism

NEMBOFW57_005161	36	4	12	4	17.7	9.28	17.8	4		x					Ribosomal_L11_N	PF03946.15		Protein metabolism
NEMBOFW57_002881	8	5	6	5	130.4	5.95	17.8	5				x			AMP-binding	PF00501.29	L-aminoadipate-semialdehyde dehydrogenase large subunit [Madurella mycetomatis]	Protein metabolism
NEMBOFW57_001804	28	3	6	3	22.3	7.72	17.5	3		x	x	x	x	x	eIF-5a	PF01287.21		Protein metabolism
NEMBOFW57_004950	17	5	8	5	44.9	8.62	17.24	5	x	x		x		x	IlvN	PF07991.13		Protein metabolism
NEMBOFW57_000778	27	5	11	5	17.7	10.55	17.01	5	x	x					Ribosomal_S13	PF00416.23		Protein metabolism
NEMBOFW57_009144	19	9	10	8	59.6	9.14	16.84	9		x					SHMT	PF00464.20		Protein metabolism
NEMBOFW57_002599	28	6	11	6	30.9	5.99	16.78	6	x	x	x	x	x	x	RL10P_insert	PF17777.2		Protein metabolism
NEMBOFW57_009798	21	4	10	4	27.2	10.52	15.6	4	x	x					Ribosomal_S6e	PF01092.20		Protein metabolism
NEMBOFW57_002108	33	5	7	5	16.8	10.32	15.29	5	x	x					Ribosomal_S13_N	PF08069.13		Protein metabolism
NEMBOFW57_005849	48	7	10	7	20.8	10.43	15.0	7		x					Ribosomal_L22	PF00237.20		Protein metabolism
NEMBOFW57_002748	12	2	6	2	19.9	10.13	14.12	2	x	x					Ribosomal_L5	PF00281.20		Protein metabolism
NEMBOFW57_002478	24	3	8	3	18.5	10.83	13.91	3	x	x					Ribosomal_S17_N	PF16205.6		Protein metabolism
NEMBOFW57_003842	18	7	9	6	53.5	7.37	13.52	7		x	x	x			SHMT	PF00464.20		Protein metabolism

NEMBOFW57_000878	34	5	10	5	23.9	9.92	13.33	5	x	x					Ribosomal_L1	PF00687.22		Protein metabolism
NEMBOFW57_003361	52	8	11	8	20.5	10.71	13.21	8	x	x					Ribosomal_L18A	PF01775.18		Protein metabolism
NEMBOFW57_010423	22	3	4	3	25.4	4.48	12.54	3	x	x	x	x		x	EF1_GNE	PF00736.20		Protein metabolism
NEMBOFW57_009877	50	7	10	7	15.8	10.45	12.53	7	x	x					Ribosomal_L27e	PF01777.19		Protein metabolism
NEMBOFW57_005858	27	8	9	8	44.7	7.44	12.21	8		x		x		x	EF1G	PF00647.20		Protein metabolism
NEMBOFW57_009800	35	5	9	5	22.3	10.68	12.02	5		x					Ribosomal_S8e	PF01201.23		Protein metabolism
NEMBOFW57_001932	3	3	4	3	183.5	7.68	11.89	3				x			CPSase_L_D2	PF02786.18		Protein metabolism
NEMBOFW57_000255	11	3	4	3	48.4	6.38	11.31	3			x	x			Aminotran_3	PF00202.22		Protein metabolism
NEMBOFW57_002279	37	6	7	6	22.2	10.21	11.08	6		x					Ribosomal_L6e	PF01159.20		Protein metabolism
NEMBOFW57_001844	23	4	9	4	18.0	11.39	11.0	4	x	x					Ribosomal_L24e	PF01246.21		Protein metabolism
NEMBOFW57_006121	27	5	5	5	34.7	7.59	10.96	5		x				x	Ribosomal_L5e	PF17144.5		Protein metabolism
NEMBOFW57_001561	16	3	5	3	18.2	10.45	10.88	3	x	x					Ribosomal_L21e	PF01157.19		Protein metabolism
NEMBOFW57_008773	34	7	8	7	24.0	11.11	10.36	7		x					Ribosomal_L13e	PF01294.19		Protein metabolism
NEMBOFW57_007770	16	4	4	4	45.9	6.87	10.24	4	x	x	x	x			Aminotran_1_2	PF00155.22		Protein metabolism

NEMBOFW57_005179	18	2	5	2	12.0	11.62	9.34	2	x	x					Ribosomal_L36e	PF01158.19		Protein metabolism
NEMBOFW57_003321	27	3	4	3	13.9	10.35	9.3	3	x	x					Ribosomal_L31e	PF01198.20		Protein metabolism
NEMBOFW57_000509	24	3	6	3	17.0	10.24	9.17	3	x	x					Ribosomal_L23eN	PF03939.14		Protein metabolism
NEMBOFW57_000221	17	3	4	3	13.0	10.64	9.04	3	x	x					Ribosomal_L34e	PF01199.19		Protein metabolism
NEMBOFW57_005139	14	2	3	2	24.4	6.74	9.0	2		x	x	x			IGPD	PF00475.19		Protein metabolism
NEMBOFW57_001858	37	5	5	5	15.9	10.67	8.68	5		x					Ribosomal_L14e	PF01929.18		Protein metabolism
NEMBOFW57_005066	23	4	4	4	15.1	9.47	8.49	4		x					Ribosomal_L22e	PF01776.18		Protein metabolism
NEMBOFW57_007463	2	2	2	2	244.4	6.46	8.45	2				x			CPSase_L_D2	PF02786.18		Protein metabolism
NEMBOFW57_006643	29	3	4	3	16.7	9.83	7.15	3		x					Ribosomal_S17e	PF00833.19		Protein metabolism
NEMBOFW57_005894	26	3	3	3	14.8	10.43	7.14	3		x					Ribosomal_L27A	PF00828.20		Protein metabolism
NEMBOFW57_007908	16	5	5	5	49.9	5.72	7.02	5		x					Aminotran_5	PF00266.20		Protein metabolism
NEMBOFW57_000199	39	5	5	5	17.0	7.64	6.98	5		x					Ribosomal_S19e	PF01090.20		Protein metabolism
NEMBOFW57_007554	16	1	3	1	17.3	5.4	6.71	1	x	x		x			eIF-5a	PF01287.21		Protein metabolism
NEMBOFW57_005469	2	1	3	1	48.9	6.32	6.64	1		x	x	x			ELFV_dehydrog	PF00208.22		Protein metabolism

NEMBOFW57_007291	16	3	3	3	30.9	5.26	6.14	3		x					FAA_hydrolase	PF01557.19		Protein metabolism
NEMBOFW57_009415	14	2	3	2	16.4	9.95	6.13	2		x					Ribosomal_S10	PF00338.23		Protein metabolism
NEMBOFW57_004187	30	3	6	3	13.5	10.78	6.04	3		x					Ribosomal_S26e	PF01283.20		Protein metabolism
NEMBOFW57_002254	2	2	2	2	127.5	6.33	5.85	2	x	x		x			CPSase_L_D2	PF02786.18		Protein metabolism
NEMBOFW57_002280	22	4	4	4	15.0	11.41	5.74	4		x					Ribosomal_L29	PF00831.24		Protein metabolism
NEMBOFW57_005227	4	2	3	2	48.9	6.96	5.42	2	x	x					GTP_EFTU	PF00009.28		Protein metabolism
NEMBOFW57_010223	28	4	5	4	15.5	10.67	5.24	4		x					Ribosomal_L26	PF16906.6		Protein metabolism
NEMBOFW57_003164	33	3	3	3	12.2	10.86	4.97	3		x					Ribosomal_L35Ae	PF01247.19		Protein metabolism
NEMBOFW57_000382	18	2	2	2	14.8	10.05	4.84	2		x		x			Ribosomal_S8	PF00410.20		Protein metabolism
NEMBOFW57_000713	16	2	2	2	14.9	11.43	4.82	2		x					Ribosomal_L32e	PF01655.19		Protein metabolism
NEMBOFW57_006026	2	1	2	1	63.3	6.44	4.8	1				x			GATase_6	PF13522.7		Protein metabolism
NEMBOFW57_010953	7	2	3	2	35.4	6.54	4.69	2	x	x					DHDPS	PF00701.23		Protein metabolism
NEMBOFW57_005682	24	6	6	6	22.9	10.39	4.52	6		x					Ribosomal_L13	PF00572.19		Protein metabolism
NEMBOFW57_010019	5	3	3	3	43.0	7.46	3.95	3		x					Tyrosinase	PF00264.21		Protein metabolism

NEMBOFW57_008802	4	1	1	1	45.7	5.58	3.91	1		x		x		x	FAA_hydrolase	PF01557.19		Protein metabolism
NEMBOFW57_002837	5	2	2	2	42.7	5.83	3.84	2	x	x					Aminotran_4	PF01063.20		Protein metabolism
NEMBOFW57_005403	5	2	3	2	46.0	6.71	3.8	2		x					Aminotran_5	PF00266.20		Protein metabolism
NEMBOFW57_000383	20	3	5	3	16.3	5.02	3.47	3	x	x					Ribosomal_L7Ae	PF01248.27		Protein metabolism
NEMBOFW57_007623	6	4	4	4	114.9	5.17	3.46	4		x					ThiF	PF00899.22		Protein metabolism
NEMBOFW57_008411	4	1	1	1	53.3	5.77	2.96	1		x	x				Aminotran_1_2	PF00155.22		Protein metabolism
NEMBOFW57_004401	1	1	1	1	233.4	6.29	2.77	1				x			GATase_2	PF00310.22		Protein metabolism
NEMBOFW57_006380	29	4	4	4	15.5	10.84	2.54	4		x					Ribosomal_S24e	PF01282.20		Protein metabolism
NEMBOFW57_000293	13	1	1	1	14.9	11.52	2.49	1		x					Ribosomal_L28e	PF01778.18		Protein metabolism
NEMBOFW57_001874	18	1	1	1	9.6	9.22	2.24	1		x					Ribosomal_S27e	PF01667.18		Protein metabolism
NEMBOFW57_008716	24	1	1	1	7.7	10.76	2.17	1		x					Ribosomal_S28e	PF01200.19		Protein metabolism
NEMBOFW57_004586	10	1	2	1	10.9	5.5	2.14	1		x					Ribosomal_S25	PF03297.16		Protein metabolism
NEMBOFW57_003730	12	2	2	2	14.6	10.33	2.06	2		x					Ribosomal_L14	PF00238.20		Protein metabolism
NEMBOFW57_009427	2	1	1	1	49.3	5.66	1.97	1		x					eIF3_N	PF09440.11		Protein metabolism

NEMBOFW57_005256	8	1	3	1	11.9	10.1	1.9	1		x					Ribosomal_L38e	PF01781.19		Protein metabolism
NEMBOFW57_006177	12	2	2	2	11.7	9.67	1.84	2		x					Ribosomal_L7Ae	PF01248.27		Protein metabolism
NEMBOFW57_000818	11	1	1	1	7.9	10.42	1.72	1		x					Ribosomal_L37ae	PF01780.20		Protein metabolism
NEMBOFW57_004163	7	2	2	2	39.4	6.37	1.6	2		x					Tyrosinase	PF00264.21		Protein metabolism
NEMBOFW57_003037	2	1	2	1	55.5	8.13	0.0	1	x						eIF2_C	PF09173.12		Protein metabolism
NEMBOFW57_007710	3	1	1	1	42.4	7.72	0.0	1		x					Tyrosinase	PF00264.21		Protein metabolism
NEMBOFW57_000850	3	1	1	1	26.5	4.72	0.0	1		x					eIF-6	PF01912.19		Protein metabolism
NEMBOFW57_010357	1	1	1	1	85.2	5.1	0.0	1		x					eIF2A	PF08662.12		Protein metabolism
NEMBOFW57_002583	45	29	78	29	97.6	5.44	263.72	29	x	x	x	x	x	x	ERAP1_C	PF11838.9		Proteolysis
NEMBOFW57_009314	29	18	43	18	99.6	5.57	135.77	18		x	x	x	x	x	Peptidase_M1	PF01433.21		Proteolysis
NEMBOFW57_004704	40	13	33	13	55.0	6.7	130.41	13	x	x	x	x	x	x	Peptidase_M28	PF04389.18		Proteolysis
NEMBOFW57_002046	16	9	34	9	107.8	6.79	118.18	9	x	x	x	x	x	x	Peptidase_S28	PF05577.13		Proteolysis
NEMBOFW57_001262	17	8	20	8	75.3	6.52	63.36	8	x	x	x	x	x	x	Peptidase_M24	PF00557.25		Proteolysis
NEMBOFW57_006866	20	10	16	10	92.6	6.42	50.24	10		x	x	x	x	x	Peptidase_M3	PF01432.21		Proteolysis
NEMBOFW57_000332	22	7	15	7	52.1	6.24	49.24	7	x	x		x		x	Peptidase_M18	PF02127.16		Proteolysis
NEMBOFW57_001132	29	16	21	16	88.8	6.86	46.03	16	x	x	x	x	x	x	DPPIV_N	PF00930.22		Proteolysis
NEMBOFW57_004069	27	4	12	4	23.0	7.08	42.55	4			x	x	x	x	HRXXH	PF13933.7		Proteolysis
NEMBOFW57_001130	17	3	12	3	27.0	8.57	38.89	3	x	x	x	x	x	x	DJ-1_PfpI	PF01965.25		Proteolysis
NEMBOFW57_001330	18	10	14	10	80.5	6.76	31.89	10	x	x	x	x	x	x	Peptidase_S8	PF00082.23		Proteolysis
NEMBOFW57_005294	10	3	8	3	32.6	6.57	28.52	3	x	x	x	x	x	x	Peptidase_A4	PF01828.18		Proteolysis

NEMBOFW57_003829	30	8	12	8	42.9	5.17	24.37	8	x	x		x		x	Asp	PF00026.24		Proteolysis
NEMBOFW57_000018	11	2	6	2	44.2	5.34	21.39	2		x	x	x	x	x	Peptidase_M28	PF04389.18		Proteolysis
NEMBOFW57_009119	21	7	10	7	40.1	6.64	21.25	7	x	x					Peptidase_S8	PF00082.23		Proteolysis
NEMBOFW57_010961	14	4	7	4	53.1	6.0	21.2	4	x		x	x	x		Peptidase_M28	PF04389.18		Proteolysis
NEMBOFW57_006901	33	6	7	6	24.0	5.27	19.73	6	x	x	x	x		x	DJ-1_PfpI	PF01965.25		Proteolysis
NEMBOFW57_009044	21	8	9	8	54.6	6.3	17.8	8	x	x					Peptidase_S28	PF05577.13		Proteolysis
NEMBOFW57_005172	8	5	5	5	112.1	6.19	16.85	5				x			M16C_assoc	PF08367.12		Proteolysis
NEMBOFW57_006358	14	3	4	3	39.9	8.1	15.96	3		x		x	x	x	Peptidase_S8	PF00082.23		Proteolysis
NEMBOFW57_010772	7	2	4	2	86.6	6.25	14.38	2			x	x	x	x	Peptidase_S8	PF00082.23		Proteolysis
NEMBOFW57_005284	11	4	5	4	55.9	7.27	14.32	4	x	x	x	x	x		Peptidase_M18	PF02127.16		Proteolysis
NEMBOFW57_002351	21	5	5	5	29.2	6.89	11.65	5	x	x		x		x	Proteasome	PF00227.27		Proteolysis
NEMBOFW57_007537	25	5	7	5	26.9	4.86	11.52	5	x	x		x			Proteasome	PF00227.27		Proteolysis
NEMBOFW57_005408	28	6	8	6	28.9	5.4	11.42	6	x	x	x	x		x	Proteasome	PF00227.27		Proteolysis
NEMBOFW57_008113	25	6	8	6	29.2	7.77	11.08	6	x	x					Proteasome	PF00227.27		Proteolysis
NEMBOFW57_001765	8	4	6	4	57.4	6.61	10.05	4	x	x					Peptidase_S8	PF00082.23		Proteolysis
NEMBOFW57_006357	10	3	3	3	52.3	5.15	9.12	3		x		x			Peptidase_M20	PF01546.29		Proteolysis
NEMBOFW57_002425	10	2	3	2	31.4	6.43	8.96	2	x	x		x			Proteasome	PF00227.27		Proteolysis
NEMBOFW57_000405	26	5	6	5	29.5	7.4	8.16	5	x	x		x		x	Proteasome	PF00227.27		Proteolysis
NEMBOFW57_002765	17	4	4	4	30.7	5.12	7.77	4	x	x		x		x	Proteasome	PF00227.27		Proteolysis
NEMBOFW57_002083	17	2	2	2	30.3	5.97	7.11	2	x	x		x		x	Proteasome	PF00227.27		Proteolysis
NEMBOFW57_003522	10	4	4	4	46.1	7.8	7.0	4	x		x				Abhydrolase_1	PF00561.21	proteinase [Aspergillus flavus]	Proteolysis
NEMBOFW57_006188	16	5	5	5	27.9	7.36	6.72	5	x	x					Proteasome	PF00227.27		Proteolysis
NEMBOFW57_005311	16	3	3	3	22.5	7.53	6.13	3	x	x					Proteasome	PF00227.27		Proteolysis
NEMBOFW57_005333	16	6	6	6	43.0	5.55	5.93	6	x	x					Peptidase_M19	PF01244.22		Proteolysis
NEMBOFW57_003937	6	2	2	2	74.5	9.26	5.36	2			x	x			eIF2A	PF08662.12		Proteolysis
NEMBOFW57_007393	1	1	2	1	103.7	7.09	4.61	1				x			Lon_C	PF05362.14		Proteolysis
NEMBOFW57_010378	12	2	2	2	22.6	7.01	4.5	2	x	x					Proteasome	PF00227.27		Proteolysis

NEMBOFW57_006015	2	1	1	1	69.1	5.63	4.48	1				x			Peptidase_M1	PF01433.21		Proteolysis
NEMBOFW57_010937	7	3	3	3	66.5	5.87	4.28	3		x					PepX_C	PF08530.11		Proteolysis
NEMBOFW57_004435	9	2	4	2	24.5	9.25	4.01	2	x	x				x	Proteasome	PF00227.27		Proteolysis
NEMBOFW57_010208	2	1	3	1	47.1	8.94	3.98	1	x	x					Peptidase_M16	PF00675.21		Proteolysis
NEMBOFW57_004414	8	3	3	3	56.6	6.74	3.92	3		x	x	x			Asp	PF00026.24		Proteolysis
NEMBOFW57_003977	6	1	1	1	44.2	7.28	3.88	1			x				Peptidase_M24	PF00557.25		Proteolysis
NEMBOFW57_003380	3	1	1	1	57.1	6.23	3.78	1				x			Peptidase_S9	PF00326.22		Proteolysis
NEMBOFW57_003141	13	2	2	2	22.5	5.39	3.61	2	x	x		x			Proteasome	PF00227.27		Proteolysis
NEMBOFW57_000780	11	4	4	4	62.0	5.95	3.6	4	x	x					Peptidase_S10	PF00450.23		Proteolysis
NEMBOFW57_002274	2	1	1	1	77.9	5.5	2.72	1				x			Peptidase_M49	PF03571.16		Proteolysis
NEMBOFW57_000439	3	2	2	2	120.5	9.47	2.4	2		x					PCI	PF01399.28		Proteolysis
NEMBOFW57_003848	3	2	2	2	70.1	4.94	2.39	2	x	x					Peptidase_S10	PF00450.23		Proteolysis
NEMBOFW57_007692	3	1	1	1	41.4	6.14	2.35	1					x		Peptidase_M14	PF00246.25		Proteolysis
NEMBOFW57_006198	8	1	1	1	19.6	8.92	2.17	1	x	x					Peptidase_M57	PF12388.9		Proteolysis
NEMBOFW57_002952	3	1	1	1	52.4	5.66	1.86	1		x					Peptidase_M16	PF00675.21		Proteolysis
NEMBOFW57_008659	2	1	1	1	60.9	5.9	0.0	1	x						Peptidase_S15	PF02129.19		Proteolysis
NEMBOFW57_002864	6	1	1	1	30.4	5.78	0.0	1	x	x					Peptidase_A4	PF01828.18		Proteolysis
NEMBOFW57_000899	2	1	2	1	45.5	6.68	0.0	1		x					Peptidase_M24	PF00557.25		Proteolysis
NEMBOFW57_004065	1	1	1	1	125.4	5.12	0.0	1		x					RPN2_C	PF18004.2		Proteolysis
NEMBOFW57_007398	1	1	1	1	100.2	5.02	0.0	1		x					RPN1_RPN2_N	PF17781.2		Proteolysis
NEMBOFW57_008732	10	1	1	1	25.8	8.95	0.0	1	x	x					Peptidase_A4	PF01828.18		Proteolysis
NEMBOFW57_003006	5	1	1	1	29.1	7.2	0.0	1		x					Proteasome	PF00227.27		Proteolysis
NEMBOFW57_004747	2	1	1	1	79.9	7.02	0.0	1		x					Asp	PF00026.24		Proteolysis
NEMBOFW57_006951	2	1	1	1	45.3	6.1	0.0	1		x					Peptidase_M28	PF04389.18		Proteolysis
NEMBOFW57_010510	30	13	21	13	54.0	8.16	81.11	13	x	x	x	x	x	x	Pyr_redox_2	PF07992.15		RNA metabolism
NEMBOFW57_000553	22	8	20	8	52.9	6.35	70.0	8			x	x	x	x	ADSL_C	PF10397.10		RNA metabolism
NEMBOFW57_003032	44	9	19	9	27.1	5.03	60.79	9	x	x	x	x	x	x	14-3-3	PF00244.21		RNA metabolism

NEMBOFW57_002745	23	12	28	12	59.6	9.28	60.69	12	x	x	x	x	x	x	ATP-synt_ab	PF00006.26		RNA metabolism
NEMBOFW57_007542	40	6	15	6	25.0	5.8	46.87	6		x	x	x	x	x	Pribosyltran	PF00156.28		RNA metabolism
NEMBOFW57_009710	19	6	18	6	49.3	5.24	41.31	6	x	x	x	x	x	x	ATP-synt_ab	PF00006.26		RNA metabolism
NEMBOFW57_003083	25	9	11	9	58.2	6.9	38.6	9	x	x		x			UDPGP	PF01704.19		RNA metabolism
NEMBOFW57_005292	43	6	14	6	16.7	7.39	35.79	6	x	x	x	x	x	x	NDK	PF00334.20		RNA metabolism
NEMBOFW57_006545	20	8	16	8	38.7	5.33	33.94	8	x	x	x	x	x	x	Pyr_redox_2	PF07992.15		RNA metabolism
NEMBOFW57_000798	15	4	9	4	46.0	6.35	25.1	4			x	x			Adenylsucc_synt	PF00709.22		RNA metabolism
NEMBOFW57_010061	26	5	6	4	47.3	6.92	24.49	5	x	x	x	x	x	x	Pyr_redox_2	PF07992.15		RNA metabolism
NEMBOFW57_002405	44	3	6	3	12.0	7.01	24.25	3	x	x	x	x	x	x	Ribonuc_L-PSP	PF01042.22		RNA metabolism
NEMBOFW57_003136	37	8	10	8	29.3	5.48	22.3	8	x	x		x			14-3-3	PF00244.21		RNA metabolism
NEMBOFW57_009188	28	11	11	11	57.3	7.47	21.49	11		x					Pyr_redox_2	PF07992.15		RNA metabolism
NEMBOFW57_002589	12	4	11	4	44.8	5.19	21.43	4	x	x	x		x		DEAD	PF00270.30		RNA metabolism
NEMBOFW57_003105	26	5	13	5	24.1	6.92	20.76	5	x	x					Ras	PF00071.23		RNA metabolism
NEMBOFW57_006227	26	2	5	2	14.7	9.32	19.34	2			x	x	x	x	Pyr_redox_2	PF07992.15		RNA metabolism
NEMBOFW57_000215	14	3	6	3	33.6	6.58	19.11	3			x	x			Uricase	PF01014.19		RNA metabolism
NEMBOFW57_007621	2	1	5	1	81.5	4.53	18.13	1			x	x		x	Utp14	PF04615.14		RNA metabolism
NEMBOFW57_000017	12	4	6	4	61.1	5.71	16.18	4	x	x	x	x	x	x	5_nucleotid_C	PF02872.19		RNA metabolism
NEMBOFW57_003963	5	2	8	2	39.8	6.7	15.36	2	x	x					NMT1	PF09084.12		RNA metabolism
NEMBOFW57_007389	12	2	4	2	29.5	5.39	14.38	2		x	x	x		x	NUDIX	PF00293.29		RNA metabolism
NEMBOFW57_003265	13	4	4	4	53.1	5.25	12.28	4				x			UDPGP	PF01704.19		RNA metabolism
NEMBOFW57_005544	8	3	3	3	72.8	6.77	11.12	3			x	x			tRNA-synt_1c	PF00749.22		RNA metabolism
NEMBOFW57_007251	15	5	6	5	37.7	6.05	10.96	5	x	x					RRM_1	PF00076.23		RNA metabolism
NEMBOFW57_005312	4	3	5	3	72.1	8.6	10.35	3	x	x					DEAD	PF00270.30		RNA metabolism
NEMBOFW57_010507	19	6	6	6	43.4	6.73	9.66	6		x					RRM_1	PF00076.23		RNA metabolism
NEMBOFW57_004416	18	3	5	3	21.6	6.87	9.23	3		x					Ras	PF00071.23		RNA metabolism
NEMBOFW57_000570	13	2	4	2	21.7	6.89	9.05	2	x	x		x			Arf	PF00025.22		RNA metabolism
NEMBOFW57_006880	10	3	3	3	49.8	5.99	8.27	3				x			GMP_synt_C	PF00958.23		RNA metabolism

NEMBOFW57_008893	9	6	6	6	82.6	6.07	8.02	6		x					RRM_1	PF00076.23		RNA metabolism
NEMBOFW57_000282	6	3	3	3	82.9	5.4	5.48	3				x			GARS_A	PF01071.20		RNA metabolism
NEMBOFW57_003462	2	1	2	1	64.1	6.38	5.39	1				x			tRNA-synt_2	PF00152.21		RNA metabolism
NEMBOFW57_009810	21	4	4	4	22.4	5.44	5.23	4		x	x			x	Ras	PF00071.23		RNA metabolism
NEMBOFW57_007203	3	2	2	2	94.5	7.27	5.03	2			x				tRNA_lig_CPD	PF08302.12		RNA metabolism
NEMBOFW57_002360	17	2	2	2	11.3	9.42	4.97	2		x					HMG_box	PF00505.20		RNA metabolism
NEMBOFW57_003188	5	2	2	2	64.8	6.77	4.85	2				x			AICARFT_IMPCHas	PF01808.19		RNA metabolism
NEMBOFW57_008846	8	2	2	2	29.4	6.62	3.9	2		x					RRM_1	PF00076.23		RNA metabolism
NEMBOFW57_001799	8	1	2	1	32.9	6.35	3.82	1		x		x			S1-P1_nuclease	PF02265.17		RNA metabolism
NEMBOFW57_008123	7	3	3	3	48.8	6.76	3.58	3		x					NTP_transferase	PF00483.24		RNA metabolism
NEMBOFW57_002492	3	1	1	1	57.1	6.61	2.87	1				x			IMPDH	PF00478.26		RNA metabolism
NEMBOFW57_007658	2	1	1	1	51.7	5.66	2.63	1				x		x	tRNA-synt_His	PF13393.7		RNA metabolism
NEMBOFW57_006453	5	1	1	1	23.3	7.93	2.39	1		x					Ras	PF00071.23		RNA metabolism
NEMBOFW57_002502	3	1	1	1	36.2	9.44	2.25	1					x		HMG_box	PF00505.20		RNA metabolism
NEMBOFW57_007278	1	1	1	1	123.0	5.02	2.2	1			x				MutL_C	PF08676.12		RNA metabolism
NEMBOFW57_006410	14	2	2	2	29.8	7.06	2.15	2	x						S1-P1_nuclease	PF02265.17		RNA metabolism
NEMBOFW57_001351	1	1	1	1	139.0	8.05	2.07	1						x	RdRP	PF05183.13		RNA metabolism
NEMBOFW57_004270	4	1	1	1	34.6	8.95	1.83	1		x					NMT1	PF09084.12		RNA metabolism
NEMBOFW57_001670	4	2	4	2	49.3	5.9	1.78	2		x					DEAD	PF00270.30		RNA metabolism
NEMBOFW57_002925	4	1	1	1	24.2	5.26	1.72	1				x			RraA-like	PF03737.16		RNA metabolism
NEMBOFW57_000212	2	1	1	1	60.7	9.13	1.69	1		x					DEAD	PF00270.30		RNA metabolism
NEMBOFW57_010150	2	1	1	1	41.8	5.95	1.67	1		x					OMPdecase	PF00215.25		RNA metabolism
NEMBOFW57_007475	5	1	2	1	22.8	7.03	1.61	1	x						Ras	PF00071.23		RNA metabolism
NEMBOFW57_000237	1	1	1	1	52.4	7.17	1.61	1		x					RRM_1	PF00076.23		RNA metabolism
NEMBOFW57_001694	2	1	1	1	147.0	5.59	0.0	1	x						RdRP	PF05183.13		RNA metabolism
NEMBOFW57_009941	8	2	3	2	25.0	5.72	0.0	2	x	x					Ras	PF00071.23		RNA metabolism
NEMBOFW57_002870	7	1	1	1	16.2	6.13	0.0	1		x					Ribonuc_L-PSP	PF01042.22		RNA metabolism

NEMBOFW57_010334	1	1	1	1	107.0	6.15	0.0	1		x					CTP_synth_N	PF06418.15		RNA metabolism
NEMBOFW57_010719	55	9	28	9	17.4	9.01	48.21	9	x	x	x	x	x	x	DUF3237	PF11578.9		Unkown function
NEMBOFW57_006192	49	10	20	10	25.0	8.81	46.36	10	x	x	x	x	x	x	DUF3455	PF11937.9		Unkown function
NEMBOFW57_010273	45	14	31	14	37.6	7.93	44.86	14		x					DUF1996	PF09362.11		Unkown function
NEMBOFW57_009140	41	11	23	11	35.1	7.03	37.84	11	x	x	x	x			WD40	PF00400.33		Unkown function
NEMBOFW57_007805	18	8	17	8	48.2	5.33	37.18	8	x	x					DUF4419	PF14388.7		Unkown function
NEMBOFW57_006151	19	6	9	6	42.0	5.03	28.85	6	x	x	x	x	x	x	Ecm33	PF12454.9		Unkown function
NEMBOFW57_007837	20	9	15	9	79.5	5.74	28.81	9	x	x					NAD_binding_10	PF13460.7	predicted protein [Chaetomium globosum CBS 148.51]	Unkown function
NEMBOFW57_002540	25	3	6	3	21.0	7.24	27.24	3	x	x	x	x	x	x	DUF2141	PF09912.10		Unkown function
NEMBOFW57_001430	35	6	14	6	22.9	5.48	25.62	6	x	x	x	x	x		DUF4360	PF14273.7		Unkown function
NEMBOFW57_004124	34	11	12	11	45.1	6.76	23.0	11	x	x	x	x		x	p450	PF00067.23	Putative cytochrome P450 B-class [Podospora comata]	Unkown function
NEMBOFW57_002803	34	3	6	3	20.4	6.15	22.38	3	x	x	x	x	x	x	DUF4360	PF14273.7		Unkown function
NEMBOFW57_003870	7	1	7	1	33.3	5.99	22.18	1	x		x	x	x		NIF3	PF01784.19		Unkown function
NEMBOFW57_006823	20	6	7	6	39.0	6.38	19.91	6		x	x	x	x	x	WSC	PF01822.20		Unkown function
NEMBOFW57_010307	12	6	10	6	43.3	6.13	19.84	6	x		x	x	x		CBM_4_9	PF02018.18		Unkown function
NEMBOFW57_009116	34	6	17	6	22.6	7.69	18.8	6	x	x					DUF4360	PF14273.7		Unkown function
NEMBOFW57_004266	8	4	6	4	80.4	6.57	16.5	4	x	x					DUF3237	PF11578.9		Unkown function
NEMBOFW57_007937	26	3	5	3	21.7	6.99	16.24	3		x		x	x		DUF4360	PF14273.7		Unkown function
NEMBOFW57_009752	14	9	9	9	79.7	7.01	16.14	9	x	x					DUF5703_N	PF18961.1		Unkown function
NEMBOFW57_010878	8	2	4	2	51.1	5.33	15.74	2			x	x	x	x	FAD_binding_4	PF01565.24	hypothetical protein CDV36_006109 [Fusarium kuroshium]	Unkown function
NEMBOFW57_001498	24	4	6	4	22.8	8.76	13.6	4	x	x					CDH-cyt	PF16010.6	hypothetical protein CHGG_06676 [Chaetomium glo-bosum CBS 148.51]	Unkown function

NEMBOFW57_010926	21	7	11	7	36.5	7.46	13.11	7		x					DUF1996	PF09362.11		Unkown function
NEMBOFW57_003149	9	1	4	1	17.1	5.12	12.76	1			x	x		x	DUF3237	PF11578.9		Unkown function
NEMBOFW57_002808	12	2	5	2	28.7	5.5	12.38	2			x	x	x	x	Abhydrolase_6	PF12697.8	alpha/beta-hydrolase [Coni-ochaeta ligniaria NRRL 30616]	Unkown function
NEMBOFW57_008690	33	6	6	6	29.5	5.92	11.52	6	x	x					DUF5127	PF17168.5		Unkown function
NEMBOFW57_002006	28	2	3	2	14.3	5.14	11.47	2			x	x	x	x	DUF3759	PF12585.9		Unkown function
NEMBOFW57_008609	9	6	8	6	66.1	6.09	11.19	6	x	x					DUF4419	PF14388.7		Unkown function
NEMBOFW57_000313	24	5	6	5	27.8	8.82	10.0	5	x	x					Abhydrolase_3	PF07859.14	alpha/beta-hydrolase [Chalara longipes BDJ]	Unkown function
NEMBOFW57_006546	53	5	7	5	21.3	7.61	9.75	5	x	x					DUF4360	PF14273.7		Unkown function
NEMBOFW57_004110	24	4	4	4	19.6	5.63	9.52	4	x	x					DUF1993	PF09351.11		Unkown function
NEMBOFW57_001763	8	4	4	4	63.5	7.68	9.19	4	x		x	x	x	x	FAD_binding_4	PF01565.24	uncharacterized protein THITE_38252 [Thermothielavi-oides terrestris NRRL 8126]	Unkown function
NEMBOFW57_000652	12	6	7	6	66.9	6.4	9.18	6	x		x	x			DUF1996	PF09362.11		Unkown function
NEMBOFW57_009373	7	2	4	2	47.5	5.34	9.16	2	x	x					DUF3237	PF11578.9		Unkown function
NEMBOFW57_002005	4	4	4	4	109.5	5.59	8.7	4	x	x					DUF4246	PF14033.7		Unkown function
NEMBOFW57_006084	6	1	2	1	38.5	7.72	7.79	1			x	x		x	WD40	PF00400.33		Unkown function
NEMBOFW57_008753	16	2	5	2	20.0	7.05	7.55	2		x					TEX13	PF15186.7		Unkown function
NEMBOFW57_010321	15	4	4	4	34.2	5.44	7.08	4	x			x			NAD_binding_10	PF13460.7	hypothetical protein LIP-STDRAFT_3195 [Lipomyces stark-eyi NRRL Y-11557]	Unkown function
NEMBOFW57_006774	6	4	4	4	117.8	6.29	6.84	4		x					FAD_binding_3	PF01494.20	hypothetical protein CHGG_04099 [Chaetomium glo-bosum CBS 148.51]	Unkown function
NEMBOFW57_004125	12	4	8	4	39.2	7.58	6.77	4	x	x					DUF1996	PF09362.11		Unkown function
NEMBOFW57_010297	18	3	5	3	34.1	6.38	6.74	3		x					CBM_1	PF00734.19		Unkown function
NEMBOFW57_008246	18	2	2	2	19.4	5.41	6.32	2	x		x	x			C166	PF17615.3		Unkown function

NEMBOFW57_005518	5	2	2	2	60.3	7.52	6.13	2				x			DUF1688	PF07958.12		Unkown function
NEMBOFW57_009301	27	3	3	3	15.0	6.9	5.36	3	x	x					DUF1323	PF07037.12		Unkown function
NEMBOFW57_007498	5	2	2	2	55.5	6.67	5.1	2				x			FMN_dh	PF01070.19	FMN-dependent dehydrogenase-domain-containing protein [Coni-ochaeta sp. 2T2.1]	Unkown function
NEMBOFW57_001800	3	1	2	1	48.4	9.16	5.03	1				x	x		GRP	PF07172.12		Unkown function
NEMBOFW57_000602	6	2	2	2	39.4	7.52	4.77	2				x			Abhydrolase_1	PF00561.21	alpha/beta-hydrolase [Coni-ochaeta ligniaria NRRL 30616]	Unkown function
NEMBOFW57_005798	4	1	2	1	51.4	5.34	4.48	1			x	x	x	x	DUF89	PF01937.20		Unkown function
NEMBOFW57_006000	1	1	2	1	94.5	6.07	4.29	1		x					DUF1793	PF08760.12		Unkown function
NEMBOFW57_006602	20	6	6	6	40.4	7.62	4.25	6		x					Reticulon	PF02453.18		Unkown function
NEMBOFW57_004017	14	3	3	3	44.5	7.49	4.11	3		x					CBM_1	PF00734.19	hypothetical protein CHGG_03878 [Chaetomium glo-bosum CBS 148.51]	Unkown function
NEMBOFW57_004364	3	2	2	2	78.2	8.43	4.06	2		x					AMP-binding	PF00501.29	hypothetical protein CHGG_10337 [Chaetomium glo-bosum CBS 148.51]	Unkown function
NEMBOFW57_007919	10	2	2	2	26.4	6.34	3.87	2	x	x					DUF4360	PF14273.7		Unkown function
NEMBOFW57_005764	3	2	2	2	94.2	6.46	3.76	2		x					Ytp1	PF10355.10		Unkown function
NEMBOFW57_003133	2	1	1	1	68.5	5.47	3.27	1				x			TPR_14	PF13428.7		Unkown function
NEMBOFW57_006165	29	3	6	3	12.8	5.82	3.06	3			x	x			YCII	PF03795.15		Unkown function
NEMBOFW57_006968	9	1	2	1	15.4	5.92	3.01	1	x	x	x				SH3_3	PF08239.12		Unkown function
NEMBOFW57_009787	14	1	1	1	13.8	4.63	3.0	1				x			DUF866	PF05907.14		Unkown function
NEMBOFW57_005391	8	1	1	1	20.9	6.1	2.77	1				x			Ham1p_like	PF01725.17		Unkown function
NEMBOFW57_006144	10	1	1	1	17.7	9.57	2.65	1		x					DUF1761	PF08570.11		Unkown function
NEMBOFW57_004890	19	2	2	2	15.4	5.27	2.39	2		x					SNRNP27	PF08648.13		Unkown function
NEMBOFW57_008691	9	1	1	1	25.9	4.7	2.25	1	x	x					DUF1793	PF08760.12		Unkown function

NEMBOFW57_000843	11	1	1	1	16.3	7.03	2.23	1				x			UPF0047	PF01894.18		Unkown function
NEMBOFW57_002636	3	1	1	1	44.0	7.36	2.15	1		x					YchF-GTPase_C	PF06071.14		Unkown function
NEMBOFW57_000291	3	1	1	1	66.8	7.8	2.15	1						x	WD40	PF00400.33	hypothetical protein CHGG_02164 [Chaetomium globosum CBS 148.51]	Unkown function
NEMBOFW57_006647	1	1	2	1	233.4	6.39	2.01	1		x					DUF1729	PF08354.11		Unkown function
NEMBOFW57_007833	5	1	1	1	23.5	5.29	1.98	1			x	x			DUF3123	PF11321.9		Unkown function
NEMBOFW57_005549	3	1	2	1	34.6	7.25	1.95	1	x						DUF3567	PF12091.9		Unkown function
NEMBOFW57_001359	3	1	1	1	38.0	8.0	1.94	1		x					LysM	PF01476.21	LysM domain-containing protein [Colletotrichum higginsianum IMI 349063]	Unkown function
NEMBOFW57_007163	6	2	2	2	40.1	6.87	1.84	2		x					VCBS	PF13517.7		Unkown function
NEMBOFW57_002902	7	3	3	3	65.1	5.67	1.76	3		x					TPR_1	PF00515.29		Unkown function
NEMBOFW57_002639	7	1	1	1	19.2	6.4	1.75	1		x					DUF1441	PF07278.12		Unkown function
NEMBOFW57_006923	6	2	2	2	27.6	5.22	1.74	2	x						WSC	PF01822.20		Unkown function
NEMBOFW57_000788	3	1	1	1	63.6	5.25	1.66	1		x					HEAT	PF02985.23		Unkown function
NEMBOFW57_002381	11	2	2	2	20.9	7.69	1.63	2	x						Abhydrolase_8	PF06259.13	uncharacterized protein THITE_2170113 [Thermothielavioides terrestris NRRL 8126]	Unkown function
NEMBOFW57_008613	10	2	2	2	36.7	8.7	0.0	2	x						Beta_helix	PF13229.7	hypothetical protein M434DRAFT_16125 [Hypoxylon sp. CO27-5]	Unkown function
NEMBOFW57_003992	2	1	1	1	52.5	4.59	0.0	1	x						Beta_helix	PF13229.7	hypothetical protein P171DRAFT_353961 [Karstenula rhodostoma CBS 690.94]	Unkown function
NEMBOFW57_009596	1	1	1	1	79.8	4.91	0.0	1	x						DUF4246	PF14033.7		Unkown function
NEMBOFW57_004009	2	1	1	1	45.8	9.39	0.0	1	x						Caskin-Pro-rich	PF16907.6		Unkown function
NEMBOFW57_002086	12	3	3	3	38.1	7.2	0.0	3		x					DUF3712	PF12505.9		Unkown function

NEMBOFW57_006994	17	2	2	2	24.0	5.3	0.0	2		x					DUF1349	PF07081.12		Unkown function
NEMBOFW57_000580	4	1	1	1	33.4	5.24	0.0	1	x	x					SGL	PF08450.13		Unkown function
NEMBOFW57_003335	3	1	2	1	45.2	7.93	0.0	1		x					DUF1772	PF08592.12		Unkown function
NEMBOFW57_003146	4	1	1	1	33.6	9.64	0.0	1	x	x					Band_7	PF01145.26		Unkown function
NEMBOFW57_010708	6	1	1	1	27.8	5.6	0.0	1		x					DUF1996	PF09362.11		Unkown function
NEMBOFW57_004258	3	1	1	1	28.4	6.86	0.0	1		x					DeoC	PF01791.10		Unkown function
NEMBOFW57_000957	1	1	1	1	173.7	5.81	0.0	1			x				HEAT_2	PF13646.7		Unkown function
NEMBOFW57_006427	31	5	9	5	25.4	6.67	15.36	5	x	x					CBM_4_9	PF02018.18		Unkown function

Supplementary Table S6. Proteins of the fungal isolate FW57 induced on maize leaves (MZ) or sugarcane bagasse (SCB) as part of the time-course analysis. Measurements were taken on days 7, 14, 21 and 28. If a protein was identified under a certain condition at a given time point it is marked as cross (x). The proteins were identified by in-solution tryptic digestion and LC-MS/MS analysis. The accession number, coverage (%), number of peptides (# peptides), peptide-to-spectrum matches (# PSMs), number of unique peptides (# Unique Peptides), molecular weight in kDa (MW [kDa]), calculated isoelectric point (calc. pI), number of Peptides Sequest HT (# Peptides Sequest HT) were compared with the automated translation of the genome of the fungal isolate *Staphylotrichum longicolleum* DSM105789. Pfam annotation was used for function annotation.

Accession number	SCB				MZ				Coverage [%]	# Peptides	# PSMs	# Unique Peptides	MW [kDa]	calc. pI	# Peptides Sequest HT	Function
	7	14	21	28	7	14	21	28								
NEMBOFW57_000134	X					X	X	X	8	3	11	3	60.6	7.93	22.93	CAZYME
NEMBOFW57_000164	X	X	X	X	X	X	X	X	11	4	15	4	41.7	5.35	40.24	CAZYME
NEMBOFW57_000190	X								1	1	2	1	92.2	6.3	5.55	CAZYME
NEMBOFW57_000278	X								4	1	1	1	31.7	6.06	2.7	CAZYME
NEMBOFW57_000322	X	X	X						9	5	9	5	53.9	5.44	23.15	CAZYME
NEMBOFW57_000338	X						X	X	7	2	5	2	52.7	6.28	12.14	CAZYME
NEMBOFW57_000575	X		X			X		X	12	3	8	3	37.5	5.3	19.94	CAZYME
NEMBOFW57_000683	X	X	X	X		X			7	2	6	2	29.3	6.43	13.99	CAZYME
NEMBOFW57_000887	X	X	X	X		X	X	X	3	2	8	2	74.9	8.51	22.5	CAZYME

NEM-BOFW57_001011	X	X	X	X					6	5	14	5	87.8	8.12	29.9	CAZYME
NEM-BOFW57_001044	X	X	X	X	X		X	X	24	5	29	5	32.9	7.18	101.51	CAZYME
NEM-BOFW57_001114	X								2	1	1	1	82	5.33	3.34	CAZYME
NEM-BOFW57_001172	X								6	1	1	1	25.8	6.39	3.18	CAZYME
NEM-BOFW57_001193	X								2	1	2	1	40.3	7.81	3.85	CAZYME
NEM-BOFW57_001269	X			X			X	X	6	2	4	2	39	5.88	9.75	CAZYME
NEM-BOFW57_001288	X	X		X		X	X	X	7	4	13	4	64.8	7.61	46.39	CAZYME
NEM-BOFW57_001310	X	X	X	X	X	X	X	X	7	2	13	2	51.2	7.8	40.51	CAZYME
NEM-BOFW57_001352	X	X	X	X		X	X	X	7	4	18	4	67.4	8.38	52.01	CAZYME
NEM-BOFW57_001439	X	X	X	X		X	X	X	13	2	19	2	27.6	8.91	39.71	CAZYME
NEM-BOFW57_001466	X	X	X	X		X	X	X	13	4	21	4	33.3	7.65	70.3	CAZYME
NEM-BOFW57_001526	X								9	3	5	3	37.2	5.06	12.71	CAZYME
NEM-BOFW57_001853	X	X					X	X	2	2	4	2	145.4	5.64	9.37	CAZYME
NEM-BOFW57_002037	X	X		X			X	X	1	1	4	1	58	5.99	8.77	CAZYME
NEM-BOFW57_002054	X								7	2	5	2	25.9	8.09	11.12	CAZYME
NEM-BOFW57_002223	X								3	1	1	1	38.4	8.79	2.58	CAZYME
NEM-BOFW57_002472	X								1	1	1	1	78.1	6.25	2.27	CAZYME
NEM-BOFW57_002500	X	X	X	X			X	X	20	5	28	5	23.7	7.42	74.35	CAZYME
NEM-BOFW57_002874	X	X	X	X			X	X	5	3	9	3	71.4	6.2	21.59	CAZYME
NEM-BOFW57_002909	X	X					X	X	4	2	5	2	60.5	5.48	11.55	CAZYME
NEM-BOFW57_002998	X								3	1	1	1	37.4	9.09	2.13	CAZYME
NEM-BOFW57_003080	X						X	X	14	4	4	4	49.2	4.78	6.57	CAZYME
NEM-BOFW57_003104	X	X	X	X		X	X	X	2	1	12	1	104.3	5.4	38.97	CAZYME

NEM-BOFW57_003169	X	X	X	X					6	4	15	4	64.9	5.91	32.89	CAZYME
NEM-BOFW57_003451	X						X	X	4	2	3	2	77.8	5.94	6.45	CAZYME
NEM-BOFW57_003635	X		X						2	1	3	1	41.4	7.28	6.98	CAZYME
NEM-BOFW57_003714	X	X	X	X		X	X	X	20	8	23	8	51.3	7.52	52.9	CAZYME
NEM-BOFW57_003741	X								4	1	2	1	44.1	8.7	6.67	CAZYME
NEM-BOFW57_003955	X								6	2	6	2	45.1	8.05	16.3	CAZYME
NEM-BOFW57_004002	X					X	X	X	7	2	4	2	47.4	8.44	7.65	CAZYME
NEM-BOFW57_004062	X	X	X	X		X	X	X	4	1	19	1	41.4	8.41	73.22	CAZYME
NEM-BOFW57_004115	X							X	8	3	6	3	54.2	7.61	16.56	CAZYME
NEM-BOFW57_004293	X	X							4	1	3	1	39.7	5.8	10.99	CAZYME
NEM-BOFW57_004303	X	X	X	X				X	11	3	8	3	36.3	7.2	20.43	CAZYME
NEM-BOFW57_004374	X	X	X	X		X	X	X	5	5	26	5	97.2	9	78.45	CAZYME
NEM-BOFW57_004454	X	X		X			X		4	2	3	2	51.3	5.4	2.32	CAZYME
NEM-BOFW57_004513	X								3	1	1	1	31.2	8.24	2.44	CAZYME
NEM-BOFW57_004556	X								5	1	2	1	26.9	6.52	5.78	CAZYME
NEM-BOFW57_004659	X								4	1	2	1	36.1	9.07	5.01	CAZYME
NEM-BOFW57_005018	X								9	1	1	1	26	8.31	2.18	CAZYME
NEM-BOFW57_005380	X						X	X	5	3	4	3	103.3	7.42	8.6	CAZYME
NEM-BOFW57_005402	X								1	1	2	1	76.4	6.74	4.16	CAZYME
NEM-BOFW57_005755	X	X		X			X	X	6	2	8	2	57	5.02	28.89	CAZYME
NEM-BOFW57_005776	X								3	1	1	1	45.6	7.02	2.09	CAZYME
NEM-BOFW57_006022	X								2	1	1	1	40.6	7.49	2.48	CAZYME
NEM-BOFW57_006728	X	X	X	X		X	X	X	23	5	17	5	35.4	8.84	42.03	CAZYME

NEM-BOFW57_006928	X	X	X	X					3	1	6	1	47	4.7	15.04	CAZYME
NEM-BOFW57_007038	X						X	X	8	2	3	2	38.1	6.3	6.75	CAZYME
NEM-BOFW57_007083	X	X	X	X		X	X		9	3	7	3	49.5	8.22	14.09	CAZYME
NEM-BOFW57_007659	X						X	X	3	2	3	2	69.2	7.06	6.78	CAZYME
NEM-BOFW57_007691	X	X	X	X		X	X	X	16	6	21	6	42.6	6.7	90.37	CAZYME
NEM-BOFW57_007704	X	X	X	X					18	7	26	7	56.9	5.78	72.72	CAZYME
NEM-BOFW57_007800	X								3	1	1	1	33.5	8.03	1.73	CAZYME
NEM-BOFW57_007876	X	X							5	1	3	1	27.2	8.06	9.68	CAZYME
NEM-BOFW57_007945	X	X							4	2	3	2	61.2	6.13	8.4	CAZYME
NEM-BOFW57_008032	X								2	1	1	1	54.4	8.02	0	CAZYME
NEM-BOFW57_008041	X	X	X	X		X	X	X	1	1	6	1	100.9	5.92	22.05	CAZYME
NEM-BOFW57_008270	X	X	X	X		X	X	X	5	2	30	2	55.3	7.81	90.87	CAZYME
NEM-BOFW57_008456	X								4	1	1	1	48.9	5.31	0	CAZYME
NEM-BOFW57_008500	X		X	X		X	X	X	9	2	7	2	39.2	5.39	16.83	CAZYME
NEM-BOFW57_008512	X	X				X	X	X	2	1	3	1	62.6	6.49	7.08	CAZYME
NEM-BOFW57_008527	X							X	3	3	5	3	89.2	6.48	11.15	CAZYME
NEM-BOFW57_008581	X					X	X	X	21	3	13	3	35.4	8.46	44.85	CAZYME
NEM-BOFW57_008598	X	X	X	X		X	X	X	18	4	23	4	39.7	7.44	66	CAZYME
NEM-BOFW57_008624	X	X	X	X		X	X	X	9	5	49	5	82	6.28	153.31	CAZYME
NEM-BOFW57_008641	X	X	X	X		X	X	X	9	5	100	5	42.2	6.27	347.29	CAZYME
NEM-BOFW57_008720	X	X	X	X					5	1	9	1	23.2	5.5	21.03	CAZYME
NEM-BOFW57_008747	X								2	1	1	1	77.5	5.34	0	CAZYME
NEM-BOFW57_009003	X		X						3	1	4	1	34.2	7.37	12.07	CAZYME

NEM-BOFW57_009085	X	X	X	X					11	3	9	3	25.7	7.9	19.44	CAZYME
NEM-BOFW57_009152	X	X	X	X		X	X	X	10	7	38	7	93.9	7.43	112.72	CAZYME
NEM-BOFW57_009223	X								2	1	1	1	85.4	5.48	3.54	CAZYME
NEM-BOFW57_009578	X	X	X	X		X		X	14	3	10	3	27.2	7.83	29.58	CAZYME
NEM-BOFW57_009579	X								3	1	1	1	51.8	6.33	2.36	CAZYME
NEM-BOFW57_009841	X							X	5	2	3	2	56.2	7.94	6.18	CAZYME
NEM-BOFW57_010246	X	X	X	X			X	X	7	2	9	2	58.2	7.17	26.09	CAZYME
NEM-BOFW57_010290	X	X	X	X			X	X	19	5	19	5	35.2	7.83	44.83	CAZYME
NEM-BOFW57_010291	X	X	X	X	X	X	X	X	60	9	71	9	17.3	5.3	235.43	CAZYME
NEM-BOFW57_010319	X	X	X	X	X	X	X	X	19	8	47	8	63.3	6.84	163.69	CAZYME
NEM-BOFW57_010602	X	X					X	X	4	3	6	3	62.8	8.13	14.43	CAZYME
NEM-BOFW57_010955	X								8	1	1	1	31.7	7.94	0	CAZYME
NEM-BOFW57_010962	X								4	1	1	1	45.2	7.34	2.74	CAZYME
NEM-BOFW57_001303	X				X				4	1	1	1	28.4	5.87	2.56	Energy metabolism
NEM-BOFW57_001718	X								5	1	1	1	34.6	7.78	1.85	Energy metabolism
NEM-BOFW57_001745	X	X	X	X	X	X	X	X	21	7	20	7	35.1	5.66	52.72	Energy metabolism
NEM-BOFW57_002476	X								3	1	2	1	57	6.8	5.65	Energy metabolism
NEM-BOFW57_002584	X				X				3	1	1	1	46.1	5.31	2.1	Energy metabolism
NEM-BOFW57_002646	X								2	1	1	1	59.6	6.49	2.82	Energy metabolism
NEM-BOFW57_003315	X								5	1	1	1	33.8	5.3	2.45	Energy metabolism
NEM-BOFW57_003985	X								2	1	1	1	57.9	6.57	2.27	Energy metabolism
NEM-BOFW57_004223	X	X	X	X					11	3	9	3	36.5	5.07	16.41	Energy metabolism
NEM-BOFW57_004369	X				X				3	2	2	2	98.8	5.22	4.76	Energy metabolism

NEM-BOFW57_004456	X								2	1	1	1	72.2	8.35	2.3	Energy metabolism
NEM-BOFW57_004828	X	X	X				X		4	2	6	2	40.4	5.83	22.06	Energy metabolism
NEM-BOFW57_007133	X		X					X	6	4	6	4	60.8	6.7	9.1	Energy metabolism
NEM-BOFW57_008695	X								2	1	1	1	52.3	7.5	2.2	Energy metabolism
NEM-BOFW57_008876	X								2	1	1	1	32.9	5.99	1.81	Energy metabolism
NEM-BOFW57_009613	X	X	X	X		X	X	X	19	5	19	5	41.6	6.01	47.5	Energy metabolism
NEM-BOFW57_009796	X						X	X	7	2	4	2	44.3	5.92	9.51	Energy metabolism
NEM-BOFW57_009946	X								8	2	2	2	36.2	7.39	4.07	Energy metabolism
NEM-BOFW57_010857	X								7	1	1	1	16.5	8.38	2.7	Energy metabolism
NEM-BOFW57_002516	X	X		X				X	2	1	4	1	69.1	5.19	9.67	Lipid metabolism
NEM-BOFW57_005494	X								2	1	1	1	46	7.08	2.23	Lipid metabolism
NEM-BOFW57_006771	X						X	X	4	1	3	1	70.5	7.36	7.94	Lipid metabolism
NEM-BOFW57_010938	X			X					7	2	6	2	39.3	5.35	17.46	Lipid metabolism
NEM-BOFW57_000156	X	X	X	X		X	X	X	19	4	49	4	26.5	6.67	131.36	NON CONSERVED PATTERN
NEM-BOFW57_001211	X	X	X	X				X	10	2	6	2	23.7	8.53	15.69	NON CONSERVED PATTERN
NEM-BOFW57_002919	X	X	X	X					12	1	7	1	19.7	7.42	20.68	NON CONSERVED PATTERN
NEM-BOFW57_003604	X								4	1	2	1	18.9	7.71	4.57	NON CONSERVED PATTERN
NEM-BOFW57_003820	X								1	1	2	1	135.7	6.39	1.88	Non conserved pattern
NEM-BOFW57_004728	X		X				X	X	6	1	4	1	14.6	5.24	5.91	NON CONSERVED PATTERN
NEM-BOFW57_004778	X								6	1	1	1	17.1	8.9	1.98	NON CONSERVED PATTERN
NEM-BOFW57_005070	X	X				X	X	X	2	5	10	5	461.1	4.11	15.16	NON CONSERVED PATTERN
NEM-BOFW57_005291	X	X	X	X		X	X	X	26	2	21	2	10.2	7.02	64.48	NON CONSERVED PATTERN
NEM-BOFW57_005507	X	X							7	1	3	1	22.1	7.21	7.95	NON CONSERVED PATTERN

NEM-BOFW57_005666	X								5	1	1	1	37.8	5.58	1.64	NON CONSERVED PATTERN
NEM-BOFW57_007650	X	X			X				15	2	3	2	26.7	4.67	10.15	NON CONSERVED PATTERN
NEM-BOFW57_007828	X								3	1	2	1	32.5	6.98	3.75	NON CONSERVED PATTERN
NEM-BOFW57_008003	X								3	1	2	1	44.6	7.5	3.85	NON CONSERVED PATTERN
NEM-BOFW57_009268	X					X	X		4	1	3	1	32.1	4.96	6.6	NON CONSERVED PATTERN
NEM-BOFW57_009312	X								4	1	1	1	28.3	6.07	2.13	NON CONSERVED PATTERN
NEM-BOFW57_000071	X	X			X	X	X	X	10	2	9	2	21.6	5.16	23.09	Other biological process
NEM-BOFW57_000397	X								7	1	5	1	18.7	4.56	14.08	Other biological process
NEM-BOFW57_000673	X								3	2	2	2	90.5	5.17	5.12	Other biological process
NEM-BOFW57_000696	X								6	1	1	1	19.4	4.67	2.44	Other biological process
NEM-BOFW57_000919	X	X							3	2	2	2	86.2	6.6	3.66	Other biological process
NEM-BOFW57_001057	X								5	1	2	1	26	6.09	4.54	Other biological process
NEM-BOFW57_001171	X								10	2	2	2	23.6	5.07	2.84	Other biological process
NEM-BOFW57_001387	X								9	1	1	1	22.3	5.03	3.36	Other biological process
NEM-BOFW57_001731	X	X	X	X				X	11	3	6	3	28.1	6.54	10.97	Other biological process
NEM-BOFW57_001992	X								10	1	1	1	16.9	6.34	2.25	Other biological process
NEM-BOFW57_002503	X								1	1	1	1	90.1	6.16	2.15	Other biological process
NEM-BOFW57_002862	X								2	1	1	1	70.4	4.56	3.4	Other biological process
NEM-BOFW57_002896	X	X	X	X			X	X	14	1	6	1	12.2	6.55	14.55	Other biological process
NEM-BOFW57_003025	X	X							3	1	2	1	48.9	6.67	5.54	Other biological process
NEM-BOFW57_003065	X								3	1	1	1	63.3	5.14	0	Other biological process
NEM-BOFW57_003346	X	X	X	X			X		8	1	7	1	23.7	5.45	21.75	Other biological process
NEM-BOFW57_003460	X								9	2	2	2	61	5	5.6	Other biological process

NEM-BOFW57_003663	X								7	1	1	1	23	7.56	2.15	Other biological process
NEM-BOFW57_003675	X								3	1	1	1	41.3	5.35	2.39	Other biological process
NEM-BOFW57_003920	X								5	1	1	1	22	4.51	2.52	Other biological process
NEM-BOFW57_003945	X								9	1	1	1	32.2	4.55	3.18	Other biological process
NEM-BOFW57_004279	X	X	X	X					9	2	9	2	15.3	6.73	31.16	Other biological process
NEM-BOFW57_004571	X								4	1	1	1	22.7	5.1	2.12	Other biological process
NEM-BOFW57_004626	X	X	X	X		X	X	X	15	4	15	4	46.4	5.05	41.31	Other biological process
NEM-BOFW57_004688	X	X							5	1	3	1	31	6.37	12.49	Other biological process
NEM-BOFW57_004791	X	X							3	2	4	2	42.5	6.24	9.67	Other biological process
NEM-BOFW57_004820	X	X	X	X				X	22	2	9	2	16.1	5.62	15.85	Other biological process
NEM-BOFW57_004990	X								13	1	2	1	13	5.6	3.28	Other biological process
NEM-BOFW57_005563	X								7	1	2	1	17.6	9.31	4.74	Other biological process
NEM-BOFW57_005717	X			X				X	20	2	4	2	20	4.79	6.55	Other biological process
NEM-BOFW57_005781	X	X	X	X		X	X	X	11	1	8	1	14.5	6.27	23.84	Other biological process
NEM-BOFW57_005790	X	X	X						6	1	3	1	18.5	4.28	8.16	Other biological process
NEM-BOFW57_005811	X	X		X	X	X	X		8	4	17	4	79.5	4.97	51.97	Other biological process
NEM-BOFW57_005874	X				X				7	1	2	1	17	4.27	4.11	Other biological process
NEM-BOFW57_005989	X	X	X	X	X		X		6	3	12	3	41.6	5.69	27.48	Other biological process
NEM-BOFW57_006563	X	X	X	X	X	X	X	X	38	3	25	3	11.5	4.97	66.52	Other biological process
NEM-BOFW57_006834	X								9	1	1	1	21.6	5.49	2.67	Other biological process
NEM-BOFW57_007088	X								11	3	3	3	46.6	5.38	9.07	Other biological process
NEM-BOFW57_007284	X								2	1	1	1	72.5	8.47	2.19	Other biological process
NEM-BOFW57_007379	X	X		X		X	X	X	8	2	9	2	40.4	6.44	19.06	Other biological process

NEM-BOFW57_007484	X	X							15	2	3	2	14.1	6.15	6.72	Other biological process
NEM-BOFW57_007559	X								2	1	1	1	85.7	5.1	2.3	Other biological process
NEM-BOFW57_007610	X								6	1	1	1	22.1	4.93	2.65	Other biological process
NEM-BOFW57_007655	X	X		X		X	X	X	4	2	11	2	81.4	8.35	34.9	Other biological process
NEM-BOFW57_007777	X								4	2	2	2	49.1	6.18	3.96	Other biological process
NEM-BOFW57_007982	X	X	X	X	X	X	X	X	12	3	9	3	27.6	6.16	18.9	Other biological process
NEM-BOFW57_008011	X	X		X			X	X	12	3	7	3	42.4	7.49	19.08	Other biological process
NEM-BOFW57_008349	X								1	1	1	1	199.7	5.06	0	Other biological process
NEM-BOFW57_008390	X								1	1	1	1	139.9	7.8	1.72	Other biological process
NEM-BOFW57_008393	X	X	X	X	X	X	X	X	13	7	22	7	72.2	5.03	45.16	Other biological process
NEM-BOFW57_008685	X	X				X	X	X	7	3	7	3	88.2	4.53	21.76	Other biological process
NEM-BOFW57_008807	X								4	1	1	1	41	5.39	0	Other biological process
NEM-BOFW57_009102	X			X			X	X	16	2	5	2	15.6	5.39	16.05	Other biological process
NEM-BOFW57_009162	X	X		X		X	X	X	6	2	7	2	40.8	5.33	8.78	Other biological process
NEM-BOFW57_009228	X								15	2	4	2	12.1	4.3	8.8	Other biological process
NEM-BOFW57_009732	X	X	X		X		X	X	13	6	17	6	62.4	6.02	36.37	Other biological process
NEM-BOFW57_009733	X						X		15	1	1	1	17.9	7.18	3.58	Other biological process
NEM-BOFW57_010231	X			X	X	X	X		11	6	14	6	70.9	5.21	33.26	Other biological process
NEM-BOFW57_010263	X								3	1	1	1	45.9	5.64	2.13	Other biological process
NEM-BOFW57_010280	X						X	X	6	1	5	1	21.5	6.29	13.68	Other biological process
NEM-BOFW57_010451	X								3	1	1	1	39.2	7.14	2.25	Other biological process
NEM-BOFW57_010598	X	X							9	2	3	2	28.9	6.09	5.44	Other biological process
NEM-BOFW57_010621	X	X							4	1	2	1	33.3	6.37	6.1	Other biological process

NEM-BOFW57_010755	X								10	3	3	3	33.1	7.11	7.13	Other biological process
NEM-BOFW57_010939	X	X	X						10	2	4	2	19.7	5.38	8.13	Other biological process
NEM-BOFW57_010943	X						X		1	1	2	1	123.6	7.33	5.2	Other biological process
NEM-BOFW57_000013	X	X		X					3	2	7	2	84.4	6.07	9.34	Oxidation-reduction
NEM-BOFW57_001478	X	X	X	X					6	1	4	1	25.6	6.4	10.08	Oxidation-reduction
NEM-BOFW57_001747	X	X	X	X		X	X		26	3	14	3	15.9	6.52	53.14	Oxidation-reduction
NEM-BOFW57_002578	X	X	X	X	X	X	X	X	24	9	22	9	55.1	4.86	55.07	Oxidation-reduction
NEM-BOFW57_002948	X	X		X					5	1	3	1	24.3	6.64	7.32	Oxidation-reduction
NEM-BOFW57_003058	X		X						3	2	3	2	72.9	5.72	2.49	Oxidation-reduction
NEM-BOFW57_003365	X		X						11	2	4	2	36.3	5.9	12.4	Oxidation-reduction
NEM-BOFW57_003548	X	X			X		X		3	1	4	1	40.1	6.68	10.85	Oxidation-reduction
NEM-BOFW57_003641	X								3	1	2	1	39.3	7.21	4.61	Oxidation-reduction
NEM-BOFW57_004388	X								4	1	1	1	42.8	5.59	3.45	Oxidation-reduction
NEM-BOFW57_005608	X	X	X	X		X	X	X	5	3	10	3	65.9	7.31	24.93	Oxidation-reduction
NEM-BOFW57_006149	X								5	1	1	1	29.9	5.91	2.71	Oxidation-reduction
NEM-BOFW57_006984	X								3	1	1	1	33	6.33	2.13	Oxidation-reduction
NEM-BOFW57_007852	X	X	X						5	1	5	1	46.2	6.61	7.45	Oxidation-reduction
NEM-BOFW57_010467	X	X	X		X	X	X		13	4	17	4	46.2	6.86	35.33	Oxidation-reduction
NEM-BOFW57_000203	X	X	X	X					8	2	9	2	20.3	9.8	28.23	Protein metabolism
NEM-BOFW57_000930	X								16	1	1	1	17.1	8.22	0	Protein metabolism
NEM-BOFW57_001610	X	X		X	X				4	2	5	2	49.9	9.13	12.74	Protein metabolism
NEM-BOFW57_001844	X								6	1	1	1	18	11.39	2.37	Protein metabolism
NEM-BOFW57_001858	X	X							8	1	3	1	15.9	10.67	6.63	Protein metabolism

NEM-BOFW57_001977	X		X	X					8	2	7	2	41	8	17.4	Protein metabolism
NEM-BOFW57_002599	X								4	1	1	1	30.9	5.99	2.24	Protein metabolism
NEM-BOFW57_002628	X								4	1	1	1	25.2	4.58	1.99	Protein metabolism
NEM-BOFW57_002748	X	X							4	1	2	1	19.9	10.13	3.61	Protein metabolism
NEM-BOFW57_002751	X								1	1	1	1	131.2	6.38	2.31	Protein metabolism
NEM-BOFW57_002891	X								9	2	3	2	20.4	6.79	7.68	Protein metabolism
NEM-BOFW57_003164	X								15	1	1	1	12.2	10.86	0	Protein metabolism
NEM-BOFW57_004447	X	X	X						6	2	5	2	46.1	5.67	11.78	Protein metabolism
NEM-BOFW57_005066	X								7	1	1	1	15.1	9.47	2.45	Protein metabolism
NEM-BOFW57_005179	X								9	1	1	1	12	11.62	2.03	Protein metabolism
NEM-BOFW57_005469	X								7	2	3	2	48.9	6.32	9.5	Protein metabolism
NEM-BOFW57_005475	X	X	X						10	2	5	2	22.6	7.65	16.38	Protein metabolism
NEM-BOFW57_005849	X	X							5	1	2	1	20.8	10.43	1.72	Protein metabolism
NEM-BOFW57_005858	X								3	1	1	1	44.7	7.44	2.43	Protein metabolism
NEM-BOFW57_007291	X	X	X	X					6	1	5	1	30.9	5.26	14.89	Protein metabolism
NEM-BOFW57_007908	X	X	X	X					2	1	6	1	49.9	5.72	17.39	Protein metabolism
NEM-BOFW57_008913	X	X		X					4	1	3	1	29.4	10.27	9.1	Protein metabolism
NEM-BOFW57_009144	X								2	1	1	1	59.6	9.14	2.37	Protein metabolism
NEM-BOFW57_009798	X								3	1	1	1	27.2	10.52	1.79	Protein metabolism
NEM-BOFW57_000332	X	X	X	X					5	2	6	2	52.1	6.24	21.74	Proteolysis
NEM-BOFW57_000405	X	X			X				4	1	3	1	29.5	7.4	7.23	Proteolysis
NEM-BOFW57_001262	X	X							4	3	6	3	75.3	6.52	14.29	Proteolysis
NEM-BOFW57_001330	X	X	X	X				X	4	2	7	2	80.5	6.76	17.89	Proteolysis

NEM-BOFW57_001765	X			X					2	1	2	1	57.4	6.61	6.52	Proteolysis
NEM-BOFW57_002765	X	X							5	1	2	1	30.7	5.12	6.06	Proteolysis
NEM-BOFW57_003380	X								2	1	1	1	57.1	6.23	1.93	Proteolysis
NEM-BOFW57_004414	X								3	1	1	1	56.6	6.74	3.2	Proteolysis
NEM-BOFW57_005010	X								2	1	3	1	125.6	5.38	7.35	Proteolysis
NEM-BOFW57_009155	X								3	1	1	1	45.4	6.33	2.05	Proteolysis
NEM-BOFW57_009314	X								2	1	1	1	99.6	5.57	2.43	Proteolysis
NEM-BOFW57_002405	X				X			X	44	3	3	3	12	7.01	7.38	RNA metabolism
NEM-BOFW57_002745	X	X		X	X				4	2	4	2	59.6	9.28	10.94	RNA metabolism
NEM-BOFW57_002870	X								7	1	1	1	16.2	6.13	0	RNA metabolism
NEM-BOFW57_003136	X	X	X				X		9	3	9	3	29.3	5.48	23.82	RNA metabolism
NEM-BOFW57_005292	X			X		X	X	X	23	3	9	3	16.7	7.39	19.89	RNA metabolism
NEM-BOFW57_006545	X	X	X	X			X	X	10	3	13	3	38.7	5.33	30.07	RNA metabolism
NEM-BOFW57_006564	X								2	1	1	1	40.1	8.09	1.65	RNA metabolism
NEM-BOFW57_007251	X	X	X	X		X			11	4	13	4	37.7	6.05	29.16	RNA metabolism
NEM-BOFW57_008846	X	X			X	X	X		4	2	3	2	29.4	6.62	4.85	RNA metabolism
NEM-BOFW57_008893	X	X		X		X	X		2	1	4	1	82.6	6.07	7.86	RNA metabolism
NEM-BOFW57_010061	X								3	1	1	1	47.3	6.92	3.04	RNA metabolism
NEM-BOFW57_010510	X	X	X	X	X		X	X	7	3	10	3	54	8.16	28.82	RNA metabolism
NEM-BOFW57_010526	X								3	1	1	1	88.2	7.09	4.38	RNA metabolism
NEM-BOFW57_000313	X								3	1	1	1	27.8	8.82	2.08	Unkown function
NEM-BOFW57_000580	X								13	3	4	3	33.4	5.24	8.04	Unkown function
NEM-BOFW57_000652	X								2	1	2	1	66.9	6.4	4.88	Unkown function

NEM-BOFW57_001498	X	X	X	X			X	X	6	1	11	1	22.8	8.76	27.45	Unkown function
NEM-BOFW57_002441	X								1	1	1	1	102	9.8	0	Unkown function
NEM-BOFW57_002639	X		X						7	1	2	1	19.2	6.4	2.85	Unkown function
NEM-BOFW57_002894	X								6	1	1	1	24.7	3.98	0	Unkown function
NEM-BOFW57_002902	X		X	X	X	X		X	5	2	5	2	65.1	5.67	13.72	Unkown function
NEM-BOFW57_003004	X								5	1	1	1	44.4	5.16	3.58	Unkown function
NEM-BOFW57_003036	X								4	1	1	1	51.4	4.86	4.26	Unkown function
NEM-BOFW57_004110	X								5	1	1	1	19.6	5.63	2.01	Unkown function
NEM-BOFW57_004124	X		X						5	2	2	2	45.1	6.76	2.19	Unkown function
NEM-BOFW57_004125	X	X	X						3	1	4	1	39.2	7.58	4.47	Unkown function
NEM-BOFW57_004266	X	X		X			X		2	1	3	1	80.4	6.57	11.71	Unkown function
NEM-BOFW57_004890	X	X	X	X			X		24	4	7	4	15.4	5.27	22.19	Unkown function
NEM-BOFW57_005716	X	X							4	1	4	1	31.5	5.66	10.05	Unkown function
NEM-BOFW57_006151	X								2	1	2	1	42	5.03	6.15	Unkown function
NEM-BOFW57_006427	X							X	8	1	1	1	25.4	6.67	2.24	Unkown function
NEM-BOFW57_007805	X								2	1	2	1	48.2	5.33	4.63	Unkown function
NEM-BOFW57_007919	X	X		X				X	10	2	6	2	26.4	6.34	16.11	Unkown function
NEM-BOFW57_007967	X								4	1	1	1	34.2	7.08	2.9	Unkown function
NEM-BOFW57_009116	X								4	1	1	1	22.6	7.69	1.66	Unkown function
NEM-BOFW57_010273	X			X					6	2	3	2	37.6	7.93	7.7	Unkown function
NEM-BOFW57_010321	X								6	1	1	1	34.2	5.44	2.26	Unkown function
NEM-BOFW57_010708	X								6	1	5	1	27.8	5.6	17.61	Unkown function
NEM-BOFW57_010878	X								3	1	1	1	51.1	5.33	1.73	Unkown function

NEM-BOFW57_000084							X	X	3	1	3	1	47.3	5.5	6.9	CAZYME
NEM-BOFW57_000286						X			2	1	1	1	56.9	6.92	0	CAZYME
NEM-BOFW57_001116								X	3	1	1	1	63.3	8.46	2.17	CAZYME
NEM-BOFW57_001184							X	X	15	2	5	2	26.9	7.94	7.04	CAZYME
NEM-BOFW57_001268						X	X	X	8	2	4	2	50.5	8.7	8.99	CAZYME
NEM-BOFW57_001287							X		3	1	1	1	124.8	5.44	0	CAZYME
NEM-BOFW57_001299			X						1	1	1	1	66.5	6.13	2.15	CAZYME
NEM-BOFW57_001316				X					3	1	1	1	41.7	6.76	2.62	CAZYME
NEM-BOFW57_001968						X			8	1	1	1	26	4.92	2.27	CAZYME
NEM-BOFW57_002736								X	7	1	1	1	34.1	8.66	2.19	CAZYME
NEM-BOFW57_002850			X	X					1	1	3	1	109.7	6.1	1.78	CAZYME
NEM-BOFW57_003172								X	5	1	1	1	33.1	9.01	2.85	CAZYME
NEM-BOFW57_003584				X					6	1	1	1	35.3	6.11	1.96	CAZYME
NEM-BOFW57_003773								X	4	1	1	1	36.3	8.27	0	CAZYME
NEM-BOFW57_004007						X	X	X	7	2	3	2	50.2	7.02	7.39	CAZYME
NEM-BOFW57_004236				X					1	1	1	1	94.6	6.87	1.77	CAZYME
NEM-BOFW57_004480								X	2	1	1	1	101	8.05	0	CAZYME
NEM-BOFW57_004532								X	6	1	1	1	27.7	5.67	2.68	CAZYME
NEM-BOFW57_004738		X	X	X				X	1	1	4	1	132.1	6.51	9.33	CAZYME
NEM-BOFW57_004987				X					2	1	1	1	71.9	4.79	2.31	CAZYME
NEM-BOFW57_005247		X							1	1	1	1	95.3	6.54	2.56	CAZYME
NEM-BOFW57_005284			X					X	2	1	1	1	55.9	7.27	2.05	CAZYME
NEM-BOFW57_005577				X					6	1	1	1	24.1	7.72	0	CAZYME

NEM-BOFW57_005718		X						X	5	1	2	1	26	6.2	5.05	CAZYME
NEM-BOFW57_006599								X	6	1	2	1	37.2	5.83	5.46	CAZYME
NEM-BOFW57_006721			X			X		X	5	2	3	2	54	6.84	8.33	CAZYME
NEM-BOFW57_006926		X	X	X		X	X	X	1	1	6	1	147.8	5.5	15.72	CAZYME
NEM-BOFW57_007690		X	X						5	2	3	2	55.5	7.69	6.14	CAZYME
NEM-BOFW57_007936								X	8	1	1	1	23.3	8	1.69	CAZYME
NEM-BOFW57_008083								X	4	1	1	1	68.5	8.76	0	CAZYME
NEM-BOFW57_008762							X		3	1	1	1	48.2	5.74	2.25	CAZYME
NEM-BOFW57_009077		X	X	X			X	X	8	3	8	3	45.9	6.87	17.91	CAZYME
NEM-BOFW57_009130		X	X	X					2	1	4	1	57	5.34	4.34	CAZYME
NEM-BOFW57_009222								X	3	2	2	2	83.7	6.23	5.09	CAZYME
NEM-BOFW57_009225								X	6	1	2	1	33.5	8.7	3.8	CAZYME
NEM-BOFW57_009327						X	X	X	4	2	7	2	77.5	5.92	16.94	CAZYME
NEM-BOFW57_009419				X				X	2	1	2	1	46.6	7.53	5.04	CAZYME
NEM-BOFW57_009693		X	X	X			X	X	3	1	5	1	36.3	6.96	11.01	CAZYME
NEM-BOFW57_009932		X							2	1	1	1	70.5	6.55	2.97	CAZYME
NEM-BOFW57_010165				X					3	1	4	1	45.6	6.16	8.18	CAZYME
NEM-BOFW57_010289				X				X	3	1	5	1	71.2	6.7	14.79	CAZYME
NEM-BOFW57_010449							X	X	3	1	2	1	67.1	8.16	3.94	CAZYME
NEM-BOFW57_010773		X	X	X		X	X	X	17	3	10	3	32.2	8.92	18.92	CAZYME
NEM-BOFW57_000445					X				5	1	1	1	45	9.1	2.44	Energy metabolism
NEM-BOFW57_000528							X	X	5	1	4	1	38.7	5.58	6.57	Energy metabolism
NEM-BOFW57_000867		X	X	X					5	1	3	1	27	5.54	8.83	Energy metabolism

NEM-BOFW57_004367		X	X		X				3	1	2	1	52.2	8.22	4.02	Energy metabolism
NEM-BOFW57_005119		X							1	1	1	1	72.4	6.32	1.62	Energy metabolism
NEM-BOFW57_007899				X					4	1	1	1	35.4	8.37	2.19	Energy metabolism
NEM-BOFW57_009772		X	X	X					2	1	4	1	73.7	6.47	8.47	Energy metabolism
NEM-BOFW57_009882				X					2	1	2	1	57.8	6.86	5.53	Lipid metabolism
NEM-BOFW57_002979			X						5	1	1	1	23.8	4.94	1.88	NON CONSERVED PATTERN
NEM-BOFW57_003599		X	X						3	1	2	1	34.7	6.76	5.24	NON CONSERVED PATTERN
NEM-BOFW57_006102						X	X		9	1	2	1	17.5	5.95	2.06	NON CONSERVED PATTERN
NEM-BOFW57_006968				X				X	7	1	2	1	15.4	5.92	4.17	NON CONSERVED PATTERN
NEM-BOFW57_007583					X				3	1	1	1	53	8.66	0	NON CONSERVED PATTERN
NEM-BOFW57_008178					X				3	1	1	1	40	6.19	0	NON CONSERVED PATTERN
NEM-BOFW57_008845						X	X		18	1	2	1	7.8	7.42	3.35	NON CONSERVED PATTERN
NEM-BOFW57_009194		X	X	X			X	X	8	1	6	1	19	8.92	13.38	NON CONSERVED PATTERN
NEM-BOFW57_009202					X				1	1	2	1	93	5.03	3.3	NON CONSERVED PATTERN
NEM-BOFW57_009984			X						7	1	1	1	31.4	5.77	0	NON CONSERVED PATTERN
NEM-BOFW57_010651							X	X	10	2	4	2	20.5	6.54	8.87	NON CONSERVED PATTERN
NEM-BOFW57_010657						X			20	1	1	1	16.5	5.08	2.87	NON CONSERVED PATTERN
NEM-BOFW57_000009					X				8	1	2	1	15.7	7.05	5.43	Other biological process
NEM-BOFW57_000226								X	1	1	1	1	159.1	8.34	1.61	Other biological process
NEM-BOFW57_000446			X						1	1	1	1	65.4	5.82	0	Other biological process
NEM-BOFW57_000465					X				3	1	1	1	39.8	6.57	1.84	Other biological process
NEM-BOFW57_000705				X					2	1	2	1	65.7	5.11	4.5	Other biological process
NEM-BOFW57_000721					X				5	1	1	1	34.8	9.41	3.3	Other biological process

NEM-BOFW57_001021		X							4	1	1	1	33.2	9.64	2.74	Other biological process
NEM-BOFW57_001068								X	13	2	3	2	28.9	8	6.33	Other biological process
NEM-BOFW57_001388				X					1	1	1	1	173.6	7.15	1.93	Other biological process
NEM-BOFW57_001465		X	X	X					6	2	5	2	23.5	4.88	11.47	Other biological process
NEM-BOFW57_001931			X						6	1	1	1	26.4	5.77	1.76	Other biological process
NEM-BOFW57_002347						X	X		7	1	2	1	20.6	6.01	5.75	Other biological process
NEM-BOFW57_002353					X				5	1	1	1	23.9	6.14	2.69	Other biological process
NEM-BOFW57_003143		X							10	1	1	1	14.4	4.63	2.62	Other biological process
NEM-BOFW57_003434					X				3	1	1	1	60.2	8.81	2.01	Other biological process
NEM-BOFW57_003740				X					8	1	1	1	48.1	6.74	0	Other biological process
NEM-BOFW57_003752			X						2	1	1	1	55.4	5.83	2.49	Other biological process
NEM-BOFW57_004128				X					4	1	1	1	48.5	7.42	0	Other biological process
NEM-BOFW57_004725				X					3	1	2	1	38.9	6.32	2.14	Other biological process
NEM-BOFW57_005318				X				X	3	1	1	1	71.9	7.21	2.83	Other biological process
NEM-BOFW57_005350					X				2	1	1	1	100.3	8.75	2.07	Other biological process
NEM-BOFW57_005812		X		X			X		17	2	3	2	12.7	5.5	6.59	Other biological process
NEM-BOFW57_005962			X	X					2	1	3	1	65.4	7.52	1.75	Other biological process
NEM-BOFW57_006136		X							10	1	1	1	18.5	8.05	3.15	Other biological process
NEM-BOFW57_006702				X					17	1	2	1	10.5	7.4	4.49	Other biological process
NEM-BOFW57_007040								X	9	1	1	1	18.1	8.15	2.13	Other biological process
NEM-BOFW57_007353					X				4	2	2	2	72.5	6.14	2.02	Other biological process
NEM-BOFW57_009145				X					0	1	1	1	313.8	7.46	1.98	Other biological process
NEM-BOFW57_009769		X							8	1	1	1	19.2	4.77	2.47	Other biological process

NEM-BOFW57_009803			X						3	1	2	1	43.7	6.05	3.7	Other biological process
NEM-BOFW57_009879		X							2	1	1	1	57.7	6.2	0	Other biological process
NEM-BOFW57_009894		X			X		X		5	2	2	2	48.4	4.93	7.72	Other biological process
NEM-BOFW57_009987			X	X			X	X	3	1	3	1	62.9	6.71	7.63	Other biological process
NEM-BOFW57_010270						X			5	1	1	1	15.2	10.35	0	Other biological process
NEM-BOFW57_000642				X					1	1	1	1	116.7	5.85	2.41	Oxidation-reduction
NEM-BOFW57_001246				X					2	1	1	1	81.9	6.42	2.57	Oxidation-reduction
NEM-BOFW57_002570		X	X	X		X	X	X	21	4	15	4	29.2	5.78	47.62	Oxidation-reduction
NEM-BOFW57_005837		X							5	1	1	1	29.1	8.6	2.27	Oxidation-reduction
NEM-BOFW57_005888		X							2	1	1	1	46.9	8.6	2.3	Oxidation-reduction
NEM-BOFW57_007517							X	X	4	1	4	1	51.7	5.71	9.83	Oxidation-reduction
NEM-BOFW57_007667							X		3	1	1	1	68.3	7.59	2.6	Oxidation-reduction
NEM-BOFW57_008312				X					2	1	1	1	64.5	8.03	2.5	Oxidation-reduction
NEM-BOFW57_008362		X							5	1	1	1	33.7	6.04	2.23	Oxidation-reduction
NEM-BOFW57_008741		X		X					3	1	3	1	40.7	7.97	3.67	Oxidation-reduction
NEM-BOFW57_009620			X	X		X			5	2	3	2	25.1	7.75	8.16	Oxidation-reduction
NEM-BOFW57_010142		X	X	X					5	2	6	2	47	6.32	17.73	Oxidation-reduction
NEM-BOFW57_010893			X					X	4	1	2	1	24.7	7.77	3.84	Oxidation-reduction
NEM-BOFW57_000199		X	X	X					11	1	4	1	17	7.64	3.01	Protein metabolism
NEM-BOFW57_000790		X	X	X					6	1	4	1	28.5	8.95	6.66	Protein metabolism
NEM-BOFW57_001158		X							8	1	1	1	20.2	6.8	2.79	Protein metabolism
NEM-BOFW57_001562		X		X					12	1	3	1	16	9.79	7.54	Protein metabolism
NEM-BOFW57_002218			X	X					11	1	2	1	11.1	4.27	5.2	Protein metabolism

NEM-BOFW57_004163		X		X					4	1	2	1	39.4	6.37	4.58	Protein metabolism
NEM-BOFW57_006167						X			33	1	1	1	12.5	4.21	0	Protein metabolism
NEM-BOFW57_006799				X					4	1	1	1	32.5	6.65	2.72	Protein metabolism
NEM-BOFW57_006946					X				3	1	1	1	62.8	6.77	3.14	Protein metabolism
NEM-BOFW57_007373		X	X						1	1	2	1	93.2	6.81	4.25	Protein metabolism
NEM-BOFW57_008773				X					6	1	1	1	24	11.11	3.65	Protein metabolism
NEM-BOFW57_008836						X			6	1	1	1	50.5	6.07	2.13	Protein metabolism
NEM-BOFW57_009768		X		X					5	2	5	2	44.1	10.14	16.48	Protein metabolism
NEM-BOFW57_002046							X	X	5	2	15	2	107.8	6.79	43.07	Proteolysis
NEM-BOFW57_003829						X	X	X	7	2	6	2	42.9	5.17	16.61	Proteolysis
NEM-BOFW57_004704		X		X					6	3	5	3	55	6.7	15.66	Proteolysis
NEM-BOFW57_005294			X						2	1	3	1	32.6	6.57	5.98	Proteolysis
NEM-BOFW57_005333		X		X		X			7	2	3	2	43	5.55	7.9	Proteolysis
NEM-BOFW57_007530					X	X			4	2	2	2	119.6	6.18	8.1	Proteolysis
NEM-BOFW57_009939						X			5	1	1	1	18.3	6.93	2	Proteolysis
NEM-BOFW57_010378				X					7	1	1	1	22.6	7.01	1.84	Proteolysis
NEM-BOFW57_002360		X		X					13	1	2	1	11.3	9.42	4.99	RNA metabolism
NEM-BOFW57_002589		X	X						4	1	2	1	44.8	5.19	4.77	RNA metabolism
NEM-BOFW57_003032				X					5	1	1	1	27.1	5.03	2.31	RNA metabolism
NEM-BOFW57_003040			X						3	1	1	1	36	9.89	0	RNA metabolism
NEM-BOFW57_006500		X							12	1	1	1	14.8	5.31	2.21	RNA metabolism
NEM-BOFW57_007853					X				1	1	5	1	91.7	7.56	11.41	RNA metabolism
NEM-BOFW57_008213					X				2	1	3	1	52.5	6.27	6.3	RNA metabolism

NEM-BOFW57_010150		X							4	1	1	1	41.8	5.95	2.04	RNA metabolism
NEM-BOFW57_010507		X		X					6	2	4	2	43.4	6.73	11.36	RNA metabolism
NEM-BOFW57_000049			X						1	1	1	1	132.2	5.19	1.98	Unkown function
NEM-BOFW57_000139			X						2	1	1	1	80.2	5.47	0	Unkown function
NEM-BOFW57_000843				X					6	1	2	1	16.3	7.03	0	Unkown function
NEM-BOFW57_000863					X				1	1	1	1	146.2	6.21	2.47	Unkown function
NEM-BOFW57_001651						X		X	20	1	2	1	7.6	8.47	4.33	Unkown function
NEM-BOFW57_001677				X		X	X	X	4	1	3	1	30.5	5.2	6.13	Unkown function
NEM-BOFW57_002187				X					15	1	1	1	11.9	7.12	3.39	Unkown function
NEM-BOFW57_002259					X				2	1	1	1	66.5	6.29	2.4	Unkown function
NEM-BOFW57_003190						X	X		4	1	3	1	37.6	4.98	9.36	Unkown function
NEM-BOFW57_003427					X				2	1	1	1	51	4.58	2.19	Unkown function
NEM-BOFW57_005908					X				15	1	1	1	13.9	9.42	3.34	Unkown function
NEM-BOFW57_006192						X		X	14	3	3	3	25	8.81	5.67	Unkown function
NEM-BOFW57_006994				X					11	1	1	1	24	5.3	0	Unkown function
NEM-BOFW57_008609						X	X	X	3	1	6	1	66.1	6.09	8.22	Unkown function
NEM-BOFW57_008753				X					9	1	1	1	20	7.05	2.31	Unkown function
NEM-BOFW57_009140				X					3	1	1	1	35.1	7.03	1.96	Unkown function
NEM-BOFW57_010307						X	X	X	6	2	8	2	43.3	6.13	16.57	Unkown function

Supplementary Table S7. Quantity of reducing sugars released by the commercial Accellerase 1500 (ACC) enzyme cocktail and the FW57 mixture, a 1:1 ratio of secretomes captured following SSF for 21 days on maize and steam-exploded sugarcane. The two preparation were tested on three different forms of biomass: steam-exploded sugarcane bagasse (XSCB), untreated (*in nature*) sugarcane bagasse (NSCB) and untreated maize leaves (MZ).

Time [h]	NSCB/ACC	XSCB/ACC	MZ/ACC	NSCB/FW57	XSCB/FW57	MZ/FW57
**0**	0.00	0.00	0.00	0.00	0.00	0.00

**12**	14.32 ± 0.03	24.15 ± 0.41	8.57 ± 0.09	9.36 ± 0.02	9.12 ± 0.03	10.17 ± 0.22
**24**	17.99 ± 0.20	38.08 ± 0.35	15.52 ± 0.19	11.16 ± 0.04	15.76 ± 0.23	15.76 ± 0.13
**48**	16.33 ± 0.27	48.27 ± 0.41	23.41 ± 0.03	15.80 ± 0.03	29.21 ± 0.06	15.53 ± 0.22

References

Brandt SC, Ellinger B, van Nguyen T, Thi QD, van Nguyen G, Baschien C, Yurkov A, Hahnke RL, Schäfer W, Gand M (2018) A unique fungal strain collection from Vietnam characterized for high performance degraders of bioecological important biopolymers and lipids. PLOS ONE 13(8), doi.org/10.1371/journal.pone.0202695

Ferrari, A.R.; Gaber, Y.; Fraaije, M.W. A fast, sensitive and easy colorimetric assay for chitinase and cellulase activity detection. *Biotechnol. Biofuels* **2014**, *7*, doi:10.1186/1754-6834-7-37.

White TJ, Bruns T, Lee S, Taylor J (1990) Amplification and direct sequencing of fungal ribosomal RNA genes for phylogenetics. In: Innis M, Gelfand D, Sninsky J, White T, editors. PCR Protocols: a Guide to Methods and Applications. Orlando, Florida: Academic Press. pp. 315-322.

Zhang, H.; Yohe, T.; Huang, L.; Entwistle, S.; Wu, P.Z.; Yang, Z.L.; Busk, P.K.; Xu, Y.; Yin, Y.B. dbCAN2: A Meta Server for Automated Carbohydrate-Active Enzyme Annotation. *Nucleic Acids Res* **2018**, *46*, W95-W101, doi:10.1093/nar/gky418.
